# Defining the concepts of a smart nursing home and its potential technology utilities that integrate medical services and are acceptable to stakeholders: a scoping review

**DOI:** 10.1186/s12877-022-03424-6

**Published:** 2022-10-07

**Authors:** Yuanyuan Zhao, Fakhrul Zaman Rokhani, Shariff-Ghazali Sazlina, Navin Kumar Devaraj, Jing Su, Boon-How Chew

**Affiliations:** 1 Department of Family Medicine, Faculty of Medicine & Health Sciences, Universiti Putra Malaysia, 43400 Serdang, Selangor Malaysia; 2 Global Century Science Group, Hong Kong, China; 3 Faculty of Engineering, Universiti Putra Malaysia, Serdang, Malaysia; 4 Malaysian Research Institute on Ageing (MyAgeingTM), Universiti Putra Malaysia, Serdang, Malaysia; 5 College of Public Health, Hainan Medical University, Haikou, China; 6 Clinical Research Unit, Hospital Pengajar Universiti Putra Malaysia (HPUPM Teaching Hospital), Serdang, Malaysia

**Keywords:** Smart nursing homes, Smart technologies, Integration of medical services, Quality of care, Acceptability of stakeholders

## Abstract

**Background and objectives:**

Smart technology in nursing home settings has the potential to elevate an operation that manages more significant number of older residents. However, the concepts, definitions, and types of smart technology, integrated medical services, and stakeholders’ acceptability of smart nursing homes are less clear. This scoping review aims to define a smart nursing home and examine the qualitative evidence on technological feasibility, integration of medical services, and acceptability of the stakeholders.

**Methods:**

Comprehensive searches were conducted on stakeholders’ websites (Phase 1) and 11 electronic databases (Phase 2), for existing concepts of smart nursing home, on what and how technologies and medical services were implemented in nursing home settings, and acceptability assessment by the stakeholders. The publication year was inclusive from January 1999 to September 2021. The language was limited to English and Chinese. Included articles must report nursing home settings related to older adults ≥ 60 years old with or without medical demands but not bed-bound. Technology Readiness Levels were used to measure the readiness of new technologies and system designs. The analysis was guided by the Framework Method and the smart technology adoption behaviours of elder consumers theoretical model. The results were reported according to the PRISMA-ScR.

**Results:**

A total of 177 literature (13 website documents and 164 journal articles) were selected. Smart nursing homes are technology-assisted nursing homes that allow the life enjoyment of their residents. They used IoT, computing technologies, cloud computing, big data and AI, information management systems, and digital health to integrate medical services in monitoring abnormal events, assisting daily living, conducting teleconsultation, managing health information, and improving the interaction between providers and residents. Fifty-five percent of the new technologies were ready for use in nursing homes (levels 6–7), and the remaining were proven the technical feasibility (levels 1–5). Healthcare professionals with higher education, better tech-savviness, fewer years at work, and older adults with more severe illnesses were more acceptable to smart technologies.

**Conclusions:**

Smart nursing homes with integrated medical services have great potential to improve the quality of care and ensure older residents’ quality of life.

**Supplementary Information:**

The online version contains supplementary material available at 10.1186/s12877-022-03424-6.

## Introduction

The ageing population is associated with increased demand in healthcare, and they would require a wide range of assistance in physical mobility and daily monitoring [1]. Smart technologies could help older adults extend their independence and well-being [2]. In the earlier stage, many sensors and actuators were used as a ubiquitous environment (u-healthcare) to monitor patients [3]. IBM’s (International Business Machines Corporation) first introduced the concept of ‘Smarter Planet’ [4], which was briefed as ‘smart’. Later, smart technologies were associated with a range of information technologies such as the Internet of Things (IoT), big data, cloud computing, and artificial intelligence (AI) in the medical field [5]. The World Health Organisation (WHO) (2019) links smart healthcare with digital health, including telemedicine and mobile health (mHealth) [6].

Smart technologies empower older adults to ‘live in place’ and lead their activities to maintain a quality of life [7]. Several studies have proven that smart technologies were feasible to apply in health monitoring, disease prediction, and detection of abnormal situations for home-based care residents [8, 9]. However, admission to nursing homes is usually a significant life event for most older adults due to the changes in health conditions with complex needs in healthcare [10]. Using smart technology in nursing home settings provides residents a more comfortable and safe environment [11]. Nursing homes integrating smart technologies could benefit caregivers by saving time and reducing unnecessary workload while providing efficient and effective care services for residents, such as using wearable devices to collect biometric data [12]. Moreover, it is possible to reduce healthcare costs by using more efficient healthcare resources [13].

Globally, the quality of care in most nursing homes is suboptimal, and the concerns are about the shortages of doctors and nurses, skills of nursing home staff, and safety of medical operations [14–16]. Many nations are seeking solutions for alternative senior care to cope with the challenges of the ageing population and encouraging technique innovation in real-time monitoring of diseases, mobile phone-based healthcare assistance, electronic health record, and telemedicine at nursing homes [17]. As one of the countries in the world facing the ‘grey tsunami’, the Chinese Ministry of Civil Affairs, a nursing home supervision department, initiated a report to promote IoT-based projects for senior institutional care. The Chinee government would financially support the pilot projects in health monitoring, fall detection, location tracking, and any innovation in big data management or analysis [18]. However, a clear concept of technique-assistant nursing home and the appropriate technologies related to ‘smartness’ is yet to be defined [19, 20].

Accordingly, a scoping review is needed to provide a smart nursing home model which includes a definition and the availability of smart technologies to meet the demands and aspirations of potential customers, such as older adults and their family members. Standardising the definition and service scope of smart nursing homes would help introduce appropriate smart technologies in the nursing home settings. A clear concept would also allow stakeholders to evaluate and monitor the operations of smart nursing homes with an evidence-based reference and enhance their acceptability of the smart nursing home model [21].

## Theoretical model

**Fig. 1:**
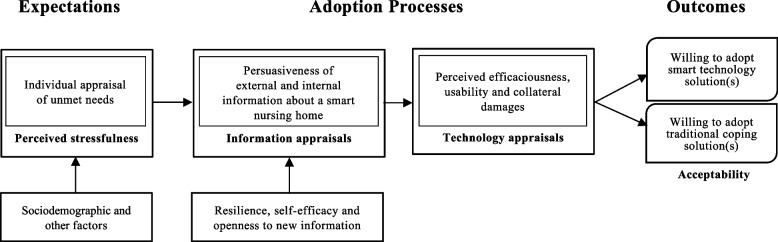
The Smart Technology Adoption Behaviors of Elder Consumer Theoretical Model (Golant, 2017) [22]

The smart technology adoption behaviours of elder consumers theoretical model by Golant (2017) is adopted to guide this scoping review (Fig. 1) [22]. The model offers an adequate explanation of older adults’ coping process regarding adopting smart technologies. The coping process may come from the older adults’ unmet needs in daily life, the user perspective of perceived efficaciousness (usefulness, relative advantage of adoption), usability (easy or complex of use), and collateral damages (unintended harms of use) until deciding to adopt the ‘new’ solution. This coping process is also influenced by internal information (potential users’ past experiences) and external information such as the cues, tips or persuasions of friends, family members, and doctors on the potentials of technology, electronic devices or smart gadgets in daily living. Other factors such as user sociodemographic characteristics may affect their acceptability. The non-senior stakeholders, for example, the healthcare professionals (HCPs), may have the same coping process when considering the older adults’ unmet needs. This model is appropriate for formulating the review objectives.

### Review objectives

This scoping review was conducted to map the concepts of smart nursing homes systematically and to examine the qualitative evidence on technological feasibility, integration of medical services, and the stakeholders’ acceptability of smart nursing homes, including the older adults aged ≥ 60 years old and their caregivers [23].

## Method

Extended and comprehensive searches were conducted on stakeholder websites for existing concepts of smart nursing homes and the criteria of services (Phase 1). The search was continued on the 11 electronic databases for technologies and integrated medical services implemented in nursing home settings, as well as the acceptability as reported by stakeholders, including nursing home residents and HCPs (Phase 2). The eligible articles searched in Phase 2 were included for extracting the definition of smart nursing homes and the criteria of services if they stated the respective information. Technology Readiness Level (TRL) was adopted to evaluate the feasibility and the maturity of a newly developed technology for future implementation [24]. The data analysis was guided by the Framework Method [25] and the smart technology adoption behaviours of elder consumers theoretical model [22]. Results were reported according to the PRISMA-ScR [26] (Supplementary file 1).

### Eligibility criteria

The eligibility criteria include: 1) concepts or definitions of smart nursing home; 2) nursing home residents aged ≥ 60 years old with or without medical demands but not bed-bound; 3) assessment of any health information technologies or models that were considered ‘smartness’ in nursing home settings; 4) perception and acceptability of smart nursing homes by the older adults and other stakeholders; 5) challenges and recommendations to implement information technologies that facilitate medical services in nursing homes. Other articles irrelevant to the study objectives or not in nursing home settings were excluded, for example, the smart technologies applied in home-based settings or technologies used in entertainment, environmental control, and transportation for older adults.

### Information sources and search strategy

Following the plan of the published study protocol [20], the search on stakeholder websites was conducted on three popular search engines for the statement of smart nursing homes, including ‘Google’, ‘Yahoo’ and ‘Baidu (a Chinese engine)’. The search used the following Chinese and English keywords sequentially: ‘Yang Lao Yuan’ (nursing home in Chinese) and followed by ‘smart nursing home’, ‘concept of smart nursing home’, ‘definition of smart nursing home’, ‘criteria of smart nursing home’, and ‘standard of smart nursing home’.

Additionally, the keywords: smart nursing home, smart health*(care), Internet of Things (IoT), digital health*, remote health*(care), telemedicine, mobile health*(care), mHealth (including telemedicine), eHealth, point-of-care, wireless sensor network (WSN), artificial intelligence (AI) and ubiquitous healthcare (u-healthcare) were used for searching the published articles on technological feasibility, integrated medical services, and user acceptability on the English bibliographic databases (PubMed, IEEE Explore, CINAHL, Scopus, Cochrane Library, Health Systems Evidence, Social Systems Evidence, ProQuest Dissertations & Theses Global, Psychology and Behavioral Sciences Collection). The keywords applied on the selected Chinese bibliographic databases (China National Knowledge Infrastructure and the Wanfang Data) were the Chinese description of smart nursing homes, for example, Zhi Neng Yang Lao Yuan, Zhi Hui nursing Yang Lao Yuan, and Yi Liao Kang Yang. The language was limited to English and Chinese. The publication year was limited to those published between January 1999 and May 2020, as the label ‘smart dust technology’ was first introduced in 1999 to describe the limited size of wireless sensor networks and millimeter-scale nodes [27]. Supplementary file 2 provides the search strategy on databases. An updated search was conducted on the 11 bibliographic databases by using the same method to identify the latest publication from May 2020 to September 2021. Due to the license from the university, the search on Scopus was updated to December 2019.

### Selection of sources of evidence

A comprehensive screening of eligible articles was conducted by a reviewer (YYZ). All sources were imported into the Endnotes X9 library, and the duplicates have been removed. Endnotes X9 library was shared with a second reviewer (NKD). Documents in the Chinese language were double reviewed by another reviewer (JS). Eligible criteria were applied to both abstracts and full texts. This scoping review was conducted to provide an overview of the existing evidence of smart nursing home concepts, technological feasibility, integration of medical services, and stakeholders’ acceptability of smart nursing homes regardless of methodological quality or risk of bias [26]. Quality appraisal of reviewed literature and individual source of evidence was not applicable. The third reviewer was involved in the discussion and decided the results when two reviewers had disagreements in the selection process.(FKR, SSG and BHC).

### Data charting

The Framework Method is used to thematically analyse the qualitative data in this scoping review. It is a comparative form of thematic analysis that combines inductive and deductive approaches to analyse texture data and summarise the results, such as using a combination of data description and abstraction (codes and themes) [20]. The data from stakeholder websites and electronic databases were categorised by type of smart technology, technology function, direct user, integrated medical services, and stakeholder acceptability. Three investigators (YYZ, NKD, and JS) extracted the textual statements on the concept of smart nursing homes, implemented technologies, the integration of medical services, and stakeholders’ acceptability. Preliminary codes and themes related to the research objectives were named after the most frequently recurring terms within the same clusters, and the generalisability of textural data gave those names. The codes labelled for stakeholders’ acceptability were referred to the theoretical model [22]. Data extraction and translation from Chinese to English were also done (YYZ). The individual data extraction and analysis were subsequently discussed by all investigators (YYZ, FKR, SSG, and BHC). The coding categories were defined and refined until at least three investigators reached a consensus.

## Results

**Fig. 2:**
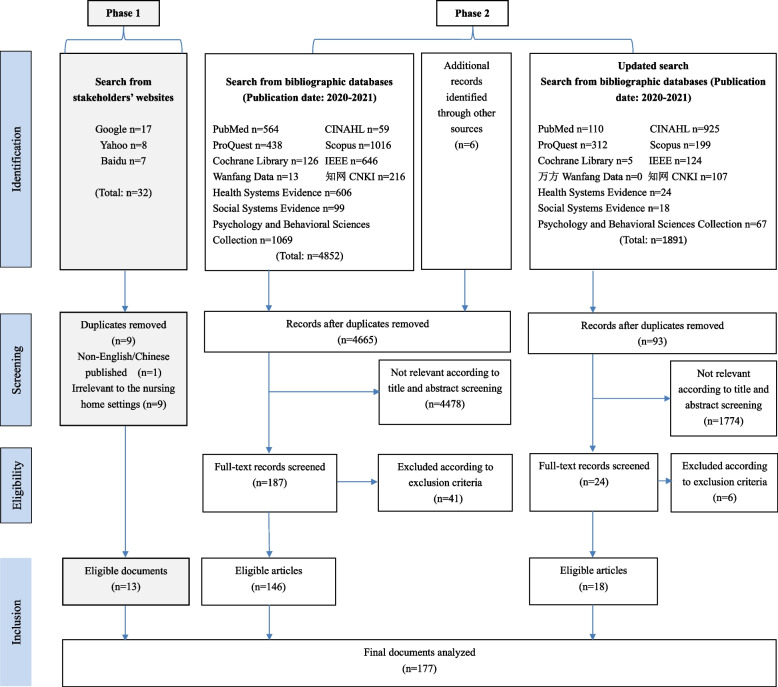
PRISMA Flow Diagram for Scoping Review

A total of 177 pieces of literature (Fig. 2 and supplementary file 3) were selected for review comprising 13 documents from stakeholders’ websites (Phase 1) and 164 articles from bibliographic databases (Phase 2).

### Phase 1: Definition, concepts and criteria of a smart nursing home

Thirty documents and articles (supplementary file 3) were included to retrieve the definitions, concepts, and criteria of smart nursing homes. Of these, there were 13 documents searched from the stakeholder websites in Phase 1 and 17 research papers in Phase 2. The sources of the 13 documents from stakeholder websites were government authorities (*n* = 4), smart technology providers (*n* = 4), home pages of nursing home (*n* = 3), construction company of nursing home (*n* = 1), and respective research institute (*n* = 1).

The qualitative analysis generated three themes related to the concept of smart nursing homes (Table 1): 1) application of smart technologies, 2) technology-assisted nursing care, and 3) combination of smart home and hospital models. In addition, quality of care (QoC) defined by WHO [28] was adopted and applied to measure the criteria and outcome of smart nursing home services that are provided to its residents. In order to achieve better services, health care must be safe, effective, timely, efficient, equitable, and people-centered [28].

**Table 1:** The codes of defining the concepts and criteria of a smart nursing home

Authors and year	Codes	Description	Themes
a. Concept of smart nursing homes			
Baidu, 2018 [29]; Ce.cn, 2019 [30]; Chen & Li, 2012 [31]; Gamberini et al., 2018 [11]; Huang et al., 2019 [12]; Korte [32]; Lee et al., 2018 [33]; Mahieu et al., 2019 [34]; MCA, 2014 [18]; Roh & Park, 2017 [35]; Shenghuo, 2020 [36]; Tang et al., 2019 [37]; Wang, 2014 [19]; Wang, 2020 [38]; Xie, 2017 [39]; Xiexiebang, 2019 [40]; Xu & Tuo, 2019 [41]	IoT^a^	The concept of smartness in nursing home settings is using a new generation of information technologies such as the internet of things (loT), computing technologies, cloud computing, big data and AI, information management system and digital health, to transform traditional nursing care in an all-round way, making healthcare more efficient, more effective, and more personalised	Application of smart technologies (Smartness)
Cui et al., 2020 [42]; Korte [32]; SheCuiTong [43]; Telpo [44]	Computing technologies		
Ce.cn, 2019 [30]	Cloud computing		
Cui et al., 2020 [42]; Mahieu et al., 2019 [34]; MHURD [45]; Telpo [44]; Xu & Tuo, 2019 [41]	Big data and AI^b^		
Baidu, 2018 [29]; Liuye [46]; MHURD [45]; Morley, 2012 [47]	Information management system (IMS)		
BOE Technology Group Co., 2018 [48]; MHURD [45]; Morley, 2012 [47]; Shenghuo, 2020 [36]; Telpo [44]	Digital health		
Shenghuo, 2020 [36]; Siciliano & Khatib, 2016 [49]; Sun et al., 2015 [50]	Assistive devices		
Cui et al., 2020 [42]; Deng, 2019 [51]; MCA, 2014 [18]; Tang et al., 2019 [37]	Intelligent nursing	A nursing home offers technology-assisted nursing care for the people who require a lot of assistance with activities of daily living to improve their quality of life in relation to their goals, expectations, standards and concerns	Technology-assisted nursing care
Korte [32]; Lee et al., 2018 [33]; Xie, 2017 [39]	Automated tracking, monitoring and alerts		
Huang, 2019 [52]; Korte [32]; Wang, 2014 [19]	Improving quality of life		
Baidu, 2018 [29]; Cui et al., 2020 [42]; MHURD [45]; Tang et al., 2019 [37]	Meeting older adults and users' satisfaction		
Cui et al., 2020 [42]; Korte [32]; Morley, 2012 [47]	Similar to smart home	The concept belongs to smart homes with specific users. It performs as a home-based care with the functions of both home and hospital to guarantee a better environment for older adults	Combination of smart home and hospital model
Cui et al., 2020 [42]; Korte [32]	Home and hospital models		
Gamberini et al., 2018 [11]	More comfortable and safe environments		
Cui et al., 2020 [42]; Siciliano & Khatib, 2016 [49]	Special users-older adults and caregivers		
b. Criteria of smart nursing homes			
Baidu, 2018 [29]; Huang et al., 2019 [12]; Korte [32]; Matusitz et al., 2013 [53]; MHURD [45]; Tang et al., 2019 [37]	Provide/improve quality of care	The quality of care is the extent to which health care services provided to individuals and patient populations improve desired health outcomes. In order to achieve this, health care must be safe, effective, timely, efficient, equitable and people-centered. (WHO)	Quality of care
Huang et al., 2019 [12]; MHURD [45]; Siciliano & Khatib, 2016 [49]; Wang, 2020 [38]; Xiexiebang, 2019 [40]	Safe		
Baidu, 2018 [29]; Betgé-Brezetz et al., 2009 [54]; Cui et al., 2020 [42]; MHURD [45]; Shenghuo, 2020 [36]; Tang et al., 2019 [37]	Effective		
Baidu, 2018 [29]; Cui et al., 2020 [42]; SheCuiTong [43]; Siciliano & Khatib, 2016 [49]; Tang et al., 2019 [37]; Xiexiebang, 2019 [40]	Efficient		
Cui et al., 2020 [42]; Huang et al., 2019 [12]; Korte [32]; MHURD [45]; Telpo [44]; Wang, 2014 [19]	People-centered (PC)		

^a^I*oT* Internet of things

^b^ *AI* Artificial intelligence

The qualitative analysis defined a smart nursing home as a collective or individual senior care model. In particular, the smart nursing home integrates the older adults’ daily routine of life and healthcare needs with information technologies or engineering to provide continuous monitoring for its residents, connect communication within its care providers, and conduct teleconsultation with external medical resources. Technology-assisted nursing care ensures life enjoyment in an affordable and safe environment. The smart nursing home services with immediate health attention and people-centered respect are effective, efficient, and evidence-based. Supplementary file 4 presents the quotations and the categories of the code.

### Phase 2: Technological feasibility, integration of medical services and acceptability

A total of 164 articles from 28 countries and regions across four continents were eligible for data extraction. Two of the 164 articles, including an editorial on bringing smart technologies into a nursing home [47] and one system design of engineering methodology [55], were only eligible to be included for exacting the definition of smart nursing homes. There were 162 articles searched in Phase 2 (Table 2) were included to extract the technological feasibility, integration of medical services, and stakeholders’ acceptability. Out of these, 50% (*n* = 81) were studies of system designs, 7% (*n* = 12) experimental, 23% (*n* = 38) non-experimental, 8% (*n* = 13) qualitative studies, 3% (*n* = 4) mixed methods, 9% (*n* = 14) non-research articles including literature reviews, perspective, and editorial. Fifty-seven percent (*n* = 93) were journal articles, 31% (*n* = 50) conference papers, 9% (*n* = 15) student dissertations/theses, and 3% (*n* = 4) book chapters. Most resources were from the USA (*n* = 40) and China, including Taiwan (*n* = 41).

**Table 2:** The codes of smart technologies

No	Authors and year	Country	Type of Publication	Study design	Application	Technologies related to ‘smartness’	Direct User	Function of Technology
1	Suzuki et al., 2006 [56]	Japan	Journal article	System design	Monitoring abnormal events (only location)	IoT	Residents^a^	Monitoring and notification of abnormal events
2	Fischer et al., 2008 [57]	Australia	Conference paper	System design	Monitoring abnormal events	IoT	Residents	
3	Lin et al., 2008 [58]	Taiwan, China	Conference paper	System design	Monitoring abnormal events	IoT	Residents	
4	Betgé-Brezetz et al., 2009 [54]	USA	Conference paper	System design	Notification for specific events	Computing technologies	Residents	
5	Biswas et al., 2009 [59]	Singapore	Book	System design	Monitoring abnormal events (Sleeping monitoring)	IoT	Residents	
6	Hu et al., 2009 [60]	USA	Journal article	System design	Monitoring abnormal events	IoT	NH^b^ staffs	
7	Fraile et al., 2010 [61]	Spain	Conference paper	System design	Monitoring abnormal events	IoT	Residents	
8	Pallikonda Rajasekaran et al., 2010 [62]	India	Journal article	System design	Monitoring abnormal events	IoT	Residents	
9	Gower et al., 2011 [63]	Italy	Conference paper	System design	Monitoring abnormal events	IoT	Residents	
10	Lee et al., 2011 [64]	South Korea	Journal article	System design	Monitoring abnormal events	IoT	Residents	
11	Sun, 2011 [65]	China	Book	System design	Monitoring abnormal events	IoT	Residents	
12	Wu & Huang, 2011 [66]	Taiwan, China	Conference paper	System design	Monitoring abnormal events	IoT	Residents	
13	Back et al., 2012 [67]	Finland	Journal article	System design	Monitoring abnormal events	IoT	Residents	
14	Chang et al., 2012 [68]	Taiwan, China	Journal article	System design	Monitoring abnormal events	IoT	Residents	
15	Chen & Li, 2012 [31]	China	Thesis	System design	Monitoring abnormal events	IoT	Residents	
16	Nijhof et al., 2012 [69]	Netherlands	Journal article	Mixed methods	Monitoring abnormal events (Sleep/wake rhythm monitoring)	IoT	Residents	
17	Ghorbel et al., 2013 [70]	France	Journal article	System design	Notification for specific events	Computing technologies	Residents	
18	Huang et al., 2013 [71]	Taiwan, China	Conference paper	System design	Monitoring abnormal events	IoT	Residents	
19	Matsui et al., 2013 [72]	USA	Journal article	System design	Monitoring abnormal events	Computing technologies	Residents	
20	Neuhaeuser & D'Angelo, 2013 [73]	Germany	Conference paper	System design	Monitoring abnormal events	IoT	Residents	
21	Pan, 2013 [74]	China	Thesis	System design	Monitoring abnormal events	IoT	Residents	
22	Tseng et al., 2013 [75]	USA	Journal article	System design	Monitoring abnormal events	IoT	Residents	
23	Abbate et al., 2014 [76]	Italy	Journal article	Experimental^c^	Fall detection	IoT	Residents	
24	Chu et al., 2014 [77]	China	Journal article	System design	Monitoring abnormal events	IoT	Residents	
25	Liu & Hsu, 2014 [78]	Taiwan, China	Journal article	System design	Monitoring abnormal events (Smart mattress)	IoT	Residents	
26	Wang, 2014 [19]	China	Thesis	System design	Monitoring abnormal events	IoT	Residents	
27	Zhu et al., 2014 [79]	Japan	Conference paper	System design	Monitoring abnormal events (Sleep monitoring)	IoT	Residents	
28	Andò et al., 2015 [80]	Italy	Conference paper	System design	Monitoring abnormal events	IoT	Residents	
29	Carvalho et al., 2015 [81]	France	Conference paper	System design	Monitoring abnormal events	IoT	Residents	
30	Yu et al., 2015 [82]	UK	Conference paper	System design	Monitoring abnormal events	IoT	Residents	
31	Danielsen, 2016 [83]	Norway	Journal article	System design	Monitoring abnormal events	IoT	Residents	
32	Dias et al., 2016 [84]	Brazil	Conference paper	System design	Fall detection	IoT	Residents	
33	Lopez-Samaniego & Garcia-Zapirain, 2016 [85]	Spain	Journal article	System design	Monitoring abnormal events	IoT	Residents	
34	Ansefine et al., 2017 [86]	Indonesia	Conference paper	System design	Monitoring abnormal events	IoT	Residents	
35	Jiang, 2017 [87]	China	Thesis	System design	Monitoring abnormal events	IoT	Residents	
36	Mendes et al., 2017 [88]	Portugal	Conference paper	System design	Monitoring abnormal events	Big data and AI	Residents	
37	Mendoza et al., 2017 [89]	Philippines	Conference paper	System design	Monitoring abnormal events	IoT	Residents	
38	Montanini et al., 2017 [90]	Italy	Conference paper	System design	Monitoring abnormal events (Night monitoring of patients with dementia)	IoT	Residents	
39	Saod et al., 2017 [91]	Malaysia	Conference paper	System design	Monitoring abnormal events	IoT	Residents	
40	Singh et al., 2017 [92]	Austria	Conference paper	Qualitative	Monitoring abnormal events	IoT	Residents	
41	Wu et al., 2017 [93]	China	Conference paper	System design	Monitoring abnormal events	Computing technologies	Residents	
42	Xie, 2017 [39]	China	Thesis	System design	Monitoring abnormal events	Big data and AI	Residents	
43	Bleda et al., 2018 [94]	Spain	Conference paper	System design	Monitoring abnormal events (Smart mattress)	IoT	Residents	
44	Donnelly et al., 2018 [95]	Ireland	Journal article	Qualitative	Fall detection	IoT	Residents	
45	Gamberini et al., 2018 [11]	Italy	Book	Non-research article^d^	Monitoring abnormal events	IoT	Residents	
46	Lee et al., 2018 [33]	South Korea	Conference paper	System design	Monitoring abnormal events	IoT	Residents	
47	Mahfuz et al., 2018 [96]	Canada	Conference paper	System design	Fall detection	IoT	Residents	
48	Morita et al., 2018 [97]	Japan	Conference paper	System design	Monitoring abnormal events	Big data and AI	Residents	
49	Wu et al., 2018 [98]	China	Journal article	System design	Monitoring abnormal events	IoT	Residents	
50	Borelli et al., 2019 [99]	Italy	Journal article	System design	Monitoring abnormal events	IoT	Residents	
51	Cai & Wang, 2019 [100]	China	Journal article	System design	Fall detection	IoT	Residents	
52	Delmastro et al., 2019 [101]	Italy	Journal article	Experimental	Monitoring abnormal events	Cloud computing	Residents	
53	Deng, 2019 [51]	China	Thesis	System design	Monitoring abnormal events	IoT	Residents	
54	Fong et al., 2019 [102]	USA	Conference paper	System design	Monitoring abnormal events	IoT	Residents	
55	Ghosh et al., 2019 [103]	India	Conference paper	System design	Monitoring abnormal events	Big data and AI	Residents	
56	Huang, 2019 [52]	China	Thesis	System design	Fall detection	Big data and AI	Residents	
57	Huang et al., 2019 [12]	Taiwan, China	Conference paper	System design	Monitoring abnormal events	IoT	Residents	
58	Lenoir, 2019 [104]	Japan	Conference paper	System design	Monitoring abnormal events	IoT	Residents	
59	Shen, 2019 [105]	China	Journal article	System design	Monitoring abnormal events	IoT	Residents	
60	Takahashi et al., 2019 [106]	Japan	Conference paper	System design	Monitoring abnormal events (only location)	IoT	Residents	
61	Tang et al., 2019 [37]	China	Journal article	System design	Monitoring abnormal events	IoT	Residents	
62	Toda & Shinomiya, 2019 [107]	Japan	Conference paper	System design	Fall detection	IoT	Residents	
63	Xiao, 2019 [108]	China	Thesis	System design	Monitoring abnormal events (Smart mattress)	IoT	Residents	
64	Xu & Tuo, 2019 [108]	China	Journal article	Non-research article	Monitoring abnormal events	IoT	Residents	
65	Yoo et al., 2019 [109]	South Korea	Conference paper	System design	Monitoring abnormal events	IoT	Residents	
66	Buisseret et al. 2020 [110]	Belgium	Journal article	System design	Fall prediction	Big data and AI	Residents	
67	Chen et al. 2021 [111]	China	Conference paper	System design	Fall prediction	Big data and AI	Residents	
68	Gharti 2020 [112]	Australia	Conference Paper	Non-research article	Fall detection	IoT	Residents	
69	Lanza et al. 2020 [113]	Italy	Journal article	System design	Monitoring abnormal events	Big data and AI	Residents	
70	Lee et al. 2020 [114]	South Korea	Journal article	System design	Fall prediction	Big data and AI	HCPs^e^	
71	Mishkhal et al. 2020 [115]	Iraq	Conference paper	System design	Fall prediction	IoT	Residents	
72	Suzuki et al. 2020 [56]	Japan	Journal article	Non-experimental^f^	Fall prediction	Big data and AI	Residents	
73	Wang, 2020 [38]	China	Thesis	System design	Monitoring abnormal events	IoT	Residents	
74	Wan et al. 2021 [116]	China	Journal article	System design	Fall detection	IoT	Residents	
75	Chen et al. 2021 [111, 117]	Taiwan, China	Conference paper	System design	Monitoring abnormal events	IoT	Residents	
76	Flores-Martin et al. 2021 [118]	Spain	Journal article	System design	Monitoring abnormal events	IoT	Residents	
77	Chan et al., 2001 [119]	China	Journal article	Non-experimental	Telemedicine	Digital health	Residents	Remote clinical services through digital health
78	Pallawala & Lun, 2001 [120]	Singapore	Journal article	Non-experimental	Telemedicine	Digital health	Residents	
79	Weiner et al., 2001 [121]	USA	Journal article	Experimental	Telemedicine	Digital health	Residents	
80	Hui & Woo, 2002 [122]	China	Journal article	Non-experimental	Telemedicine	Digital health	Residents	
81	Savenstedt et al., 2002 [123]	Sweden	Journal article	Qualitative	Telemedicine	Digital health	Residents	
82	Weiner et al., 2003 [124]	USA	Conference paper	Experimental	Telemedicine	Digital health	Residents	
83	Zelickson, 2003 [125]	USA	Journal article	Non-experimental	Telemedicine	Digital health	Residents	
84	Armer et al., 2004 [126]	USA	Journal article	Experimental	Telemedicine	Digital health	Residents	
85	Savenstedt et al., 2004 [127]	Sweden	Journal article	Qualitative	Telemedicine	Digital health	Residents	
86	Daly et al., 2005 [128]	USA	Journal article	Non-research article	Telemedicine	Digital health	Residents	
87	Lavanya et al., 2006 [129]	Singapore	Conference paper	Non-experimental	Teledermatology (Clinical assessment system)	Digital health	Nurses and dermatologists	
88	Loeb et al., 2006 [130]	Canada	Journal article	Non-experimental	Telemedicine (Mobile x-ray)	Digital health	Residents	
89	Shulman et al., 2006 [131]	Canada	Conference paper	Non-research article	Telemedicine	Digital health	Residents	
90	Cusack et al., 2008 [132]	USA	Journal article	Non-experimental	Telemedicine	Digital health	Residents	
91	Janardhanan et al., 2008 [133]	Singapore	Journal article	Non-experimental	Telemedicine	Digital health	Residents	
92	Biglan et al., 2009 [134]	USA	Journal article	Qualitative	Telemedicine	Digital health	Residents	
93	Chang et al., 2009 [135]	Taiwan, China	Journal article	Non-experimental	Telemedicine	Digital health	Residents	
94	Qadri et al., 2009 [136]	USA	Journal article	Mixed methods	Telemedicine (Clinical assessment system)	Digital health	Nurses	
95	Chang et al., 2010 [137]	Taiwan, China	Journal article	Non-experimental	Telemedicine	Digital health	Residents	
96	Rabinowitz et al., 2010 [138]	USA	Journal article	Non-experimental	Telemedicine	Digital health	Residents	
97	Wälivaara et al., 2011 [139]	Sweden	Journal article	Qualitative	Telemedicine	Digital health	Residents	
98	Eklund et al., 2012 [140]	Sweden	Journal article	Non-experimental	Telemedicine (Mobile X-ray)	Digital health	Residents	
99	Gray et al., 2012 [141]	Australia	Journal article	Non-experimental	Telemedicine	Digital health	Residents	
100	Handler et al., 2013 [142]	USA	Journal article	Non-experimental	Telemedicine	Digital health	Residents	
101	Novak et al., 2013 [143]	USA	Conference paper	Experimental	Telemedicine	Digital health	Residents	
102	Vowden & Vowden, 2013 [144]	UK	Journal article	Experimental	Telemedicine	Digital health	Residents	
103	Catic et al., 2014 [145]	USA	Journal article	Non-experimental	Telemedicine	Digital health	Residents	
104	Grabowski & O'Malley, 2014 [146]	USA	Journal article	Experimental	Telemedicine	Digital health	Residents	
105	Crotty et al., 2014 [147]	Australia	Journal article	Experimental	Telemedicine	Digital health	Residents	
106	Doumbouya et al., 2015 [148]	France	Journal article	System design	Telemedicine	Digital health	Residents	
107	F. Huang et al., 2015 [149]	Taiwan, China	Journal article	Experimental	Telemedicine	Digital health	Residents	
108	Montalto et al., 2015 [150]	Australia	Conference paper	Non-experimental	Telemedicine (Mobile X-ray)	Digital health	Residents	
109	Toh et al., 2015b [151]	Singapore	Conference paper	Qualitative	Telemedicine	Digital health	Residents	
110	Toh et al., 2015a [152]	Singapore	Conference paper	Non-experimental	Telemedicine	Digital health	Residents	
111	Volicer, 2015 [153]	USA	Journal article	Non-research article	Telemedicine	Digital health	Residents	
112	De Luca et al., 2016 [154]	Italy	Journal article	Experimental	Telemedicine	Digital health	Residents	
113	Dozet et al., 2016 [155]	Sweden	Journal article	Non-experimental	Telemedicine (Mobile X-ray)	Digital health	Residents	
114	Driessen et al., 2016 [156]	USA	Journal article	Non-experimental	Telemedicine	Digital health	Residents	
115	Gaglio et al., 2016 [157]	France	Conference paper	Qualitative	Telemedicine	Digital health	Residents	
116	Gillespie et al., 2016 [158]	USA	Journal article	Non-experimental	Telemedicine	Digital health	Residents	
117	Morley, 2016 [159]	USA	Journal article	Non-research article	Telemedicine	Digital health	Residents	
118	Schneider et al., 2016 [160]	USA	Journal article	Non-experimental	Telemedicine	Digital health	Residents	
119	Kjelle & Lysdahl, 2017 [161]	Norway	Journal article	Non-research article	Telemedicine (Mobile X-ray)	Digital health	Residents	
120	Newbould et al., 2017 [162]	UK	Book	Non-experimental	Telemedicine	Digital health	Residents	
121	Queyroux et al., 2017 [163]	France	Journal article	Non-experimental	Telemedicine	Digital health	Residents	
122	Delmastro et al., 2018 [101, 164]	Italy	Conference paper	Non-experimental	Telemedicine	Digital health	Residents	
123	Kjelle et al., 2018 [165]	Norway	Journal article	Qualitative	Telemedicine (Mobile X-ray)	Digital health	Residents	
124	Esteves et al., 2019 [166]	Portugal	Journal article	System design	Telemedicine	Digital health	HCPs	
125	Gentry et al., 2019 [167]	USA	Journal article	Non-research article	Telemedicine	Digital health	Residents	
126	Ozkaynak et al., 2019 [168]	USA	Journal article	Qualitative	Telemedicine(Clinical assessment system)	Digital health	NH staffs	
127	Shafiee Hanjani et al., 2019 [169]	Australia	Journal article	Mixed methods	Telemedicine	Digital health	Residents	
128	Cormi et al. 2020 [170]	France	Journal article	Non-research article	Telemedicine	Digital health	Residents	
129	Lai et al. 2020 [171]	USA	Journal article	Non-experimental	Teleophthalmology	Digital health	Residents	
130	Low et al. 2020 [172]	Singapore	Journal article	Non-experimental	Telemedicine	Digital health	Residents	
131	Ohligs et al. 2020 [173]	Germany	Journal article	Non-experimental	Telemedicine	Digital health	Residents	
132	Alexander et al. 2021 [174]	USA	Journal article	Non-experimental	Telemedicine	Digital health	Residents	
133	Okamoto et al. 2021 [175]	USA	Conference paper	Non research article	Telemedicine	Digital health	Residents	
134	Lenderink & Egberts, 2004 [176]	Netherlands	Journal article	Non-experimental	Information management and decision making	IMS^g^	Nurses	Information management and decision making
135	Alexander, 2005 [177]	USA	Thesis	Non-experimental	Information management and decision making	IMS	Administrative staffs	
136	Byrne, 2005 [178]	USA	Thesis	Experimental	Information management and decision making	IMS	NH staffs	
137	Celler et al., 2006 [179]	Australia	Conference paper	Non-experimental	Information management and decision making	IMS	NH staffs	
138	Cherry, 2006 [180]	USA	Thesis	Qualitative	Information management and decision making	IMS	HCPs	
139	Alexander et al., 2007 [181]	USA	Journal article	Qualitative	Information management and decision making	IMS	NH staffs	
140	Alexander, 2008 [182]	USA	Journal article	Non-experimental	Information management and decision making	IMS	NH staffs	
141	Breen & Zhang, 2008 [183]	USA	Journal article	Non-research article	Information management and decision making	IMS	Nurses and other medical practitioners	
142	Yu et al., 2008 [184]	China	Journal article	Mixed methods	Information management and decision making	IMS	Caregivers	
143	Sax & Lawrence, 2009 [185]	Australia	Conference paper	System design	Information management and decision making	IMS	Nurses	
144	Scott-Cawiezell et al., 2009 [186]	USA	Journal article	Non-experimental	Information management and decision making	IMS	Practitioners, nursing staffs, medication administrators and NH leadership	
145	Ohol, 2010 [187]	USA	Thesis	System design	Information management and decision making	IMS	Clinical staffs	
146	Matusitz et al., 2013 [53]	USA	Journal article	Non-research article	Information management and decision making	IMS	Healthcare practitioners	
147	Alexander et al., 2015 [188]	USA	Journal article	Qualitative	Information management and decision making	IMS	Clinical staffs	
148	Z. Huang et al., 2015 [189]	China	Journal article	System design	Information management and decision making	IMS	NH staffs and administration	
149	Wang, 2016 [190]	China	Journal article	Non-research article	Information management and decision making	IMS	HCPs and administration	
150	Zhang, 2017 [191]	China	Thesis	System design	Information management and decision making	IMS	Doctors, nurses and caregivers	
151	Xie, 2016 [192]	China	Thesis	System design	Information management and decision making	IMS	Caregivers	
152	Ausserhofer et al. 2021 [193]	Switzerland	Journal article	Non-experimental	Information management and decision making	IMS	Care workers and nurses	
153	Kei Hong et al. 2021 [194]	China	Journal article	Non-experimental	Information management and decision making	IMS	HCPs	
154	Masuda & Numao, 2017 [195]	Japan	Conference paper	System design	Clinical data anaylsis (Diagnosis)	IoT	Residents	Clinical data analysis by AI
155	Roh & Park, 2017 [35]	South Korea	Journal article	System design	Quality of Life measurements	Big data and AI	HCPs	
156	González et al., 2019 [196]	Spain	Journal article	System design	Clinical data anaylsis (frailty and cognition status)	IoT	HCPs	
157	Kokubo & Kamiya, 2019 [197]	USA	Conference paper	Non-experimental	A new signal parameter estimation algorithm for vital signs monitoring	Big data and AI	HCPs	
158	Ambagtsheer et al., 2020 [198]	Australia	Journal article	Non-experimental	Identifying frailty by using artificial intelligence (AI) algorithms	Big data and AI	HCPs	
159	Hsu et al., 2010 [199]	Taiwan, China	Journal article	System design	ADLs assistance (Pillbox)	IoT	Residents	Activities of daily living (ADLs^h^) assistance
160	Chang et al., 2011 [200]	Taiwan, China	Journal article	System design	ADLs assistance (Pillbox)	IoT	Residents	
161	Sun et al., 2015 [50]	China	Journal article	System design	ADLs assistance (Intelligent robot)	Computing technologies	Residents	
162	Tsai et al., 2017 [201]	Taiwan, China	Conference paper	System design	ADLs assistance (Pillbox)	IoT	Residents	

^a^ *Residents* Nursing home residents

^b^ *NH* Nursing home

^c^Experimental study: The intervention or implementation of smart technologies with one or more control variables of the research subjects conducted in nursing home setting to measure or compare the effect of this manipulation on the users or medical outcomes

^d^Non-research article: Non-original research articles such as review, perspective, controversies, and editorial

^e^ *HCPs* Healthcare professionals

^f^Non-experimental study: No control, manipulate or prediction of intervention and implementation of smart technologies, and the conclusion came through the interpretation, observation or interactions

^g^ *IMS* Information management system

^h^ *ADLs* Activities of daily living

### Technologies related to ‘smartness’

Smart technologies offer much more interaction between the nursing home resident and HCPs, enhance safety, and improve the quality of care [11, 202]. Out of 162 articles, 41% articles (*n* = 66) reported on IoT, 35% (*n* = 57) on digital health, 12% (*n* = 20) on information management system (IMS), 8% (*n* = 13) on big data and AI, 3% (*n* = 5) on computing technologies and 1% (*n* = 1) on cloud computing.

### Functions of smart technology in nursing home settings and direct users

Forty-seven percent of included articles (*n* = 76) reported technologies for monitoring and notification of abnormal events, such as health monitoring, fall detection, and location tracking, 35% (*n* = 57) for remote clinical services through digital health, 12% (*n* = 20) for information management and decision making, 3% (*n* = 5) for clinical data analysis by AI approach, and 3% (*n* = 4) for daily living assistance. The direct users of those smart technologies were nursing home residents (*n* = 132) and HCPs (*n* = 30), such as nursing home staff and health professionals in remote hospitals which provided health services for nursing homes. There were none related to family members as the direct users.

#### Monitoring and notification of abnormal events

Monitoring devices have been proven to ensure the safety of the nursing home residents in fall prevention [52, 76, 84, 95, 96, 100, 107, 110–112, 114–116], automatic monitoring of health conditions, and notification of emerging events, such as heart attacks and fatal accidents [11, 12, 19, 31, 33, 37–39, 41, 51, 54, 57–75, 77–83, 85–94, 97–109, 113, 118, 202, 203]. The vital sign of older adults could be collected and recorded by the wearable devices, such as clothes and shoes on nursing home residents [96, 106]. Sensors were installed in the mattresses and rooms to monitor the older adults’ behaviours and sleeping quality, especially used for residents with limited mobility [51, 90]. Biosensors, ultrasonic sensors, infrared sensors, radio frequency identification (RFID), and GPS were mainly used with IoT terminals [71, 77, 83, 87]. Cameras, mobile devices, and personal computers were embedded with sensor networks to assist the real-time monitoring. Family members could also be given access to the real-time monitoring of their senior family members in the nursing homes [95]. Such a solution improved care efficiency and decision-making of nursing home HCPs, especially in managing a large number of nursing home residents with cognitive disorders [94].

#### Remote clinical services through digital health

Digital health, including telemedicine and mHealth, has shown to benefit the older adults in nursing homes in rural areas with good internet or communication coverage [119, 120, 122, 124, 126, 127, 131–134, 136, 137, 139, 141, 146, 148, 149, 151, 152, 154, 156–162, 166, 168, 204]. During the COVID-19 pandemic, telemedicine reduced unnecessary hospitalisation [170, 175]. Digital images of the residents could be transmitted in real-time to hospital specialists, and that enabled the electronic stethoscopes, otoscopes, dermoscopic, dental scopes, and electrocardiograms to be implemented through the internet and live video to assist clinical practices [128]. Telehealth and mHealth were widely applied in managing cognitive disorders [145, 153, 172], dermatologic conditions [125–144], cardiovascular diseases [124, 137, 173], diabetes mellitus [143], rehabilitation of disabilities [147, 202], dentistry [163] and ophthalmology [171] in the distance. The portable X-ray machine attached with mobile devices successfully conducted x-ray for nursing home residents to reduce unnecessary transmission to the hospitals, and the services were of comparable quality to hospital-based examinations [130, 140, 150, 155, 161]. Telemedicine with designed software helped doctors to prescribe medicines remotely and avoid adverse drug events [123, 142].

#### Information management and decision making

There was a growing use of electronic documentation in many nursing homes requiring proper information management for patients’ medical records, nursing projects, care quality assessment, clinical task schedule, and medication records [179, 180, 186, 188]. The health information of nursing home residents was manually collected by nursing home staff or through technology-based devices, such as mobile phones, tablets, personal computers, and sensors to input into the electronic medical records (EMR) systems [182, 187]. The information management systems also improved clinical decision-making by sharing and tracking patients’ medical records and enhanced HCPs’ communication to reduce errors in clinical practices [53, 176–178, 181, 183–185, 187, 189, 191, 193, 194].

#### Clinical data analysis by AI

AI approach helped with health-related parameter analysis and big data management [35, 197]. Using AI to analyse biometric data collected from older adults enabled the identification of potential relationships between parameters and frailty [196, 198]. As an emerging technology, big data analytics, data mining, and classification used in nursing home management would transform the available data into structured knowledge, enhance data reliability, and enable accurate diagnosis, such as detection of disuse syndrome [88].

#### Activities of daily living (ADLs) assistance

Based on the IoT and computing technologies, smart toolkits have been developed to assist older adults with chronic diseases in their activities of daily living, for example, smart pill-boxes with automagical medication reminders, recording, and pill-dispensing that helped them in taking their daily medications to improve medication adherence [199–201]. Humanoid robots were developed to monitor nursing home residents’ activities and ensure their safety in certain areas [50].

### Technology Readiness Level (TRL) measurement

TRL classifies nine levels of developmental stages, from basic principles and technology concepts formulated to the completion and proof of actual system [205]. Of the 81 articles on system designs, three [83, 90, 117] were not able to be evaluated by TRLs because these were only abstracts with inadequate information, 6.5% (*n* = 5) were judged to be at level 1, 15% (*n* = 12) at level 2, 14% (*n* = 11) at level 3, 6.5% (*n* = 5) at level 4, 4% (*n* = 3) at level 5, 19% (*n* = 15) at level 6, and 35% (*n* = 27) at level 7 (Table 3). Among newly developed technologies, 82% (*n* = 64) were applications for health and abnormal events monitoring, fall detection, and notification systems. The remaining 18% (*n* = 14) were related to activities of daily living assistance, information management, big data analysis, and remote clinical services.

**Table 3:** Technology readiness levels

No	Authors and year	Country	Study design	Function of Technology	Technologies related to ‘smartness’	TRLs
1	Sun et al., 2015 [50]	China	System design	Assisting ADLs^a^	Computing technologies	L 1^b^
2	Xie, 2016 [192]	China	System design	Information management and decision making	IMS	
3	Esteves et al., 2019 [166]	Portugal	System design	Telemedicine	Digital health	
4	Shen, 2019 [105]	China	System design	Monitoring abnormal events	IoT	
5	Chen et al. 2021 [117]	Taiwan, China	System design	Monitoring abnormal events	IoT	
6	Lin et al., 2008 [58]	Taiwan, China	System design	Monitoring abnormal events	IoT	L 2^c^
7	Hu et al., 2009 [60]	USA	System design	Monitoring abnormal events	IoT	
8	Ohol, 2010 [187]	USA	System design	Information management and decision making	IMS	
9	Pallikonda Rajasekaran et al., 2010 [62]	India	System design	Monitoring abnormal events	IoT	
10	Wu & Huang, 2011 [66]	Taiwan, China	System design	Monitoring abnormal events	IoT	
11	Ghorbel et al., 2013 [70]	France	System design	Notification for specific events	Computing technologies	
12	Neuhaeuser & D'Angelo, 2013 [73]	Germany	System design	Monitoring abnormal events	IoT	
13	Chu et al., 2014 [77]	China	System design	Monitoring abnormal events	IoT	
14	Z. Huang et al., 2015 [189]	China	System design	Information management and decision making	IMS	
15	Yu et al., 2015 [82]	UK	System design	Monitoring abnormal events	IoT	
16	Roh & Park, 2017 [35]	South Korea	System design	Quality of Life measurements	Big data and AI	
17	Flores-Martin et al. 2021 [118]	Spain	System design	Monitoring abnormal events	IoT	
18	Sun, 2011 [65]	China	System design	Monitoring abnormal events	IoT	L 3^d^
19	Andò et al., 2015 [80]	Italy	System design	Monitoring abnormal events	IoT	
20	Jiang, 2017 [87]	China	System design	Monitoring abnormal events	IoT	
21	Mendes et al., 2017 [88]	Portugal	System design	Monitoring abnormal events	Big data and AI	
22	Wu et al., 2017 [93]	China	System design	Monitoring abnormal events	Computing technology	
23	Mahfuz et al., 2018 [96]	Canada	System design	Fall detection	IoT	
24	Fong et al., 2019 [102]	USA	System design	Monitoring abnormal events	IoT	
25	Ghosh et al., 2019 [103]	India	System design	Monitoring abnormal events	Big data and AI	
26	Huang, 2019 [12, 52]	China	System design	Fall detection	Big data and AI	
27	Xiao, 2019 [108]	China	System design	Monitoring abnormal events	IoT	
28	Lanza et al. 2020 [113]	Italy	System design	Monitoring abnormal events	Big data and AI	
29	Fischer et al., 2008 [57]	Australia	System design	Monitoring abnormal events	IoT	L 4^e^
30	Hsu et al., 2010 [199]	Taiwan, China	System design	Assisting ADLs	IoT	
31	Chang et al., 2011 [200]	Taiwan, China	System design	Assisting ADLs	IoT	
32	Chen & Li, 2012 [31]	China	System design	Monitoring abnormal events	IoT	
33	Pan, 2013 [74]	China	System design	Monitoring abnormal events	IoT	
34	Carvalho et al., 2015 [81]	France	System design	Monitoring abnormal events	IoT	L 5^f^
35	Borelli et al., 2019 [99]	Italy	System design	Monitoring abnormal events	IoT	
36	Mishkhal et al. 2020 [115]	Iraq	System design	Fall prediction	IoT	
37	Sax & Lawrence, 2009 [185]	Australia	System design	Information management and decision making	IMS	L 6^ g^
38	Gower et al., 2011 [63]	Italy	System design	Monitoring abnormal events	IoT	
39	Lee et al., 2011 [64]	South Korea	System design	Monitoring abnormal events	IoT	
40	Wang, 2014 [19]	China	System design	Monitoring abnormal events	IoT	
41	Doumbouya et al., 2015 [148]	France	System design	Telemedicine	Digital health	
42	Dias et al., 2016 [84]	Brazil	System design	Fall detection	IoT	
43	Ansefine et al., 2017 [86]	Indonesia	System design	Monitoring abnormal events	IoT	
44	Saod et al., 2017 [91]	Malaysia	System design	Monitoring abnormal events	IoT	
45	Xie, 2017 [39]	China	System design	Monitoring abnormal events	Big data and AI	
46	Zhang, 2017 [191]	China	System design	Information management and decision making	IMS	
47	Cai & Wang, 2019 [100]	China	System design	Fall detection	IoT	
48	Deng, 2019 [51]	China	System design	Monitoring abnormal events	IoT	
49	Toda & Shinomiya, 2019 [107]	Japan	System design	Fall detection	IoT	
50	Yoo et al., 2019 [109]	South Korea	System design	Monitoring abnormal events	IoT	
51	Wang, 2020 [38]	China	System design	Monitoring abnormal events	IoT	
52	Suzuki et al., 2006 [203]	Japan	System design	Monitoring abnormal events (location)	IoT	L 7^ h^
53	Betgé-Brezetz et al., 2009 [54]	USA	System design	Notification for specific events	Computing technologies	
54	Biswas et al., 2009 [59]	Singapore	System design	Monitoring abnormal events	IoT	
55	Fraile et al., 2010 [61]	Spain	System design	Monitoring abnormal events	IoT	
56	Back et al., 2012 [67]	Finland	System design	Monitoring abnormal events	IoT	
57	Chang et al., 2012 [68]	Taiwan, China	System design	Monitoring abnormal events	IoT	
58	Huang et al., 2013 [71]	Taiwan, China	System design	Monitoring abnormal events	IoT	
59	Matsui et al., 2013 [72]	USA	System design	Monitoring abnormal events	Computing technology	
60	Tseng et al., 2013 [75]	USA	System design	Monitoring abnormal events	IoT	
61	Liu & Hsu, 2014 [78]	Taiwan, China	System design	Monitoring abnormal events in bed	IoT	
62	Zhu et al., 2014 [79]	Japan	System design	Monitoring abnormal events	IoT	
63	Lopez-Samaniego & Garcia-Zapirain, 2016 [85]	Spain	System design	Monitoring abnormal events	IoT	
64	Masuda & Numao, 2017 [195]	Japan	System design	Clinical data anaylsis (diagnosis)	IoT	
65	Mendoza et al., 2017 [89]	Philippines	System design	Monitoring abnormal events	IoT	
66	Tsai et al., 2017 [201]	Taiwan, China	System design	Assisting ADLs	IoT	
67	Bleda et al., 2018 [94]	Spain	System design	Monitoring abnormal events	IoT	
68	Lee et al., 2018 [33]	South Korea	System design	Monitoring abnormal events	IoT	
69	Morita et al., 2018 [97]	Japan	System design	Monitoring abnormal events	Big data and AI	
70	Wu et al., 2018 [98]	China	System design	Monitoring abnormal events	IoT	
71	Huang et al., 2019 [12, 52]	Taiwan, China	System design	Monitoring abnormal events	IoT	
72	González et al., 2019 [196]	Spain	System design	Clinical data anaylsis (frailty and cognition status)	IoT	
73	Lenoir, 2019 [104]	Japan	System design	Monitoring abnormal events	IoT	
74	Takahashi et al., 2019 [106]	Japan	System design	Monitoring abnormal events (location)	IoT	
75	Tang et al., 2019 [37]	China	System design	Monitoring abnormal events	IoT	
76	Buisseret et al. 2020 [110]	Switzerland	System design	Fall prediction	Big data and AI	
77	Lee et al. 2020 [114]	South Korea	System design	Fall prediction	Big data and AI	
78	Wan et al. 2021 [116]	China	System design	Fall detection	IoT	
79	Montanini et al. 2017 [90]	Italy	System design	Monitoring abnormal events	IoT	Not applicable
80	Danielsen 2016 [83]	Norway	System design	Monitoring abnormal events	IoT	
81	Chen et al. 2021 [111, 117]	China	System design	Fall prediction	Big data and AI	

^a^ *ADLs* Activities of daily living

^b^L 1 = Level 1: Basic principles observed and reported

^c^L 2 = Level 2: Technology concept and/or application formulated

^d^L 3 = Level 3: Analytical and experimental critical function and/or characteristic proof-of-concept

^e^L 4 = Level 4: Component and/or breadboard validation in laboratory environment

^f^L 5 = Level 5: Component and/or breadboard validation in relevant environment

^g^L 6 = Level 6: System/sub-system model or prototype demonstration in relevant environment

^h^L 7 = Level 7: System prototype demonstration in relevant environment

### Integration of medical services

Forty-four out of 162 articles reported the integration of medical services in nursing homes. Telemedicine (31/44, 70%), mHealth (10/44, 23%), and clinical information management (3/44, 7%) were used to integrate medical services from distant hospitals and clinical specialists to assist the nursing homes (Table 4 and supplementary file 5).

**Table 4:** The codes of integration of medical services

No	Authors and year	The form of integrated medical services	Sub-codes	Codes
1	Armer et al., 2004 [126]	Telemedicine and videoconferencing or without videoconferencing	Teleconsultation and videoconferencing	Integration of medical services in telemedicine
2	Daly et al., 2005 [128]	Teleconsulting, live video and image transition		
3	Chan et al., 2001 [119]; Hui & Woo, 2002 [122]; Newbould et al., 2017 [162]; Rabinowitz et al., 2010 [138]; Schneider et al., 2016 [160]; Toh et al., 2015 [151, 152]; Weiner et al., 2001 [121]; Weiner et al., 2003 [124]	Videoconferencing and teleconsultation		
4	Biglan et al., 2009 [134]; Grabowski & O'Malley, 2014 [146]	Videoconferencing and telemedicine		
5	Pallawala & Lun, 2001 [120]	Videoconferencing, teleconsultation and electronic medical records		
6	Savenstedt et al., 2004 [127]	Videophones and teleconsultation		
7	Cusack et al., 2008 [132]	Store-and-forward, real-time video, hybrid systems and teleconsultation		
8	Catic et al., 2014 [145]	Video-consultation technology and teleconsultation		
9	Chang et al., 2010 [137]	Telemonitoring plus teleconsultation via videoconferencing	Telemonitoring	
10	De Luca et al., 2016 [154]	Telemonitoring and teleconsulting		
11	Pallikonda Rajasekaran et al., 2010 [62]	Shared health information collected by wireless Sensor Networks (WSNs) and telemonitoring		
12	Zhang, 2017 [191]	Telemonitoring, wearable devices and web-based health information through an App		
13	Delmastro et al., 2019 [101]; Deng, 2019 [51]; Wang, 2014 [19]; Mishkhal et al. 2020 [115]	Telemonitoring and wearable devices		
14	Vowden & Vowden, 2013 [144]; Zelickson, 2003 [144]	Teleconsultation without videoconference (by digital documents)	Teleconsultation and information technologies	
15	Janardhanan et al., 2008 [133]; Low et al. 2020 [172]	Internet (or email) and teleconsultation		
16	Lavanya et al., 2006 [129]	Personal health information management system (D-PHIMS) and teleconsultation		
17	Doumbouya et al., 2015 [148]	Remote specialists and teleconsultation for decision making	Teleconsultation and remote specialist decision making	
18	Shafiee Hanjani et al., 2019 [169]	Telehealth and interprofessional collaboration		
19	Liu & Hsu, 2014 [78]	mhealth (App) and a soft motion-sensing mattress	mHealth and abnormal event monitoring	Integration of medical services through mHealth
20	Mendes et al., 2017 [88]	Wearable devices and m-health personalised monitoring		
21	Delmastro et al., 2018 [164]	Mobile and e-health personalised monitoring services		
22	Donnelly et al., 2018 [95]	Mobile and wearable devices		
23	Montalto et al., 2015 [150]; Dozet et al., 2016 [206]; Esteves et al., 2019 [166]	Mobile and point-of-care (radiography)	mHealth and point-of-care	
24	Wälivaara et al., 2011 [139]	Teleconsultation and mobile distance-spanning technology (MDST)	mHealth and teleconsultation	
25	Crotty et al., 2014 [147]	Teleconsultation via videoconferencing and web-based health information through an App		
26	Lai et al. 2020 [171]	Smartphone-based teleophthalmology platforms		
27	Alexander, 2008 [182]; Alexander et al., 2015 [188]	Information management and clinical practice in different care departments	Clinical information integration	Integration of clinical information
28	Ohol, 2010 [187]	Electronic health record and technology-based devices		

#### Integration of medical services in telemedicine

The integration of medical services was widely used in the field of telemedicine, for example, videoconferencing (16/31, 52%), telemonitoring (8 /31, 26%), information technologies (5/31, 16%), and remote specialist decision making systems (2/31, 6%) have been integrated to overcome the issues of accessibility and timeliness of medical services for nursing home residents. As a form of telemedicine, teleconsultation integrating real-time videoconference was applicable to replace face-to-face consultations in nursing homes through videophones or computers combined with cameras and microphones, and it enhanced clinical efficiency and cost-effectiveness of healthcare delivery [127, 128, 138, 145]. Teleconsultation integrated health monitoring devices, such as mobile phones or smartwatches, provided a telemonitoring service to record heart rate and blood pressure electronically, and it enabled the HCPs to take prompt responses to the older adults’ urgent health conditions in remote nursing homes [19, 51, 62, 115, 154, 191]. Telemedicine integrated computing technologies have been shown to help remote HCPs make good decisions in clinical management after reviewing patients’ digital health records, which were shared through emails or web-based health management systems [125, 133, 144, 172].

#### Integration of medical services through mHealth

Abnormal events monitoring [78, 88, 95, 202], radiography [150, 166, 206], and teleconsultation [139, 147, 171] could be implemented through mobile devices. mHealth personalised nursing home services, improved efficiency in the closer connection between HCPs and nursing home residents, lowered incidences of unnoticed events, and ensured the residents’ quality of life [142]. Mobile devices connected with sensor-based devices enabled HCPs to monitor and interact with older adults in real-time, and abnormal events such as activities related to falls would be reported to prevent [88]. Mobile applications could assist HCPs at point-of-care in scheduling clinical tasks, performing radiography, digitally recording their clinical practices resulting in time-saving and error reduction [166]. Besides, personal mobiles or tablets were used to connect nursing home residents to conduct teleconsultation [139].

#### Integration of clinical information

Integration of clinical information could improve the quality of care in different medical organisations, for example, sharing patients’ clinical information between nursing homes and differently external care departments, such as the department of pathology, pharmacy, physical therapy, and other social agents, increased valuable support for nursing care, enhanced coordination with multiple specialty consultants, and improved administrative practices [187, 188].

### Stakeholders’ acceptability

Guided by the theoretical model proposed by Golant (2017) [22], the scoping review observed both the expected and unexpected reasons related to stakeholders’ acceptability of smart technologies. In addition, individual attributes were associated with the adoption of smart technologies (Table 5 and supplementary file 6).

**Table 5:** The codes of stakeholders’ acceptability

Authors and year	Sub-codes	Description	Codes	Theme
Huang et al., 2013 [71]	Severity of illness	The attributes of older adults include the severity of illness and other individual sociodemographic variables	Attributes of residents	Attributes of residents and HCPs^a^
Armer et al., 2004 [126]	Education attainment	The identified attributes of HCPs include education attainment, clinical working experience and the level of tech-savvy	Attributes of HCPs	
Handler et al., 2013 [142]	Clinical working experience			
Betgé-Brezetz et al., 2009 [54]; Handler et al., 2013 [142]; Janardhanan et al., 2008 [133]	The level of tech-savvy			
Abbate et al., 2014 [76]; Chang et al., 2009 [135]	Awareness from external resources	External information from HCPs, friends, family members, and media sources	Persuasiveness of external information	Coping process for information and technology appraisals
Eklund et al., 2012 [140]; Huang et al., 2015 [149, 189]	User experience of received benefit from using a new technology	People acquire internal information by remembering personal experiences from their earlier experiences and satisfaction	Persuasiveness of internal information	
Chang et al., 2012 [68]; Weiner et al., 2003 [124]; Yu et al., 2008 [184]; Zelickson, 2003 [125]	Achievement of user’s satisfaction			
Betgé-Brezetz et al., 2009 [54]; Bleda et al., 2018 [94]; Delmastro et al., 2019 [101]; Qadri et al., 2009 [136]; Savenstedt et al., 2002 [123]; Wälivaara et al., 2011 [139]	Usefulness	The perceived efficaciousness of smart technologies was linked to the perceived usefulness, performance expectancy, relative advantage and pleasure experience by the users which was instrumental in achieving medical outcomes and meeting personal demands	Perceived efficaciousness	
Alexander et al., 2007 [181]; Alexander et al., 2015 [188]; Handler et al., 2013 [142]; Janardhanan et al., 2008 [133]; Qadri et al., 2009 [136]	Helpfulness and improvement in care efficiency			
Chan et al., 2001 [119]; Rabinowitz et al., 2010 [138]; Weiner et al., 2003 [124]	A better solution in administrative procedures			
Crotty et al., 2014 [147]; Handler et al., 2013 [142]; Lavanya et al., 2006 [129]; Pallawala & Lun, 2001 [120]; Qadri et al., 2009 [136]; Vowden & Vowden, 2013 [144]; Okamoto et al. 2021 [175]	Improvement in quality of care			
Eklund et al., 2012 [140]; Singh et al., 2017 [92]	Assurance of quality of life			
Eklund et al., 2012 [140]	Improvement of healthcare accessibility and availability	The perceived usability includes effort expectancy, perceived ease of use, or perceived behavioral control (21). The usability appraisals depend on the availability or accessibility of these options, necessary for care, easy to understand, learn and use, affordability, compatible, the availability of tech-support during having difficulties of using a product, and “human-centric” designs such as matching preferences of users, portable and enjoyable to use	Perceived usability (positive)	
Toh et al., 2015 [151, 152]; Tseng et al., 2013 [75]	Necessity for care			
Huang et al., 2015 [149, 189]; Janardhanan et al., 2008 [133]; Lavanya et al., 2006 [129]; Ohligs et al. 2020 [173]	Easy to use			
Huang et al., 2013 [71]; Lavanya et al., 2006 [129]; Yu et al., 2008 [184]	User-friendly			
Crotty et al., 2014 [143]; Hui & Woo, 2002 [122]	Convenience			
Abbate et al., 2014 [76]; Borelli et al., 2019 [99]	“Human-centric” designs to fit user lifestyles			
Hui & Woo, 2002 [122]	Affordability			
Rabinowitz et al., 2010 [138]; Yu et al., 2008 [184]	Adequate tech-support and regular training			
Gaglio et al., 2016 [157]	Appropriate domestication of a new technology			
Lavanya et al., 2006 [129]	Unusefulness	The negative perceiveness to the usability appraisals	Perceived usability (negative)	
Huang et al., 2015 [149, 189]	Uncertainty of usefulness			
Fraile et al., 2010 [207]	Not easy to learn			
Delmastro et al., 2019 [101]	Not easy to use			
Alexander, 2005 [177]; Shafiee Hanjani et al., 2019 [169]	The difficulty of resources availability and accessibility			
Byrne, 2005 [178]	Lacking in supportive resources or tech-support			
Alexander et al., 2007 [181]; Huang et al., 2013 [71]	Burden of using technology			
Toh et al., 2015 [151, 152]	Potential medical risks	The collateral damages refer to the unintended and harmful damages	Perceived collateral damages	
Huang et al., 2015 [149, 189]	Sensitivity of technology and errors during the operation			
Chang et al., 2009 [135]	Overall concern of technology			

^a^ *HCPs* Healthcare professionals

#### Persuasiveness of external information and internal information

Older adults became more aware and willing to use new technology when persuaded or compelled by the potential benefit of the technology from external resources, for example, their family members or HCPs [76, 135]. This coping process is also influenced by internal information, such as user-experienced helpfulness, ease of use, and safety features of the technology [140, 149]. These factors resulted in user satisfaction and enhanced positive attitudes to the final adoption of smart technologies [68, 124].

#### Perceived efficaciousness

The nursing home residents who had experienced or perceived the usefulness of smart technologies in meeting their healthcare demands were more accepting of the technologies [54]. Similarly, HCPs perceived helpfulness in assisting care delivery to improve care efficiency increased their acceptability of smart technology, for example, using health information exchange systems efficiently improved doctor-patient communication [188]. Using smart technologies to improve HCPs’ daily routines, enhance medication safety, and deal with the events of emergencies could be a better solution to ensure the quality of care in nursing homes and the older adults’ quality of life [120, 142].

#### Perceived usability (positive and negative)

Smart technology improved access to healthcare for nursing home residents [140]. The users increased their awareness and consideration of adopting smart technologies when they recognised that smart solutions would be necessary for care [75, 152]. The appraisals of new technology on ease of use or ease to learn [129, 133, 149], user-friendly [71, 129, 184], and convenience [122, 147] in the coping process enhanced user acceptability of smart technology. Users also preferred the “human-centric” designs to fit their lifestyles [76, 99]. The affordability of smart technology is one of the considerations in the coping process, for example, the smart solution would be better accepted if the cost was not higher or not more expensive than the conventional care model [76]. HCPs expected adequate tech support and regular training in applying new technology to enhance the user engagement, confidence, and continuous operation [138, 184]. In addition, appropriate domestication of new technology could improve user acceptability [157]. Domestication is a dynamic process when users in various environments adapt and start to use the new technologies [207].

In contrast, the features of unusefulness or uncertainty of using smart technologies in the coping process were reported to affect the user acceptability negatively, such as the unusefulness [129, 149], difficulty in use or to learn [101, 208], and lacking supportive resources [169, 177] or tech-support in applying technologies [178]. Some HCPs perceived new technologies as a burden to disrupt routines or added workloads, and it may cause reducing their time to provide essential nursing care for the residents, for example, initiating a new information system requiring manual input of residents’ health records into the system caused frustrations among the HCPs [71, 181].

#### Perceived collateral damages

Potential medical risks, sensitivity and reliability of technology, errors during the operation, and increased costs were the main concerns that have been reported [135, 149, 152] associated with the unintended and harmful effects of using smart technology [22].

#### Acceptability differs by the attributes of residents and HCPs

Attributes of residents and HCPs were observed to associate with the acceptability and adoption of smart technologies. The attribute of residents identified from the reviewed articles was the severity of illness [71]. The attributes of HCPs in positively accepting smart technologies in nursing homes were higher educational attainment [126], a few of year working experience (younger age), and better tech-savviness [54, 133, 142].

## Discussion

To the best of our knowledge, this is the first scoping review that identified the gaps and scope of evidence on the concept of a smart nursing home, explored the smart technologies in nursing home settings, and described medical services that could be integrated and implemented in nursing homes. We evaluated the feasibility of innovative technologies in development by applying the TRLs. This review has also captured the stakeholders’ acceptability of smart technologies, especially from the perspectives of older adults and HCPs.

**Fig. 3:**
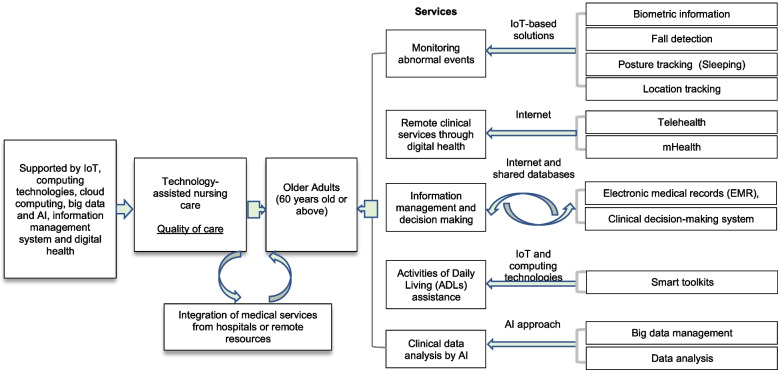
The Concept of a Smart Nursing Home

Previous studies described a smart nursing home as a smart building equipped with IoT technologies [35]. This scoping review concluded that nursing home residents’ health status and emergency situations were mainly monitored and collected by sensors through wearable devices, and the sensors installed on walls less on the user themselves achieved comfort and safe environment [11, 33]. In particular, a smart nursing home would offer technology-assisted nursing care for older adults with the needs of health monitoring, activities of daily living, and safety [37, 209]. Based on their demands, a comprehensive concept of smart nursing homes has to be supported by smart technologies to provide integrated nursing care, personalised monitoring of abnormal events, and assistance in activities of daily living. This smart nursing home model also emphasises the integration of medical services from remote clinical specialists and hospitals to support nursing and medical cares that are convenient, comfortable, and safe to the residents [11]. The services in smart nursing homes could be more effective and efficient in care delivery to achieve the expectations of all stakeholders, including the nursing home residents, family members, and nursing home staff [12]. Figure 3 illustrates the concept of a smart nursing home.

The feasible smart technologies in nursing homes reported in the literature can be classified as IoT, computing technologies, cloud computing, big data and AI, information management system, and digital health. A few published articles classified the most important functions of smart technology in hospital and home-based care settings as health status and mobility monitoring [210]. Hospitals used smart technologies to improve clinical decision-making [21], while in home-based care, smart technologies assisted in the self-management of chronic diseases and remote health monitoring [211, 212]. In nursing homes, the feasible technologies were mainly used in monitoring residents’ abnormal events, connecting to remote clinical services, managing clinical information, analysing big data, and developing device to for the older adults’ to assist their activities of daily living. The TRL evaluation showed that 54% of new system designs were at levels 6 and 7, which have been proven ready for use in nursing homes. The technology function was mainly for monitoring abnormal events in nursing homes. The development of these new technologies is ready to progress to the higher levels of TRL 8 and 9 for commercialisation and future public use. Therefore, the technologies supporting ‘ageing in place’ were developed more maturely, and some of the technologies such as the applications of health monitoring, ADLs, and safety improvement have reached TRL 8 and 9 [209].

Integrating medical services could achieve clinical efficiency and overcome the limited access to healthcare for the older adults who live in rural area [213]. Electronic clinical information, telemedicine, and mHealth have shown the feasibility in overcoming shortages of medical resources and improving healthcare access in nursing homes [169]. The scoping review found that clinical information management and remote clinical services, especially telemedicine, have been broadly implemented in some nursing homes and they were accepted by many stakeholders [147]. With the effective implementation of smart technologies and integration of medical services, many nursing homes could manage a large number of residents and provide customised care to older adults [104].

The theoretical model [22] indicated that the potential users’ persuasiveness of external and internal information, perceived efficaciousness, perceived usability, and perceived collateral damages of using smart technologies determined their acceptability of smart solutions. This scoping review identified and extracted the same determinants from the reviewed articles. In addition, the older adults’ severity of illnesses, the users with a higher level of education and better tech-savviness, and the HCPs with fewer years of working experience (younger age) were associated with higher acceptability of smart technologies [54, 71, 126, 133, 142]. These findings were consistent with the results from a literature review in a home-based care setting. The identified factors that influenced users' technology acceptability included positive experiences with using technologies, such as ease of use, increased safety, security for care, perceived need to use, concerns of technical errors, social influence, and older adults' physical conditions [209]. However, the older adults’ unmet needs and the description of their resilience to smart technology did not mention in the reviewed articles. The older adults did not seem to take concrete actions to adopt a smart technology according to their stressful unmet needs or the different levels of resilience to adversity from the new technologies as indicated in the theoretical model [22].

There are some limitations to be aware of when using the findings in this review. Business reports were not published in the 11 selected databases we searched, and it might cause the review to miss the new technologies or actual systems that have been approved to use in the nursing homes (TRL 8 and 9). Nevertheless, the number and types of databases this review has conducted searches on are believed to have captured informative literature to the review objectives. Meta-analysis and quality assessment were not applicable in this scoping review because the literature and studies informed about the scope and extent of the smart nursing home concepts, technology utilities with its integrated medical services, and acceptability by stakeholders disregarding the literature risk of biases. In the future, researchers could explore the characteristics and feasibility of smart technologies implemented in nursing homes by the particular functions that we categorised, for example, the technologies in the monitoring of abnormal events and activities of daily living assistance.

## Conclusion

Smart nursing homes with integrated medical services have great potential to be a future trend to replace the conventional nursing home. The motivation for transferring from a conventional model to a smart one includes having advanced and safe information technologies, well-trained staff who deliver the nursing care and medical services, and meeting the expectations of all stakeholders. However, technology readiness for frontier technologies, such as clinical data analysis by AI approach and cloud computing technologies, needs to catch up even though much has been presented already, such as the IoT, telemedicine, and information management system. The technology appraisal process was determined by perceived efficaciousness, perceived usability, and perceived collateral damages of adopting the smart technology. Older adults living with severe illnesses and who were persuaded of the benefit of adopting smart solution by the external and internal resources were more accepting of new technologies in nursing homes. Meanwhile, the HCPs with higher educational attainment, fewer years of working experience, and better tech-savviness had higher acceptability of smart technologies.

This scoping review is relevant to a broad base of readers interested in this research and most developed and developing countries with nursing homes. The scoping review results may contribute to future research on introducing smart technologies into nursing homes or developing a successful smart nursing home model. The identified smart technologies that integrate multidisciplinary, such as biomedical informatics and medicine, may provide the technical scope of the smart nursing home model for all stakeholders. The results are also applicable in the planning, evaluating, and monitoring the feasible technologies and service criteria when smart nursing homes are integrated with different types of medical services.

## Supplementary Information

**Additional file 1.** Systematic Reviews and Meta-Analyses (PRISMA) Checklist.

**Additional file 2.** Search Strategy on Databases.

**Additional file 3.** The Retrieved Literature for the Scoping Review.

**Additional file 4.** Code Sheet for Defining the Concepts and Criteria of a Smart Nursing Home.

**Additional file 5.** The Code Sheet of Integration of Medical Services.

**Additional file 6.** The Code Sheet of Stakeholders’ Acceptability.

## Data Availability

The authors declare that all data supporting the findings of this study are already made available in the supplementary files 2–6. If further data clarification is required, please contact the corresponding author or Ms. Yuanyuan Zhao at helenzhao78@qq.com or pcshelenzhao@gmail.com.

## Abbreviations

IoT: Internet of Things
AI: Artificial intelligence
HCPs: Healthcare professionals
TRL: Technology readiness level
ADLs: Activities of daily living
