# Supplementary material for: Defining the concepts of a smart nursing home and its potential technology utilities that integrate medical services and are acceptable to stakeholders: a scoping review
Source: BMC Geriatr. 2022 Oct 7;22:787. doi: 10.1186/s12877-022-03424-6 (PMC9540152; doi:10.1186/s12877-022-03424-6)
Supplement: Supplementary file 2 — Additional file 2. Search Strategy on Databases. [file 12877_2022_3424_MOESM2_ESM.docx]

**Supplementary file 2: Search Strategy on Databases**

| Database | Searched date | Search strategy |
| --- | --- | --- |
| PubMed | 8 September, 2021 | (((((((((((((((smart health[All Fields] OR smart healthcare[All Fields]) AND ("1999/01/01"[PDAT] : "2020/12/31"[PDAT]) AND "humans"[MeSH Terms]) OR (("internet of things"[MeSH Terms] OR ("internet"[All Fields] AND "things"[All Fields]) OR "internet of things"[All Fields]) AND ("1999/01/01"[PDAT] : "2020/12/31"[PDAT]) AND "humans"[MeSH Terms])) OR (IoT[All Fields] AND ("1999/01/01"[PDAT] : "2020/12/31"[PDAT]) AND "humans"[MeSH Terms])) OR ((digital health[All Fields] OR digital healthcare[All Fields]) AND ("1999/01/01"[PDAT] : "2020/12/31"[PDAT]) AND "humans"[MeSH Terms])) OR ((remote health[All Fields] OR remote healthcare[All Fields]) AND ("1999/01/01"[PDAT] : "2020/12/31"[PDAT]) AND "humans"[MeSH Terms])) OR (("telemedicine"[MeSH Terms] OR "telemedicine"[All Fields]) AND ("1999/01/01"[PDAT] : "2020/12/31"[PDAT]) AND "humans"[MeSH Terms])) OR (("telemedicine"[MeSH Terms] OR "telemedicine"[All Fields] OR "ehealth"[All Fields]) AND ("1999/01/01"[PDAT] : "2020/12/31"[PDAT]) AND "humans"[MeSH Terms])) OR ((mobile health[All Fields] OR mobile healthcare[All Fields]) AND ("1999/01/01"[PDAT] : "2020/12/31"[PDAT]) AND "humans"[MeSH Terms])) OR (("telemedicine"[MeSH Terms] OR "telemedicine"[All Fields] OR "mhealth"[All Fields]) AND ("1999/01/01"[PDAT] : "2020/12/31"[PDAT]) AND "humans"[MeSH Terms])) OR (("point-of-care systems"[MeSH Terms] OR ("point-of-care"[All Fields] AND "systems"[All Fields]) OR "point-of-care systems"[All Fields] OR ("point"[All Fields] AND "care"[All Fields]) OR "point of care"[All Fields]) AND ("1999/01/01"[PDAT] : "2020/12/31"[PDAT]) AND "humans"[MeSH Terms])) OR ((wireless[All Fields] AND ("Sensors (Basel)"[Journal] OR "sensor"[All Fields]) AND ("Network"[Journal] OR "Netw Res Triangle Park N C"[Journal] OR "IEEE Netw"[Journal] OR "network"[All Fields])) AND ("1999/01/01"[PDAT] : "2020/12/31"[PDAT]) AND "humans"[MeSH Terms])) OR (("artificial intelligence"[MeSH Terms] OR ("artificial"[All Fields] AND "intelligence"[All Fields]) OR "artificial intelligence"[All Fields]) AND ("1999/01/01"[PDAT] : "2020/12/31"[PDAT]) AND "humans"[MeSH Terms])) OR ((ubiquitous[All Fields] AND ("delivery of health care"[MeSH Terms] OR ("delivery"[All Fields] AND "health"[All Fields] AND "care"[All Fields]) OR "delivery of health care"[All Fields] OR "healthcare"[All Fields])) AND ("1999/01/01"[PDAT] : "2020/12/31"[PDAT]) AND "humans"[MeSH Terms])) AND ("1999/01/01"[PDAT] : "2020/12/31"[PDAT]) AND "humans"[MeSH Terms]) AND (((((((smart[All Fields] AND ("nursing homes"[MeSH Terms] OR ("nursing"[All Fields] AND "homes"[All Fields]) OR "nursing homes"[All Fields] OR ("nursing"[All Fields] AND "home"[All Fields]) OR "nursing home"[All Fields])) AND (("1999/01/01"[PDAT] : "2020/12/31"[PDAT]) AND "humans"[MeSH Terms])) OR ("nursing home"[All Fields] AND (("1999/01/01"[PDAT] : "2020/12/31"[PDAT]) AND "humans"[MeSH Terms]))) OR ((("veterans"[MeSH Terms] OR "veterans"[All Fields] OR "veteran"[All Fields]) AND ("nursing homes"[MeSH Terms] OR ("nursing"[All Fields] AND "homes"[All Fields]) OR "nursing homes"[All Fields] OR ("nursing"[All Fields] AND "home"[All Fields]) OR "nursing home"[All Fields])) AND (("1999/01/01"[PDAT] : "2020/12/31"[PDAT]) AND "humans"[MeSH Terms]))) OR ("old-age home"[All Fields] AND (("1999/01/01"[PDAT] : "2020/12/31"[PDAT]) AND "humans"[MeSH Terms]))) OR ("retirement home"[All Fields] AND (("1999/01/01"[PDAT] : "2020/12/31"[PDAT]) AND "humans"[MeSH Terms]))) OR ((Australian[All Fields] AND residential[All Fields] AND ("aged"[MeSH Terms] OR "aged"[All Fields]) AND care[All Fields] AND home[All Fields]) AND ("1999/01/01"[PDAT] : "2020/12/31"[PDAT]) AND "humans"[MeSH Terms])) AND (("1999/01/01"[PDAT] : "2020/12/31"[PDAT]) AND "humans"[MeSH Terms])  **Updated search applied the**filters:**from 2020 - 2021** |
| CINAHL | 10 September, 2021 | ((((((smart health OR internet of things OR iot OR ( digital health or digital medicine or ehealth or mhealth ) OR remote health care OR telemedicine OR ( mobile health or mhealth or telehealth ) OR point of care OR wireless sensor networks OR artificial intelligence OR ubiquitous healthcare) AND (S3)) AND (S1 OR S2)) AND (S2 AND S3)) AND (S2 AND S3)) AND (S1 AND S2 AND S3)) AND (S4 AND S5)  **Updated search applied the**filters:**from Jan 2020 – Sept 2021** |
| Health Systems Evidence | 8 September, 2021 | smart nursing home NOT child  mobile health* AND nursing home NOT child  **Date range for updated search: 2020-2021** |
| Social Systems Evidence | 8 September, 2021 | smart nursing home NOT child  mobile health* AND nursing home NOT child  **Date range for updated search: 2020-2021** |
| Cochrane Library | 9 September, 2021 | 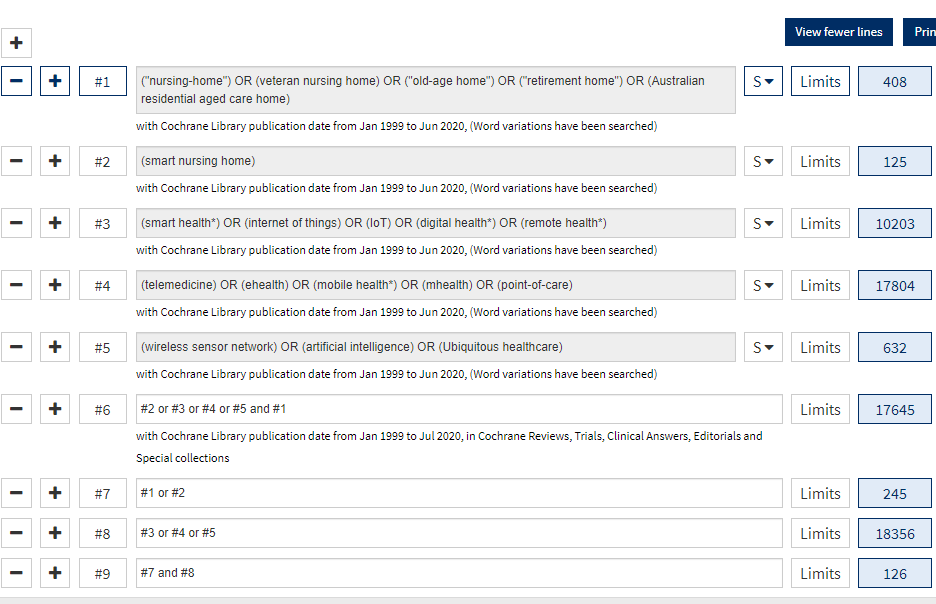  **Updated search applied the**filters: May 2020 to Sept 2021 |
| 万方 Wanfang Data | 9 September, 2021 | 智慧养老院 或智能养老院 或医疗康养养老院  Updated search applied the filters: 9 May 2020 to 9 Sept 2021 |
| 知网 CNKI | 9 September, 2021 | 智慧养老院 或者 智能养老院 或者医疗康养 并含 养老院  Updated search applied the filters: 2020 to Sept 2021 |
| ProQuest Dissertations | 9 September, 2021 | ((("smart health" OR "internet of things" OR "IoT" OR "digital health*" OR "remote health" OR "telemedicine" OR "ehealth" OR "mobile health" OR "mhealth" OR "point-of-care") OR ("wireless sensor network" OR "artificial intelligence" OR "ubiquitous healthcare")) AND ("smart nursing home" OR "nursing home" OR "veteran nursing home" OR "old-age home" OR "retirement home" OR "Australian residential aged care home")) NOT child  Updated search applied the filters: 11 May 2020 to 9 Sept 2021 |
| IEEE | 9 September, 2021 | ((((((((((("Abstract":“smart health”) OR "Abstract":“internet of things”) OR "Abstract":“digital health*”) OR "Abstract":“remote health care”) OR "Abstract":“mobile health”) OR "Abstract":“telehealth”) OR "Abstract":“wireless sensor networks”) OR "Abstract":“artificial intelligence”) OR "Authors":“ubiquitous healthcare”) AND "Full Text & Metadata":“Nursing home”) AND "Full Text & Metadata":elderly)  **Date range for updated search: 2020-2021** |
| **Psychology and Behavioral Sciences Collection** | 9 September, 2021 | 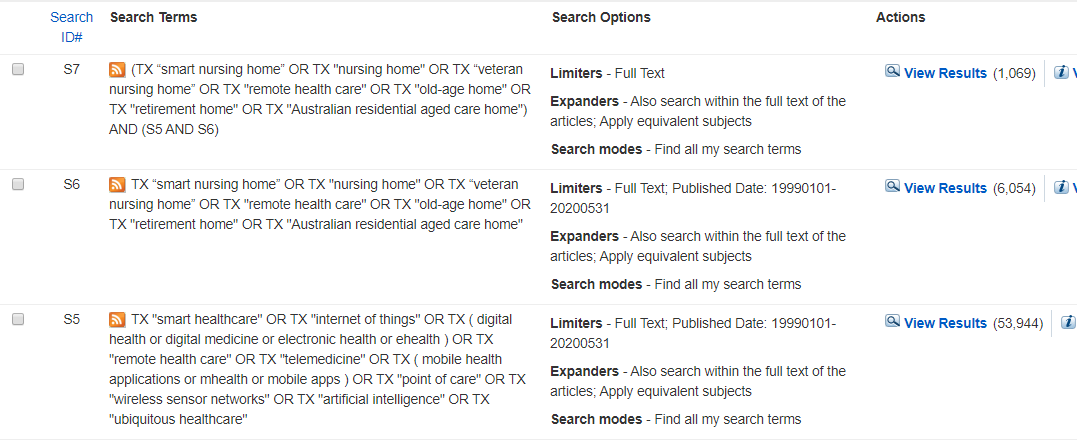  **Date range for updated search: 2020-2021** |
| Scopus | 9 September, 2021 | ( TITLE-ABS-KEY ( "smart health" ) OR TITLE-ABS-KEY ( "internet of things" ) OR TITLE-ABS-KEY ( "IoT" ) OR TITLE-ABS-KEY ( "digital health" ) OR TITLE-ABS-KEY ( "remote health" ) OR TITLE-ABS-KEY ( "telemedicine" ) OR TITLE-ABS-KEY ( "ehealth" ) OR TITLE-ABS-KEY ( "mobile health" ) OR TITLE-ABS-KEY ( "mhealth" ) OR TITLE-ABS-KEY ( "point-of-care" ) OR TITLE-ABS-KEY ( "wireless sensor network" ) OR TITLE-ABS-KEY ( "artificial intelligence" ) OR TITLE-ABS-KEY ( "ubiquitous healthcare" ) OR ALL ( "smart nursing home" ) AND ALL ( "nursing home" ) AND ALL ( "elderly" ) ) AND PUBYEAR > 1998  **Date updated search to: December 2019 (UPM license)** |
