# Supplementary material for: Defining the concepts of a smart nursing home and its potential technology utilities that integrate medical services and are acceptable to stakeholders: a scoping review
Source: BMC Geriatr. 2022 Oct 7;22:787. doi: 10.1186/s12877-022-03424-6 (PMC9540152; doi:10.1186/s12877-022-03424-6)
Supplement: Supplementary file 3 — Additional file 3. The Retrieved Literature for the Scoping Review. [file 12877_2022_3424_MOESM3_ESM.docx]

**Supplementary file 3: The Retrieved Literature for the Scoping Review**

List A. Thirty documents and articles were retrieved for the concepts of a smart nursing home in Phase 1

| No. | Authors and year | Country | Type of document | Stakeholder | Smart concept | Technology-assisted nursing care | Home model | Quality of care |
| --- | --- | --- | --- | --- | --- | --- | --- | --- |
| 1 | Betgé-Brezetz et al., 2009  (1) | USA | Conference paper | X^a^ | X | X | X | Yes |
| 2 | Chen & Li, 2012  (2) | China | Thesis | X | Yes | X | X | X |
| 3 | Morley, 2012  (3) | USA | Journal article | X | Yes | X | Yes | X |
| 4 | Matusitz et al., 2013  (4) | USA | Journal article | X | X | X | X | Yes |
| 5 | MCA, 2014  (5) | China | Web page | Government | Yes | Yes | X | X |
| 6 | Wang, 2014  (6) | China | Thesis | X | Yes | Yes | X | Yes |
| 7 | Sun et al., 2015  (7) | China | Journal article | X | Yes | X | X | X |
| 8 | Siciliano & Khatib, 2016  (8) | Germany | Web Page | Research Institute | Yes | X | Yes | Yes |
| 9 | Roh & Park, 2017  (9) | South Korea | Journal article | X | Yes | X | X | X |
| 10 | Xie, 2017  (10) | China | Thesis | X | Yes | Yes | X | X |
| 11 | Baidu, 2018  (11) | China | Web page | Nursing home | Yes | Yes | X | Yes |
| 12 | BOE Technology Group Co., 2018  (12) | China | Web page | Tech Provider | Yes | X | X | X |
| 13 | Gamberini et al., 2018 (13) | Italy | Book | X | Yes | X | Yes | X |
| 14 | Lee et al., 2018  (14) | South Korea | Conference paper | X | Yes | Yes | X | X |
| 15 | Ce.cn, 2019  (15) | China | Web page | Nursing home | Yes | X | X | X |
| 16 | Deng, 2019  (16) | China | Thesis | X | X | Yes | X | X |
| 17 | Huang, 2019  (17) | China | Thesis | X | X | Yes | X | Yes |
| 18 | Huang et al., 2019  (18) | Taiwan, China | Conference paper | X | Yes | X | X | Yes |
| 19 | Mahieu et al., 2019  (19) | Belgium | Web Page | Government Institute | Yes | X | X | X |
| 20 | Tang et al., 2019  (20) | China | Journal article | X | Yes | Yes | X | Yes |
| 21 | Xiexiebang, 2019  (21) | China | Web page | Government | Yes | X | X | Yes |
| 22 | Xu & Tuo, 2019  (22) | China | Journal article | X | Yes | X | X | X |
| 23 | Cui et al., 2020  (23) | China | Journal article | X | Yes | Yes | Yes | Yes |
| 24 | Shenghuo, 2020  (24) | China | Web page | Nursing home | Yes | X | X | X |
| 25 | Wang, 2020  (25) | China | Thesis | X | Yes | X | X | Yes |
| 26 | Korte  (26) | USA | Web page | Construction company | Yes | Yes | Yes | Yes |
| 27 | Liuye  (27) | China | Web page | Tech Provider | Yes | X | X | X |
| 28 | MHURD  (28) | China | Web page | Government | Yes | Yes | X | Yes |
| 29 | SheCuiTong  (29) | China | Web page | Tech Provider | Yes | X | X | Yes |
| 30 | Telpo  (30) | China | Web page | Tech Provider | Yes | X | X | Yes |

^a^ X=No information was mentioned in the reviewed document

List B. Articles were reviewed for the categories of smart technology in Phase 2

| No. | Authors and year | Country | Type of Publication | Study design | Application | Technologies related to ‘smartness’ | Direct User | Function of Technology |
| --- | --- | --- | --- | --- | --- | --- | --- | --- |
| 1 | Suzuki et al., 2006  (31) | Japan | Journal article | System design | Monitoring abnormal events (only location) | IoT | Residents^a^ | Monitoring and notification of abnormal events |
| 2 | Fischer et al., 2008  (32) | Australia | Conference paper | System design | Monitoring abnormal events | IoT | Residents |  |
| 3 | Lin et al., 2008  (33) | Taiwan, China | Conference paper | System design | Monitoring abnormal events | IoT | Residents |  |
| 4 | Betgé-Brezetz et al., 2009  (1) | USA | Conference paper | System design | Notification for specific events | Computing technologies | Residents |  |
| 5 | Biswas et al., 2009  (34) | Singapore | Book | System design | Monitoring abnormal events  (Sleeping monitoring) | IoT | Residents |  |
| 6 | Hu et al., 2009  (35) | USA | Journal article | System design | Monitoring abnormal events | IoT | NH^b^ staffs |  |
| 7 | Fraile et al., 2010  (36) | Spain | Conference paper | System design | Monitoring abnormal events | IoT | Residents |  |
| 8 | Pallikonda Rajasekaran et al., 2010  (37) | India | Journal article | System design | Monitoring abnormal events | IoT | Residents |  |
| 9 | Gower et al., 2011  (38) | Italy | Conference paper | System design | Monitoring abnormal events | IoT | Residents |  |
| 10 | Lee et al., 2011  (39) | South Korea | Journal article | System design | Monitoring abnormal events | IoT | Residents |  |
| 11 | Sun, 2011  (40) | China | Book | System design | Monitoring abnormal events | IoT | Residents |  |
| 12 | Wu & Huang, 2011  (41) | Taiwan, China | Conference paper | System design | Monitoring abnormal events | IoT | Residents |  |
| 13 | Back et al., 2012  (42) | Finland | Journal article | System design | Monitoring abnormal events | IoT | Residents |  |
| 14 | Chang et al., 2012  (43) | Taiwan, China | Journal article | System design | Monitoring abnormal events | IoT | Residents |  |
| 15 | Chen & Li, 2012  (2) | China | Thesis | System design | Monitoring abnormal events | IoT | Residents |  |
| 16 | Nijhof et al., 2012  (44) | Netherlands | Journal article | Mixed methods | Monitoring abnormal events  (Sleep/wake rhythm monitoring) | IoT | Residents |  |
| 17 | Ghorbel et al., 2013  (45) | France | Journal article | System design | Notification for specific events | Computing technologies | Residents |  |
| 18 | Huang et al., 2013  (46) | Taiwan, China | Conference paper | System design | Monitoring abnormal events | IoT | Residents |  |
| 19 | Matsui et al., 2013  (47) | USA | Journal article | System design | Monitoring abnormal events | Computing technologies | Residents |  |
| 20 | Neuhaeuser & D'Angelo, 2013  (48) | Germany | Conference paper | System design | Monitoring abnormal events | IoT | Residents |  |
| 21 | Pan, 2013  (49) | China | Thesis | System design | Monitoring abnormal events | IoT | Residents |  |
| 22 | Tseng et al., 2013  (50) | USA | Journal article | System design | Monitoring abnormal events | IoT | Residents |  |
| 23 | Abbate et al., 2014  (51) | Italy | Journal article | Experimental^c^ | Fall detection | IoT | Residents |  |
| 24 | Chu et al., 2014  (52) | China | Journal article | System design | Monitoring abnormal events | IoT | Residents |  |
| 25 | Liu & Hsu, 2014  (53) | Taiwan, China | Journal article | System design | Monitoring abnormal events  (Smart mattress) | IoT | Residents |  |
| 26 | Wang, 2014  (6) | China | Thesis | System design | Monitoring abnormal events | IoT | Residents |  |
| 27 | Zhu et al., 2014  (54) | Japan | Conference paper | System design | Monitoring abnormal events  (Sleep monitoring) | IoT | Residents |  |
| 28 | Andò et al., 2015  (55) | Italy | Conference paper | System design | Monitoring abnormal events | IoT | Residents |  |
| 29 | Carvalho et al., 2015  (56) | France | Conference paper | System design | Monitoring abnormal events | IoT | Residents |  |
| 30 | Yu et al., 2015  (57) | UK | Conference paper | System design | Monitoring abnormal events | IoT | Residents |  |
| 31 | Danielsen, 2016  (58) | Norway | Journal article | System design | Monitoring abnormal events | IoT | Residents |  |
| 32 | Dias et al., 2016  (59) | Brazil | Conference paper | System design | Fall detection | IoT | Residents |  |
| 33 | Lopez-Samaniego & Garcia-Zapirain, 2016  (60) | Spain | Journal article | System design | Monitoring abnormal events | IoT | Residents |  |
| 34 | Ansefine et al., 2017  (61) | Indonesia | Conference paper | System design | Monitoring abnormal events | IoT | Residents |  |
| 35 | Jiang, 2017  (62) | China | Thesis | System design | Monitoring abnormal events | IoT | Residents |  |
| 36 | Mendes et al., 2017  (63) | Portugal | Conference paper | System design | Monitoring abnormal events | Big data and AI | Residents |  |
| 37 | Mendoza et al., 2017  (64) | Philippines | Conference paper | System design | Monitoring abnormal events | IoT | Residents |  |
| 38 | Montanini et al., 2017  (65) | Italy | Conference paper | System design | Monitoring abnormal events  (Night monitoring of patients with dementia) | IoT | Residents |  |
| 39 | Saod et al., 2017  (66) | Malaysia | Conference paper | System design | Monitoring abnormal events | IoT | Residents |  |
| 40 | Singh et al., 2017  (67) | Austria | Conference paper | Qualitative | Monitoring abnormal events | IoT | Residents |  |
| 41 | Wu et al., 2017  (68) | China | Conference paper | System design | Monitoring abnormal events | Computing technologies | Residents |  |
| 42 | Xie, 2017  (10) | China | Thesis | System design | Monitoring abnormal events | Big data and AI | Residents |  |
| 43 | Bleda et al., 2018  (69) | Spain | Conference paper | System design | Monitoring abnormal events  (Smart mattress) | IoT | Residents |  |
| 44 | Donnelly et al., 2018  (70) | Ireland | Journal article | Qualitative | Fall detection | IoT | Residents |  |
| 45 | Gamberini et al., 2018  (13) | Italy | Book | Non-research article^d^ | Monitoring abnormal events | IoT | Residents |  |
| 46 | Lee et al., 2018  (14) | South Korea | Conference paper | System design | Monitoring abnormal events | IoT | Residents |  |
| 47 | Mahfuz et al., 2018  (71) | Canada | Conference paper | System design | Fall detection | IoT | Residents |  |
| 48 | Morita et al., 2018  (72) | Japan | Conference paper | System design | Monitoring abnormal events | Big data and AI | Residents |  |
| 49 | Wu et al., 2018  (73) | China | Journal article | System design | Monitoring abnormal events | IoT | Residents |  |
| 50 | Borelli et al., 2019  (74) | Italy | Journal article | System design | Monitoring abnormal events | IoT | Residents |  |
| 51 | Cai & Wang, 2019  (75) | China | Journal article | System design | Fall detection | IoT | Residents |  |
| 52 | Delmastro et al., 2019  (76) | Italy | Journal article | Experimental | Monitoring abnormal events | Cloud computing | Residents |  |
| 53 | Deng, 2019  (16) | China | Thesis | System design | Monitoring abnormal events | IoT | Residents |  |
| 54 | Fong et al., 2019  (77) | USA | Conference paper | System design | Monitoring abnormal events | IoT | Residents |  |
| 55 | Ghosh et al., 2019  (78) | India | Conference paper | System design | Monitoring abnormal events | Big data and AI | Residents |  |
| 56 | Huang, 2019  (17) | China | Thesis | System design | Fall detection | Big data and AI | Residents |  |
| 57 | Huang et al., 2019  (18) | Taiwan, China | Conference paper | System design | Monitoring abnormal events | IoT | Residents |  |
| 58 | Lenoir, 2019  (79) | Japan | Conference paper | System design | Monitoring abnormal events | IoT | Residents |  |
| 59 | Shen, 2019  (80) | China | Journal article | System design | Monitoring abnormal events | IoT | Residents |  |
| 60 | Takahashi et al., 2019  (81) | Japan | Conference paper | System design | Monitoring abnormal events (only location) | IoT | Residents |  |
| 61 | Tang et al., 2019  (20) | China | Journal article | System design | Monitoring abnormal events | IoT | Residents |  |
| 62 | Toda & Shinomiya, 2019  (82) | Japan | Conference paper | System design | Fall detection | IoT | Residents |  |
| 63 | Xiao, 2019  (83) | China | Thesis | System design | Monitoring abnormal events  (Smart mattress) | IoT | Residents |  |
| 64 | Xu & Tuo, 2019  (22) | China | Journal article | Non-research article | Monitoring abnormal events | IoT | Residents |  |
| 65 | Yoo et al., 2019  (84) | South Korea | Conference paper | System design | Monitoring abnormal events | IoT | Residents |  |
| 66 | Buisseret et al., 2020  (85) | Belgium | Journal article | System design | Fall prediction | Big data and AI | Residents |  |
| 67 | Chen et al., 2021  (86) | China | Conference paper | System design | Fall prediction | Big data and AI | Residents |  |
| 68 | Gharti, 2020  (87) | Australia | Conference Paper | Non-research article | Fall detection | IoT | Residents |  |
| 69 | Lanza et al., 2020  (88) | Italy | Journal article | System design | Monitoring abnormal events | Big data and AI | Residents |  |
| 70 | Lee et al., 2020  (89) | South Korea | Journal article | System design | Fall prediction | Big data and AI | HCPs^e^ |  |
| 71 | Mishkhal et al., 2020  (90) | Iraq | Conference paper | System design | Fall prediction | IoT | Residents |  |
| 72 | Suzuki et al., 2020  (91) | Japan | Journal article | Non-experimental^f^ | Fall prediction | Big data and AI | Residents |  |
| 73 | Wang, 2020  (25) | China | Thesis | System design | Monitoring abnormal events | IoT | Residents |  |
| 74 | Wan & Chin, 2021  (92) | China | Journal article | System design | Fall detection | IoT | Residents |  |
| 75 | Chen et al. 2021  (93) | Taiwan, China | Conference paper | System design | Monitoring abnormal events | IoT | Residents |  |
| 76 | Flores-Martin et al., 2021  (94) | Spain | Journal article | System design | Monitoring abnormal events | IoT | Residents |  |
| 77 | Chan et al., 2001  (95) | China | Journal article | Non-experimental | Telemedicine | Digital health | Residents | Remote clinical services through digital health |
| 78 | Pallawala & Lun, 2001  (96) | Singapore | Journal article | Non-experimental | Telemedicine | Digital health | Residents |  |
| 79 | Weiner et al., 2001  (97) | USA | Journal article | Experimental | Telemedicine | Digital health | Residents |  |
| 80 | Hui & Woo, 2002  (98) | China | Journal article | Non-experimental | Telemedicine | Digital health | Residents |  |
| 81 | Savenstedt et al., 2002  (99) | Sweden | Journal article | Qualitative | Telemedicine | Digital health | Residents |  |
| 82 | Weiner et al., 2003  (100) | USA | Conference paper | Experimental | Telemedicine | Digital health | Residents |  |
| 83 | Zelickson, 2003  (101) | USA | Journal article | Non-experimental | Telemedicine | Digital health | Residents |  |
| 84 | Armer et al., 2004  (102) | USA | Journal article | Experimental | Telemedicine | Digital health | Residents |  |
| 85 | Savenstedt et al., 2004  (103) | Sweden | Journal article | Qualitative | Telemedicine | Digital health | Residents |  |
| 86 | Daly et al., 2005  (104) | USA | Journal article | Non-research article | Telemedicine | Digital health | Residents |  |
| 87 | Lavanya et al., 2006  (105) | Singapore | Conference paper | Non-experimental | Teledermatology (Clinical assessment system) | Digital health | Nurses and dermatologists |  |
| 88 | Loeb et al., 2006  (106) | Canada | Journal article | Non-experimental | Telemedicine  (Mobile x-ray) | Digital health | Residents |  |
| 89 | Shulman et al., 2006  (107) | Canada | Conference paper | Non-research article | Telemedicine | Digital health | Residents |  |
| 90 | Cusack et al., 2008  (108) | USA | Journal article | Non-experimental | Telemedicine | Digital health | Residents |  |
| 91 | Janardhanan et al., 2008  (109) | Singapore | Journal article | Non-experimental | Telemedicine | Digital health | Residents |  |
| 92 | Biglan et al., 2009  (110) | USA | Journal article | Qualitative | Telemedicine | Digital health | Residents |  |
| 93 | Chang et al., 2009  (111) | Taiwan, China | Journal article | Non-experimental | Telemedicine | Digital health | Residents |  |
| 94 | Qadri et al., 2009  (112) | USA | Journal article | Mixed methods | Telemedicine (Clinical assessment system) | Digital health | Nurses |  |
| 95 | Chang et al., 2010  (113) | Taiwan, China | Journal article | Non-experimental | Telemedicine | Digital health | Residents |  |
| 96 | Rabinowitz et al., 2010  (114) | USA | Journal article | Non-experimental | Telemedicine | Digital health | Residents |  |
| 97 | Wälivaara et al., 2011  (115) | Sweden | Journal article | Qualitative | Telemedicine | Digital health | Residents |  |
| 98 | Eklund et al., 2012  (116) | Sweden | Journal article | Non-experimental | Telemedicine  (Mobile X-ray) | Digital health | Residents |  |
| 99 | Gray et al., 2012  (117) | Australia | Journal article | Non-experimental | Telemedicine | Digital health | Residents |  |
| 100 | Handler et al., 2013  (118) | USA | Journal article | Non-experimental | Telemedicine | Digital health | Residents |  |
| 101 | Novak et al., 2013  (119) | USA | Conference paper | Experimental | Telemedicine | Digital health | Residents |  |
| 102 | Vowden & Vowden, 2013  (120) | UK | Journal article | Experimental | Telemedicine | Digital health | Residents |  |
| 103 | Catic et al., 2014  (121) | USA | Journal article | Non-experimental | Telemedicine | Digital health | Residents |  |
| 104 | Grabowski & O'Malley, 2014  (122) | USA | Journal article | Experimental | Telemedicine | Digital health | Residents |  |
| 105 | Crotty et al., 2014  (123) | Australia | Journal article | Experimental | Telemedicine | Digital health | Residents |  |
| 106 | Doumbouya et al., 2015  (124) | France | Journal article | System design | Telemedicine | Digital health | Residents |  |
| 107 | Huang et al., 2015  (125) | Taiwan, China | Journal article | Experimental | Telemedicine | Digital health | Residents |  |
| 108 | Montalto et al., 2015  (126) | Australia | Conference paper | Non-experimental | Telemedicine  (Mobile X-ray) | Digital health | Residents |  |
| 109 | Toh et al., 2015b  (127) | Singapore | Conference paper | Qualitative | Telemedicine | Digital health | Residents |  |
| 110 | Toh et al., 2015a  (128) | Singapore | Conference paper | Non-experimental | Telemedicine | Digital health | Residents |  |
| 111 | Volicer, 2015  (129) | USA | Journal article | Non-research article | Telemedicine | Digital health | Residents |  |
| 112 | De Luca et al., 2016  (130) | Italy | Journal article | Experimental | Telemedicine | Digital health | Residents |  |
| 113 | Dozet et al., 2016  (131) | Sweden | Journal article | Non-experimental | Telemedicine  (Mobile X-ray) | Digital health | Residents |  |
| 114 | Driessen et al., 2016  (132) | USA | Journal article | Non-experimental | Telemedicine | Digital health | Residents |  |
| 115 | Gaglio et al., 2016  (133) | France | Conference paper | Qualitative | Telemedicine | Digital health | Residents |  |
| 116 | Gillespie et al., 2016  (134) | USA | Journal article | Non-experimental | Telemedicine | Digital health | Residents |  |
| 117 | Morley, 2016  (135) | USA | Journal article | Non-research article | Telemedicine | Digital health | Residents |  |
| 118 | Schneider et al., 2016  (136) | USA | Journal article | Non-experimental | Telemedicine | Digital health | Residents |  |
| 119 | Kjelle & Lysdahl, 2017  (137) | Norway | Journal article | Non-research article | Telemedicine  (Mobile X-ray) | Digital health | Residents |  |
| 120 | Newbould et al., 2017  (138) | UK | Book | Non-experimental | Telemedicine | Digital health | Residents |  |
| 121 | Queyroux et al., 2017  (139) | France | Journal article | Non-experimental | Telemedicine | Digital health | Residents |  |
| 122 | Delmastro et al., 2018  (140) | Italy | Conference paper | Non-experimental | Telemedicine | Digital health | Residents |  |
| 123 | Kjelle et al., 2018  (141) | Norway | Journal article | Qualitative | Telemedicine  (Mobile X-ray) | Digital health | Residents |  |
| 124 | Esteves et al., 2019  (142) | Portugal | Journal article | System design | Telemedicine | Digital health | HCPs |  |
| 125 | Gentry et al., 2019  (143) | USA | Journal article | Non-research article | Telemedicine | Digital health | Residents |  |
| 126 | Ozkaynak et al., 2019  (144) | USA | Journal article | Qualitative | Telemedicine(Clinical assessment system) | Digital health | NH staffs |  |
| 127 | Shafiee Hanjani et al., 2019  (145) | Australia | Journal article | Mixed methods | Telemedicine | Digital health | Residents |  |
| 128 | Cormi et al., 2020  (146) | France | Journal article | Non-research article | Telemedicine | Digital health | Residents |  |
| 129 | Lai et al., 2020  (147) | USA | Journal article | Non-experimental | Teleophthalmology | Digital health | Residents |  |
| 130 | Low et al., 2020  (148) | Singapore | Journal article | Non-experimental | Telemedicine | Digital health | Residents |  |
| 131 | Ohligs et al., 2020  (149) | Germany | Journal article | Non-experimental | Telemedicine | Digital health | Residents |  |
| 132 | Alexander et al., 2021  (150) | USA | Journal article | Non-experimental | Telemedicine | Digital health | Residents |  |
| 133 | Okamoto et al., 2021  (151) | USA | Conference paper | Non research article | Telemedicine | Digital health | Residents |  |
| 134 | Lenderink & Egberts, 2004  (152) | Netherlands | Journal article | Non-experimental | Information management and decision making | IMS^g^ | Nurses | Information management and decision making |
| 135 | Alexander, 2005  (153) | USA | Thesis | Non-experimental | Information management and decision making | IMS | Administrative staffs |  |
| 136 | Byrne, 2005  (154) | USA | Thesis | Experimental | Information management and decision making | IMS | NH staffs |  |
| 137 | Celler et al., 2006  (155) | Australia | Conference paper | Non-experimental | Information management and decision making | IMS | NH staffs |  |
| 138 | Cherry, 2006  (156) | USA | Thesis | Qualitative | Information management and decision making | IMS | HCPs |  |
| 139 | Alexander et al., 2007  (157) | USA | Journal article | Qualitative | Information management and decision making | IMS | NH staffs |  |
| 140 | Alexander, 2008  (158) | USA | Journal article | Non-experimental | Information management and decision making | IMS | NH staffs |  |
| 141 | Breen & Zhang, 2008  (159) | USA | Journal article | Non-research article | Information management and decision making | IMS | Nurses and other medical practitioners |  |
| 142 | Yu et al., 2008  (160) | China | Journal article | Mixed methods | Information management and decision making | IMS | Caregivers |  |
| 143 | Sax & Lawrence, 2009  (161) | Australia | Conference paper | System design | Information management and decision making | IMS | Nurses |  |
| 144 | Scott-Cawiezell et al., 2009  (162) | USA | Journal article | Non-experimental | Information management and decision making | IMS | Practitioners, nursing staffs, medication administrators and NH leadership |  |
| 145 | Ohol, 2010  (163) | USA | Thesis | System design | Information management and decision making | IMS | Clinical staffs |  |
| 146 | Matusitz et al., 2013  (4) | USA | Journal article | Non-research article | Information management and decision making | IMS | Healthcare practitioners |  |
| 147 | Alexander et al., 2015  (164) | USA | Journal article | Qualitative | Information management and decision making | IMS | Clinical staffs |  |
| 148 | Huang et al., 2015  (165) | China | Journal article | System design | Information management and decision making | IMS | NH staffs and administration |  |
| 149 | Wang, 2016  (166) | China | Journal article | Non-research article | Information management and decision making | IMS | HCPs and administration |  |
| 150 | Zhang, 2017  (167) | China | Thesis | System design | Information management and decision making | IMS | Doctors, nurses and caregivers |  |
| 151 | Xie, 2016  (168) | China | Thesis | System design | Information management and decision making | IMS | Caregivers |  |
| 152 | Ausserhofer et al., 2021  (169) | Switzerland | Journal article | Non-experimental | Information management and decision making | IMS | Care workers and nurses |  |
| 153 | Kei Hong et al., 2021  (170) | China | Journal article | Non-experimental | Information management and decision making | IMS | HCPs |  |
| 154 | Masuda & Numao, 2017  (171) | Japan | Conference paper | System design | Clinical data anaylsis (Diagnosis) | IoT | Residents | Clinical data analysis by AI |
| 155 | Roh & Park, 2017  (9) | South Korea | Journal article | System design | Quality of Life measurements | Big data and AI | HCPs |  |
| 156 | González et al., 2019  (172) | Spain | Journal article | System design | Clinical data anaylsis (frailty and cognition status) | IoT | HCPs |  |
| 157 | Kokubo & Kamiya, 2019  (173) | USA | Conference paper | Non-experimental | A new signal parameter estimation algorithm for vital signs monitoring | Big data and AI | HCPs |  |
| 158 | Ambagtsheer et al., 2020  (174) | Australia | Journal article | Non-experimental | Identifying frailty by using artificial intelligence (AI) algorithms | Big data and AI | HCPs |  |
| 159 | Hsu et al., 2010  (175) | Taiwan, China | Journal article | System design | ADLs assistance (Pillbox) | IoT | Residents | Activities of daily living (ADLs^h^) assistance |
| 160 | Chang et al., 2011  (176) | Taiwan, China | Journal article | System design | ADLs assistance (Pillbox) | IoT | Residents |  |
| 161 | Sun et al., 2015  (7) | China | Journal article | System design | ADLs assistance (Intelligent robot) | Computing technologies | Residents |  |
| 162 | Tsai et al., 2017  (177) | Taiwan, China | Conference paper | System design | ADLs assistance (Pillbox) | IoT | Residents |  |

^a^ Residents=Nursing home residents

^b^ NH=Nursing home

^c^ Experimental study: The intervention or implementation of smart technologies with one or more control variables of the research subjects conducted in nursing home setting to measure or compare the effect of this manipulation on the users or medical outcomes

^d^ Non-research article: Non-original research articles such as review, perspective, controversies, and editorial

^e^ HCPs=Healthcare professionals

^f^ Non-experimental study: No control, manipulate or prediction of intervention and implementation of smart technologies, and the conclusion came through the interpretation, observation or interactions

^g^ IMS=Information management system

^h^ ADLs=Activities of daily living

1. Betgé-Brezetz S, Dupont MP, Ghorbel M, Kamga GB, Piekarec S, editors. Adaptive notification framework for smart nursing home. Proceedings of the 31st Annual International Conference of the IEEE Engineering in Medicine and Biology Society: Engineering the Future of Biomedicine, EMBC 2009; 2009.

2. Chen D, Li W. 可用于智能养老院系统终端的电路设计. 电子器件, Chinese Journal of Electron Devices. 2012;35(3):357-60.

3. Morley JE. High Technology Coming to a Nursing Home Near You. Journal of the American Medical Directors Association. 2012;13(5):409-12.

4. Matusitz J, Breen GM, Wan TT. The use of eHealth services in US nursing homes as an improvement of healthcare delivery to residents. Aging Health. 2013;9(1):25-33.

5. MCA. 民政部发布《智能养老物联网应用示范工程》: 社会福利促进司; 2014 [Available from: <http://news.21csp.com.cn/c3/201406/72132.html>.

6. Wang J. 基于数据融合的分布式智能养老系统的应用研究 [硕士]: 沈阳大学; 2014.

7. Sun Y, Wang Y, Ai H. 基于助老服务机器人的智慧养老院系统研究. 科技视界, Science&Technology Vision. 2015(16):27,96.

8. Siciliano B, Khatib O. Springer handbook of robotics: Springer; 2016.

9. Roh EH, Park SC. A study on the quality of life improvement in fixed IoT environments: Utilizing active aging biomarkers and big data. Quality Innovation Prosperity. 2017;21(2):52-70.

10. Xie J. 智能养老院室内定位算法及应用研究 [硕士]: 中国科学院大学; 2017.

11. Baidu. 智慧养老云服务平台 2018 [Available from: <https://wenku.baidu.com/view/d100cd6c4a73f242336c1eb91a37f111f1850d93.html?re=view>.

12. BOE Technology Group Co. L. INTERIM REPORT 2018 2018 [Available from: <http://file.finance.sina.com.cn/211.154.219.97:9494/MRGG/CNSESZ_STOCK/2018/2018-8/2018-08-28/4700772.PDF>.

13. Gamberini L, Fabbri L, Orso V, Pluchino P, Ruggiero R, Barattini R, et al. A cyber secured IoT: Fostering smart living and safety of fragile individuals in intelligent environments. Lecture Notes in Electrical Engineering. 5442018. p. 335-42.

14. Lee S, Shin I, Lee N, editors. Development of IoT based Smart Signage Platform. 2018 International Conference on Information and Communication Technology Convergence (ICTC); 2018 17-19 Oct. 2018.

15. Ce.cn. 物联网+大数据+智慧养老 让无锡老年生活更安心子女更省心: 中国经济网; 2019 [Available from: <https://www.yanglaoditu.com/news/industry/11217.html>.

16. Deng S. 智慧养老院人员定位系统研制 [硕士]: 电子科技大学; 2019.

17. Huang Y. 基于深度学习的智能养老院管控系统关键技术研究 [硕士]: 中北大学; 2019.

18. Huang P, Lin C, Wang Y, Hsieh H, editors. Development of Health Care System Based on Wearable Devices. 2019 Prognostics and System Health Management Conference (PHM-Paris); 2019 2-5 May 2019.

19. Mahieu C, Ongenae F, De Backere F, Bonte P, De Turck F, Simoens P. Semantics-based platform for context-aware and personalized robot interaction in the internet of robotic things. Journal of Systems and Software. 2019;149:138-57.

20. Tang V, Choy KL, Ho GTS, Lam HY, Tsang YP. An IoMT-based geriatric care management system for achieving smart health in nursing homes. Industrial Management and Data Systems. 2019;119(8):1819-40.

21. Xiexiebang. 中国信息产业《智能养老院系统解决方案》 2019 [Available from: <https://www.xiexiebang.com/a5/2019051319/96e56884a89fba1e.html>.

22. Xu L, Tuo L. 广州市智慧养老院失能老人管理系统分析研究. 新商务周刊, New Business Weekly. 2019(10):86-7.

23. Cui F, Ma L, Hou G, Pang Z, Hou Y, Li L. Development of smart nursing homes using systems engineering methodologies in industry 4.0. Enterprise Information Systems. 2020;14(4):463-79.

24. Shenghuo. 广州好的智能养老院的位置: 生活服务网; 2020 [Available from: <http://shenghuo.huangye88.com/xinxi/0529co6e02888a.html>.

25. Wang P. 基于嵌入式的智能养老院监控系统 [硕士]: 长春工程学院; 2020.

26. Korte. Delivering World-Class Care and Quality of Life-The Owner's Guide to Senior Liver Design and Construction [Available from: <https://www.korteco.com/sites/default/files/KOR-senior-living-v8.pdf>.

27. Liuye. 智能养老院管理系统解决方案: Liuye Science and Technology, Ltd; [Available from: <http://www.liuyesoft.com.cn/lys/NewsInfo.asp?id=802>.

28. MHURD. 养老设施智能化系统技术标准(征求意见稿): 中华人民共和国住房和城乡建设部; [Available from: <http://www.mohurd.gov.cn/zqyj/201809/W020180921031507.pdf>.

29. SheCuiTong. 金中智慧养老平台 [Available from: <http://www.kingonsoft.com:81/ylxx/zzfw/index.jhtml>.

30. Telpo. Smart Nursing Home Intelligent Terminal [Available from: <http://www.telpouc.com/Article-ndetail-id-487.html>

31. Suzuki R, Otake S, Izutsu T, Yoshida M, Iwaya T. Monitoring daily living activities of elderly people in a nursing home using an infrared motion-detection system. Telemedicine Journal and e-Health. 2006;12(2):146-55.

32. Fischer M, Lim YY, Lawrence E, Ganguli LK, editors. ReMoteCare: Health Monitoring with Streaming Video. 2008 7th International Conference on Mobile Business; 2008 7-8 July 2008.

33. Lin YJ, Su MJ, Chen HS, Lin CI, editors. A study of integrating digital health network with UPnP in an elderly nursing home. 13th IEEE Asia-Pacific Computer Systems Architecture Conference, ACSAC 2008; 2008.

34. Biswas J, Jayachandran M, Shue L, Gopalakrishnan K, Yap P. Design and trial deployment of a practical sleep activity pattern monitoring system. Lecture Notes in Computer Science (including subseries Lecture Notes in Artificial Intelligence and Lecture Notes in Bioinformatics)2009. p. 190-200.

35. Hu F, Xiao Y, Hao Q. Congestion-aware, loss-resilient bio-monitoring sensor networking for mobile health applications. IEEE Journal on Selected Areas in Communications. 2009;27(4):450-65.

36. Fraile JA, Bajo J, Corchado JM, Abraham A. Applying wearable solutions in dependent environments. IEEE Transactions on Information Technology in Biomedicine. 2010;14(6):1459-67.

37. Pallikonda Rajasekaran M, Radhakrishnan S, Subbaraj P. Sensor grid applications in patient monitoring. Future Generation Computer Systems. 2010;26(4):569-75.

38. Gower V, Andrich R, Braghieri P, Susi A. An advanced monitoring system for residential care facilities. Assistive Technology Research Series. 292011. p. 57-64.

39. Lee S, Kim J, Lee M. The design of the m-health service application using a Nintendo DS game console. Telemedicine journal and e-health : the official journal of the American Telemedicine Association. 2011;17(2):124-30.

40. Sun Y. Human daily activity detect system optimization method using Bayesian network based on wireless sensor network. Advances in Intelligent and Soft Computing2011. p. 721-5.

41. Wu M, Huang W, editors. Health care platform with safety monitoring for long-term care institutions. The 7th International Conference on Networked Computing and Advanced Information Management; 2011 21-23 June 2011.

42. Back I, Kallio J, Perala S, Makela K. Remote monitoring of nursing home residents using a humanoid robot. Journal of telemedicine and telecare. 2012;18(6):357-61.

43. Chang YJ, Chen CH, Lin LF, Han RP, Huang WT, Lee GC. Wireless sensor networks for vital signs monitoring: Application in a nursing home. International Journal of Distributed Sensor Networks. 2012;2012.

44. Nijhof N, Van Gemert-Pijnen JEWC, De Jong GEN, Ankoné JW, Seydel ER. How assistive technology can support dementia care: A study about the effects of the IST Vivago watch on patients' sleeping behavior and the care delivery process in a nursing home. Technology and Disability. 2012;24(2):103-15.

45. Ghorbel M, Betgé-Brezetz S, Dupont MP, Kamga GB, Piekarec S, Reerink J, et al. Multimodal notification framework for elderly and professional in a smart nursing home. Journal on Multimodal User Interfaces. 2013;7(4):281-97.

46. Huang J, Wang T, Su T, Lan K, editors. Design and deployment of a heart rate monitoring system in a senior center. 2013 IEEE International Conference on Sensing, Communications and Networking (SECON); 2013 24-27 June 2013.

47. Matsui T, Yoshida Y, Kagawa M, Kubota M, Kurita A. Development of a practicable non-contact bedside autonomic activation monitoring system using microwave radars and its clinical application in elderly people. Journal of clinical monitoring and computing. 2013;27(3):351-6.

48. Neuhaeuser J, D'Angelo LT. Collecting and distributing wearable sensor data: an embedded personal area network to local area network gateway server. Conference proceedings : Annual International Conference of the IEEE Engineering in Medicine and Biology Society IEEE Engineering in Medicine and Biology Society Annual Conference. 2013;2013:4650-3.

49. Pan Y. 基于物联网技术的养老院管理系统设计与实现 [硕士]: 杭州电子科技大学; 2013.

50. Tseng KC, Hsu CL, Chuang YH. Designing an intelligent health monitoring system and exploring user acceptance for the elderly. Journal of medical systems. 2013;37(6):9967.

51. Abbate S, Avvenuti M, Light J. Usability study of a wireless monitoring system among Alzheimer’s disease elderly population. International journal of telemedicine and applications. 2014;2014.

52. Chu J, Li J, Zhu C, Yin H, Liu Z. 基于无线传感网的智能养老院监护系统终端电路设计. 电子制作, Practical Electronics. 2014(21):48-.

53. Liu YW, Hsu YL. Developing a bed-centered nursing home care management system. Gerontechnology. 2014;13(2):108-9.

54. Zhu X, Zhou X, Chen W, Kitamura K, Nemoto T, editors. Estimation of Sleep Quality of Residents in Nursing Homes Using an Internet-Based Automatic Monitoring System. 2014 IEEE 11th Intl Conf on Ubiquitous Intelligence and Computing and 2014 IEEE 11th Intl Conf on Autonomic and Trusted Computing and 2014 IEEE 14th Intl Conf on Scalable Computing and Communications and Its Associated Workshops; 2014 9-12 Dec. 2014.

55. Andò B, Baglio S, Lombardo CO, Marletta V, editors. A multi-user assistive system for the user safety monitoring in care facilities. 2015 IEEE International Workshop on Measurements & Networking (M&N); 2015 12-13 Oct. 2015.

56. Carvalho CMA, Rodrigues CAP, Aguilar PAC, De Castro MF, Andrade RMC, Boudy J, et al., editors. Adaptive tracking model in the framework of medical nursing home using infrared sensors. 2015 IEEE Globecom Workshops, GC Wkshps 2015 - Proceedings; 2015.

57. Yu X, Weller P, Grattan KTV. A WSN healthcare monitoring system for elderly people in geriatric facilities. Studies in health technology and informatics2015. p. 567-71.

58. Danielsen A. Non-intrusive bedside event recognition using infrared array and ultrasonic sensor. Lecture Notes in Computer Science (including subseries Lecture Notes in Artificial Intelligence and Lecture Notes in Bioinformatics)2016. p. 15-25.

59. Dias PVGF, Costa EDM, Tcheou MP, Lovisolo L, editors. Fall detection monitoring system with position detection for elderly at indoor environments under supervision. 2016 8th IEEE Latin-American Conference on Communications, LATINCOM 2016; 2016.

60. Lopez-Samaniego L, Garcia-Zapirain B. A Robot-Based Tool for Physical and Cognitive Rehabilitation of Elderly People Using Biofeedback. International journal of environmental research and public health. 2016;13(12).

61. Ansefine KE, Muzakki, Sanudin, Anggadjaja E, Santoso H, editors. Smart and wearable technology approach for elderly monitoring in nursing home. 2017 IEEE 3rd International Conference on Engineering Technologies and Social Sciences (ICETSS); 2017 7-8 Aug. 2017.

62. Jiang H. 基于GPS/GSM养老院监护系统 [硕士]: 牡丹江师范学院; 2017.

63. Mendes S, Queiroz J, Leitao P, editors. Data driven multi-agent m-health system to characterize the daily activities of elderly people. Iberian Conference on Information Systems and Technologies, CISTI; 2017.

64. Mendoza MB, Bergado CA, De Castro JLB, Siasat RGT, editors. Tracking system for patients with Alzheimer's disease in a nursing home. IEEE Region 10 Annual International Conference, Proceedings/TENCON; 2017.

65. Montanini L, Raffaeli L, de Santis A, del Campo A, Chiatti C, Paciello L, et al. Supporting caregivers in nursing homes for Alzheimer’s disease patients: A technological approach to overnight supervision. Communications in Computer and Information Science2017. p. 1-19.

66. Saod AHM, Ghani SJAM, Harron NA, Ramlan SA, Rashid ANA, Ishak NH, editors. Android-based elderly support system. 2017 IEEE Symposium on Computer Applications & Industrial Electronics (ISCAIE); 2017 24-25 April 2017.

67. Singh D, Kropf J, Hanke S, Holzinger A. Ambient assisted living technologies from the perspectives of older people and professionals. Lecture Notes in Computer Science (including subseries Lecture Notes in Artificial Intelligence and Lecture Notes in Bioinformatics)2017. p. 255-66.

68. Wu Y, Liu L, Kang J, Li L, Huang B. Measuring the wellness indices of the elderly using RFID sensors data in a smart nursing home. Lecture Notes in Computer Science (including subseries Lecture Notes in Artificial Intelligence and Lecture Notes in Bioinformatics)2017. p. 66-73.

69. Bleda AL, Maestre R, Beteta MA, Vidal JA, editors. AmICare: Ambient Intelligent and Assistive System for Caregivers Support. Proceedings - 16th International Conference on Embedded and Ubiquitous Computing, EUC 2018; 2018.

70. Donnelly S, Reginatto B, Kearns O, Mc Carthy M, Byrom B, Muehlhausen W, et al. The Burden of a Remote Trial in a Nursing Home Setting: Qualitative Study. Journal of medical Internet research. 2018;20(6):e220.

71. Mahfuz S, Isah H, Zulkernine F, Nicholls P, editors. Detecting Irregular Patterns in IoT Streaming Data for Fall Detection. 2018 IEEE 9th Annual Information Technology, Electronics and Mobile Communication Conference (IEMCON); 2018 1-3 Nov. 2018.

72. Morita T, Taki K, Fujimoto M, Suwa H, Arakawa Y, Yasumoto K, editors. BLE Beacon-based Activity Monitoring System toward Automatic Generation of Daily Report. 2018 IEEE International Conference on Pervasive Computing and Communications Workshops, PerCom Workshops 2018; 2018.

73. Wu Y, Liu L, Li L, Lu M, Li L. Determining senior wellness status using an intelligent system based on wireless sensor network and bioinformation. Web Intelligence. 2018;16(3):159-66.

74. Borelli E, Paolini G, Antoniazzi F, Barbiroli M, Benassi F, Chesani F, et al. HABITAT: An IoT solution for independent elderly. Sensors (Switzerland). 2019;19(5).

75. Cai C, Wang P. 老人跌倒姿态检测研究 %J 长春工程学院学报(自然科学版). 2019;20(04):29-34.

76. Delmastro F, Dolciotti C, La Rosa D, Di Martino F, Magrini M, Coscetti S, et al. Experimenting mobile and e-health services with frail MCI older people. Information (Switzerland). 2019;10(8).

77. Fong ACM, Fong B, Hong G, editors. Short-range tracking using smart clothing sensors : AA case study of using low power wireless sensors for pateints tracking in a nursing home setting. 2018 IEEE 3rd International Conference on Communication and Information Systems, ICCIS 2018; 2019.

78. Ghosh N, Maity S, Maity K, Saha S, editors. Non-Parametric Learning Technique for Activity Recognition in Elderly Patients. TENCON 2019 - 2019 IEEE Region 10 Conference (TENCON); 2019 17-20 Oct. 2019.

79. Lenoir J. Effective User Interface of IoT System at Nursing Homes. Communications in Computer and Information Science2019. p. 490-8.

80. Shen M. 基于物联网技术的养老院管理系统 计算机产品与流通. 2019(08):122.

81. Takahashi K, Kitamura K, Nishida Y, Mizoguchi H, editors. Battery-less shoe-type wearable location sensor system for monitoring people with dementia. Proceedings of the International Conference on Sensing Technology, ICST; 2019.

82. Toda K, Shinomiya N, editors. Machine learning-based fall detection system for the elderly using passive RFID sensor tags. Proceedings of the International Conference on Sensing Technology, ICST; 2019.

83. Xiao B. 基于智能床垫养老监护数据的分析与应用 [硕士]: 东华大学; 2019.

84. Yoo B, Muralidharan S, Lee C, Lee J, Ko H, editors. KLog-Home: A holistic approach of in-situ monitoring in elderly-care home. Proceedings - 22nd IEEE International Conference on Computational Science and Engineering and 17th IEEE International Conference on Embedded and Ubiquitous Computing, CSE/EUC 2019; 2019.

85. Buisseret F, Catinus L, Grenard R, Jojczyk L, Fievez D, Barvaux V, et al. Timed Up and Go and Six-Minute Walking Tests with Wearable Inertial Sensor: One Step Further for the Prediction of the Risk of Fall in Elderly Nursing Home People. Sensors (Basel, Switzerland). 2020;20(11).

86. Chen W, Wang X, Chen J, Ding Z, Li J, Li B. An Alarm System Based on BP Neural Network Algorithm for the Detection of Falls to Elderly Person. Lecture Notes of the Institute for Computer Sciences, Social-Informatics and Telecommunications Engineering, LNICST2021. p. 571-81.

87. Gharti P, editor A study of fall detection monitoring system for elderly people through IOT and mobile based application devices in indoor environment. 2020 5th International Conference on Innovative Technologies in Intelligent Systems and Industrial Applications (CITISIA); 2020 25-27 Nov. 2020.

88. Lanza F, Seidita V, Chella A. Agents and robots for collaborating and supporting physicians in healthcare scenarios. J Biomed Inform. 2020;108:103483.

89. Lee SK, Ahn J, Shin JH, Lee JY. Application of Machine Learning Methods in Nursing Home Research. International journal of environmental research and public health. 2020;17(17).

90. Mishkhal I, Sarah Abd ALK, Hassan Hadi S, Alqayyar A. Deep Learning with network of Wearable sensors for preventing the Risk of Falls for Older People. IOP Conference Series Materials Science and Engineering. 2020;928(3).

91. Suzuki M, Yamamoto R, Ishiguro Y, Sasaki H, Kotaki H. Deep learning prediction of falls among nursing home residents with Alzheimer's disease. Geriatrics & gerontology international. 2020;20(6):589-94.

92. Wan HC, Chin KS. Exploring internet of healthcare things for establishing an integrated care link system in the healthcare industry. International Journal of Engineering Business Management. 2021;13.

93. Chen IH, Chen CH, Ting YC, Hung WL, Cheng BY. The Guardian Slippers: Designing an IoT Device to Enhance Safety for the Elderly in the Nursing Home. Lecture Notes in Networks and Systems2021. p. 369-77.

94. Flores-Martin D, Rojo J, Moguel E, Berrocal J, Murillo JM. Smart Nursing Homes: Self-Management Architecture Based on IoT and Machine Learning for Rural Areas. Wireless Communications and Mobile Computing. 2021;2021.

95. Chan WM, Woo J, Hui E, Hjelm NM. The role of telenursing in the provision of geriatric outreach services to residential homes in Hong Kong. Journal of telemedicine and telecare. 2001;7(1):38-46.

96. Pallawala PMDS, Lun KC. EMR-based TeleGeriatric system. Studies in health technology and informatics2001. p. 849-53.

97. Weiner M, Schadow G, Lindbergh D, Warvel J, Abernathy G, Dexter P, et al. Secure Internet video conferencing for assessing acute medical problems in a nursing facility. Proceedings AMIA Symposium. 2001:751-5.

98. Hui E, Woo J. Telehealth for older patients: the Hong Kong experience. Journal of telemedicine and telecare. 2002;8 Suppl 3:S3:39-41.

99. Savenstedt S, Bucht G, Norberg L, Sandman PO. Nurse-doctor interaction in teleconsultations between a hospital and a geriatric nursing home. Journal of telemedicine and telecare. 2002;8(1):11-8.

100. Weiner M, Schadow G, Lindbergh D, Warvel J, Abernathy G, Perkins SM, et al. Clinicians' and patients' experiences and satisfaction with unscheduled, nighttime, Internet-based video conferencing for assessing acute medical problems in a nursing facility. AMIA Annual Symposium proceedings AMIA Symposium. 2003:709-13.

101. Zelickson BD. Teledermatology in the nursing home. Current problems in dermatology. 2003;32:167-71.

102. Armer JM, Harris K, Dusold JM. Application of the Concerns-Based Adoption Model to the installation of telemedicine in a rural Missouri nursing home. Journal for nurses in staff development : JNSD : official journal of the National Nursing Staff Development Organization. 2004;20(1):42-9.

103. Savenstedt S, Zingmark K, Sandman PO. Being present in a distant room: aspects of teleconsultations with older people in a nursing home. Qualitative health research. 2004;14(8):1046-57.

104. Daly JM, Jogerst G, Park JY, Kang YD, Bae T. A nursing home telehealth system: keeping residents connected. Journal of gerontological nursing. 2005;31(8):46-51.

105. Lavanya J, Goh KW, Leow YH, Chio MT, Prabaharan K, Kim E, et al. Distributed personal health information management system for dermatology at the homes for senior citizens. Conference proceedings : Annual International Conference of the IEEE Engineering in Medicine and Biology Society IEEE Engineering in Medicine and Biology Society Annual Conference. 2006;2006:6312-5.

106. Loeb MB, Carusone SB, Marrie TJ, Brazil K, Krueger P, Lohfeld L, et al. Interobserver reliability of radiologists' interpretations of mobile chest radiographs for nursing home-acquired pneumonia. Journal of the American Medical Directors Association. 2006;7(7):416-9.

107. Shulman B, Conn DK, Elford R. Geriatric telepsychiatry and telemedicine: A literature review. Canadian Journal of Geriatrics. 2006;9(4):139-46.

108. Cusack CM, Pan E, Hook JM, Vincent A, Kaelber DC, Middleton B. The value proposition in the widespread use of telehealth. Journal of telemedicine and telecare. 2008;14(4):167-8.

109. Janardhanan L, Leow YH, Chio MT, Kim Y, Soh CB. Experience with the implementation of a web-based teledermatology system in a nursing home in Singapore. Journal of telemedicine and telecare. 2008;14(8):404-9.

110. Biglan KM, Voss TS, Deuel LM, Miller D, Eason S, Fagnano M, et al. Telemedicine for the care of nursing home residents with Parkinson's disease. Movement disorders : official journal of the Movement Disorder Society. 2009;24(7):1073-6.

111. Chang JY, Chen LK, Chang CC. Perspectives and expectations for telemedicine opportunities from families of nursing home residents and caregivers in nursing homes. International journal of medical informatics. 2009;78(7):494-502.

112. Qadri S, Wang J, Ruiz J, Roos B. Personal Digital Assistants as Point-of-Care Tools in Long-Term Care Facilities: A Pilot Study. Educational Gerontology. 2009;35(4):294-307.

113. Chang HL, Shaw MJ, Lai F, Ko WJ, Ho YL, Chen HS, et al. U-Health: An example of a high-quality individualized healthcare service. Personalized Medicine. 2010;7(6):677-87.

114. Rabinowitz T, Murphy KM, Amour JL, Ricci MA, Caputo MP, Newhouse PA. Benefits of a telepsychiatry consultation service for rural nursing home residents. Telemedicine journal and e-health : the official journal of the American Telemedicine Association. 2010;16(1):34-40.

115. Wälivaara BM, Andersson S, Axelsson K. General practitioners' reasoning about using mobile distance-spanning technology in home care and in nursing home care. Scandinavian journal of caring sciences. 2011;25(1):117-25.

116. Eklund K, Klefsgard R, Ivarsson B, Geijer M. Positive experience of a mobile radiography service in nursing homes. Gerontology. 2012;58(2):107-11.

117. Gray LC, Edirippulige S, Smith AC, Beattie E, Theodoros D, Russell T, et al. Telehealth for nursing homes: The utilization of specialist services for residential care. Journal of telemedicine and telecare. 2012;18(3):142-6.

118. Handler SM, Boyce RD, Ligons FM, Perera S, Nace DA, Hochheiser H. Use and perceived benefits of mobile devices by physicians in preventing adverse drug events in the nursing home. Journal of the American Medical Directors Association. 2013;14(12):906-10.

119. Novak L, Walker S, Fonda S, Schmidt V, Vigersky R. The impact of a video phone reminder system on glycemic control in older adults with type 2 diabetes mellitus (T2DM) in a retirement home. Diabetes. 2013;62:A217.

120. Vowden K, Vowden P. A pilot study on the potential of remote support to enhance wound care for nursing-home patients. Journal of wound care. 2013;22(9):481-8.

121. Catic AG, Mattison ML, Bakaev I, Morgan M, Monti SM, Lipsitz L. ECHO-AGE: an innovative model of geriatric care for long-term care residents with dementia and behavioral issues. Journal of the American Medical Directors Association. 2014;15(12):938-42.

122. Grabowski DC, O'Malley AJ. Use of telemedicine can reduce hospitalizations of nursing home residents and generate savings for medicare. Health affairs (Project Hope). 2014;33(2):244-50.

123. Crotty M, Killington M, van den Berg M, Morris C, Taylor A, Carati C. Telerehabilitation for older people using off-the-shelf applications: acceptability and feasibility. Journal of telemedicine and telecare. 2014;20(7):370-6.

124. Doumbouya MB, Kamsu-Foguem B, Kenfack H, Foguem C. Combining conceptual graphs and argumentation for aiding in the teleexpertise. Computers in Biology and Medicine. 2015;63:157-68.

125. Huang F, Chang P, Hou IC, Tu MH, Lan CF. Use of a mobile device by nursing home residents for long-term care comprehensive geriatric self-assessment: A feasibility study. CIN - Computers Informatics Nursing. 2015;33(1):28-36.

126. Montalto M, Shay S, Le A. Evaluation of a mobile X-ray service for elderly residents of residential aged care facilities. Australian Health Review. 2015;39(5):517-21.

127. Toh HJ, Chia J, Koh E, Lam K, Magpantay GC, De Leon CM, et al., editors. User perceptions of the telemedicine programme in nursing homes the Singapore perspective. ICT4AgeingWell 2015 - Proceedings of the 1st International Conference on Information and Communication Technologies for Ageing Well and e-Health; 2015.

128. Toh HJ, Chia J, Koh E, Lam K, Magpantay GC, De Leon CM, et al. Increased engagement in telegeriatrics reduces unnecessary hospital admissions of nursing home residents. Communications in Computer and Information Science. 2015;578:81-90.

129. Volicer L. Nursing home telepsychiatry. Journal of the American Medical Directors Association. 2015;16(1):7-8.

130. De Luca R, Bramanti A, De Cola MC, Trifiletti A, Tomasello P, Torrisi M, et al. Tele-health-care in the elderly living in nursing home: the first Sicilian multimodal approach. Aging clinical and experimental research. 2016;28(4):753-9.

131. Dozet A, Ivarsson B, Eklund K, Klefsgård R, Geijer M. Radiography on wheels arrives to nursing homes – an economic assessment of a new health care technology in southern Sweden. Journal of evaluation in clinical practice. 2016;22(6):990-7.

132. Driessen J, Bonhomme A, Chang W, Nace DA, Kavalieratos D, Perera S, et al. Nursing Home Provider Perceptions of Telemedicine for Reducing Potentially Avoidable Hospitalizations. Journal of the American Medical Directors Association. 2016;17(6):519-24.

133. Gaglio G, Lewkowicz M, Tixier M, editors. "It is not because you have tools that you must use them" The difficult domestication of a telemedicine toolkit to manage emergencies in nursing homes. Proceedings of the International ACM SIGGROUP Conference on Supporting Group Work; 2016.

134. Gillespie SM, Shah MN, Wasserman EB, Wood NE, Wang H, Noyes K, et al. Reducing emergency department utilization through engagement in telemedicine by senior living communities. Telemedicine and e-Health. 2016;22(6):489-96.

135. Morley JE. Telemedicine: Coming to Nursing Homes in the Near Future. Journal of the American Medical Directors Association. 2016;17(1):1-3.

136. Schneider R, Dorsey ER, Biglan K. Telemedicine Care for Nursing Home Residents with Parkinsonism. Journal of the American Geriatrics Society. 2016;64(1):218-20.

137. Kjelle E, Lysdahl KB. Mobile radiography services in nursing homes: a systematic review of residents' and societal outcomes. BMC health services research. 2017;17(1):231.

138. Newbould L, Mountain G, Hawley M, Ariss S. Remote Health Care Provision in Care Homes. Studies in health technology and informatics. 2017;242:148-51.

139. Queyroux A, Saricassapian B, Herzog D, Muller K, Herafa I, Ducoux D, et al. Accuracy of Teledentistry for Diagnosing Dental Pathology Using Direct Examination as a Gold Standard: Results of the Tel-e-dent Study of Older Adults Living in Nursing Homes. Journal of the American Medical Directors Association. 2017;18(6):528-32.

140. Delmastro F, Dolciotti C, Palumbo F, Magrini M, Di Martino F, La Rosa D, et al., editors. Long-term care: How to improve the quality of life with mobile and e-health services. International Conference on Wireless and Mobile Computing, Networking and Communications; 2018.

141. Kjelle E, Lysdahl KB, Olerud HM, Myklebust AM. Managers' experience of success criteria and barriers to implementing mobile radiography services in nursing homes in Norway: a qualitative study. BMC health services research. 2018;18(1):301.

142. Esteves M, Esteves M, Abelha A, Machado J, editors. A mobile health application to assist health professionals: A case study in a Portuguese nursing home. ICT4AWE 2019 - Proceedings of the 5th International Conference on Information and Communication Technologies for Ageing Well and e-Health; 2019.

143. Gentry MT, Lapid MI, Rummans TA. Geriatric Telepsychiatry: Systematic Review and Policy Considerations. The American journal of geriatric psychiatry : official journal of the American Association for Geriatric Psychiatry. 2019;27(2):109-27.

144. Ozkaynak M, Reeder B, Drake C, Ferrarone P, Trautner B, Wald H, et al. Characterizing Workflow to Inform Clinical Decision Support Systems in Nursing Homes. The Gerontologist. 2019;59(6):1024-33.

145. Shafiee Hanjani L, Peel NM, Freeman CR, Gray LC. Using telehealth to enable collaboration of pharmacists and geriatricians in residential medication management reviews. International journal of clinical pharmacy. 2019;41(5):1256-61.

146. Cormi C, Chrusciel J, Laplanche D, Dramé M, Sanchez S. Telemedicine in nursing homes during the COVID-19 outbreak: A star is born (again). Geriatrics and Gerontology International. 2020;20(6):646-7.

147. Lai KY, Pathipati MP, Blumenkranz MS, Leung LS, Moshfeghi DM, Toy BC, et al. Assessment of Eye Disease and Visual Impairment in the Nursing Home Population Using Mobile Health Technology. Ophthalmic Surg Lasers Imaging Retina. 2020;51(5):262-70.

148. Low JA, Toh HJ, Tan LLC, Chia JWK, Soek ATS. The Nuts and Bolts of Utilizing Telemedicine in Nursing Homes - The GeriCare@North Experience. Journal of the American Medical Directors Association. 2020;21(8):1073-8.

149. Ohligs M, Stocklassa S, Rossaint R, Czaplik M, Follmann A. Employment of Telemedicine in Nursing Homes: Clinical Requirement Analysis, System Development and First Test Results. Clinical interventions in aging. 2020;15:1427-37.

150. Alexander GL, Powell KR, Deroche CB. An evaluation of telehealth expansion in U.S. nursing homes. Journal of the American Medical Informatics Association. 2021;28(2):342-8.

151. Okamoto L, Okamoto L, Uechi M, Blanchette P, von Preyss-Friedman S. Renaissance in the Nursing Home During the COVID-19 Pandemic: Telemedicine Blooms in a Time of Crisis. Journal of the American Medical Directors Association. 2021;22(3):B16.

152. Lenderink BW, Egberts TC. Closing the loop of the medication use process using electronic medication administration registration. Pharmacy world & science : PWS. 2004;26(4):185-90.

153. Alexander GL. Human factors, automation, and alerting mechanisms in nursing home electronic health records [Ph.D.]. Ann Arbor: University of Missouri - Columbia; 2005.

154. Byrne CM. Impact of prospective computerized clinical decision support information and targeted assistance on nursing home resident outcomes [Ph.D.]. Ann Arbor: State University of New York at Albany; 2005.

155. Celler BG, Basilakis J, Budge M, Lovel NH. A clinical monitoring and management system for residential aged care facilities. Conference proceedings : Annual International Conference of the IEEE Engineering in Medicine and Biology Society IEEE Engineering in Medicine and Biology Society Annual Conference. 2006;2006:3301-4.

156. Cherry BJ. Determining facilitators and barriers to adoption of electronic health records in long -term care facilities [D.N.Sc.]. Ann Arbor: The University of Tennessee Health Science Center; 2006.

157. Alexander GL, Rantz M, Flesner M, Diekemper M, Siem C. Clinical information systems in nursing homes: an evaluation of initial implementation strategies. CIN: Computers, Informatics, Nursing. 2007;25(4):189-97.

158. Alexander GL. A descriptive analysis of a nursing home clinical information system with decision support. Perspectives in health information management. 2008;5:12.

159. Breen GM, Zhang NJ. Introducing ehealth to nursing homes: theoretical analysis of improving resident care. Journal of medical systems. 2008;32(2):187-92.

160. Yu P, Hailey D, Li H. Caregivers' acceptance of electronic documentation in nursing homes. Journal of telemedicine and telecare. 2008;14(5):261-5.

161. Sax C, Lawrence E, editors. Point-of-treatment: Touchable e-nursing user interface for medical emergencies. 3rd International Conference on Mobile Ubiquitous Computing, Systems, Services, and Technologies, UBICOMM 2009; 2009.

162. Scott-Cawiezell J, Madsen RW, Pepper GA, Vogelsmeier A, Petroski G, Zellmer D. Medication safety teams' guided implementation of electronic medication administration records in five nursing homes. Joint Commission journal on quality and patient safety. 2009;35(1):29-35.

163. Ohol RR. Web Based Nursing Home Information System: Needs, Benefits, and Success in Providing Efficient Care at Long Term Care Facilities [M.S.]. Ann Arbor: University of Missouri - Columbia; 2010.

164. Alexander GL, Rantz M, Galambos C, Vogelsmeier A, Flesner M, Popejoy L, et al. Preparing Nursing Homes for the Future of Health Information Exchange. Applied clinical informatics. 2015;6(2):248-66.

165. Huang Z, Chen Z, Liu Z. 智能养老院综合管理系统设计. 电子科技, Electronic Science and Technology. 2015;28(11):132-4.

166. Wang Y. “物联网”实现养老档案智能化. 兰台世界. 2016(S2):103-4.

167. Zhang C. 面向养老院的健康管理服务设计研究 [硕士]: 江南大学; 2017.

168. Xie X. 颐老会智慧养老护理管理系统的研究与实现 [硕士]: 广东工业大学; 2016.

169. Ausserhofer D, Favez L, Simon M, Zúñiga F. Electronic Health Record Use in Swiss Nursing Homes and Its Association With Implicit Rationing of Nursing Care Documentation: Multicenter Cross-sectional Survey Study. JMIR Medical Informatics. 2021;9(3).

170. Kei Hong S, Ting CW, Chui PL, Teddy Tai-Ning L, Sau Chu C, Cheung YT. Medication Management Service for Old Age Homes in Hong Kong Using Information Technology, Automation Technology, and the Internet of Things: Pre-Post Interventional Study. JMIR Medical Informatics. 2021;9(2).

171. Masuda S, Numao M, editors. Sensor-based detection of invisible changes in activities towards visualizing disuse syndrome. AAAI Spring Symposium - Technical Report; 2017.

172. González I, Navarro FJ, Fontecha J, Cabañero-Gómez L, Hervás R. An Internet of Things infrastructure for gait characterization in assisted living environments and its application in the discovery of associations between frailty and cognition. International Journal of Distributed Sensor Networks. 2019;15(10).

173. Kokubo R, Kamiya Y, editors. A novel period estimation method for periodic signals suitable for vital sensing. ACM International Conference Proceeding Series; 2019.

174. Ambagtsheer RC, Shafiabady N, Dent E, Seiboth C, Beilby J. The application of artificial intelligence (AI) techniques to identify frailty within a residential aged care administrative data set. International journal of medical informatics. 2020;136.

175. Hsu WC, Kuo CW, Chang WW, Chang JJ, Hou YT, Lan YC, et al., editors. A WSN smart medication system. Procedia Engineering; 2010.

176. Chang WW, Sung TJ, Huang HW, Hsu WC, Kuo CW, Chang JJ, et al. A smart medication system using wireless sensor network technologies. Sensors and Actuators, A: Physical. 2011;172(1):315-21.

177. Tsai HL, Tseng CH, Wang LC, Juang FS, editors. Bidirectional smart pill box monitored through internet and receiving reminding message from remote relatives. 2017 IEEE International Conference on Consumer Electronics - Taiwan, ICCE-TW 2017; 2017.
