# Supplementary material for: Defining the concepts of a smart nursing home and its potential technology utilities that integrate medical services and are acceptable to stakeholders: a scoping review
Source: BMC Geriatr. 2022 Oct 7;22:787. doi: 10.1186/s12877-022-03424-6 (PMC9540152; doi:10.1186/s12877-022-03424-6)
Supplement: Supplementary file 4 — Additional file 4. Code Sheet for Defining the Concepts and Criteria of a Smart Nursing Home. [file 12877_2022_3424_MOESM4_ESM.docx]

**Supplementary file 4: Code Sheet for Defining the Concepts and Criteria of a Smart Nursing Home**

**A: Concept of Smart NH**^a^

| No | Authors and year | Data Extract/ Quotation | Codes | Description | Themes |
| --- | --- | --- | --- | --- | --- |
| 1 | Baidu, 2018 | Building a real-time positioning system for the elderly through wearable terminals to monitor the current location and status of the elderly in real time visualization | IoT^b^ | The concept of smartness in nursing home settings is using a new generation of information technologies such as the internet of things (loT), computing technologies, cloud computing, big data and AI^a^, information management system and digital health, to transform traditional nursing care in an all-round way, making healthcare more efficient, more effective, and more personalized. | Application of smart technologies (Smartness) |
| 2 | Ce.cn, 2019 | The management platform of a smart nursing home adopts new generation of information technology such as Internet of Things, cloud computing, big data, artificial intelligence |  |  |  |
| 3 | Chen & Li, 2012 | A system by using sensor technology, communication technology, location technology, ultra low power consumption technology for smart nursing home |  |  |  |
| 4 | Gamberini et al., 2018 | Smart buildings that will be equipped with IoT technologies to assist elderly people daily living |  |  |  |
| 5 | Huang et al., 2019 | This study proposes an IoT integration architecture with wearable devices |  |  |  |
| 6 | Korte | Smart architectural design and construction allow you to build the proper environments for seniors |  |  |  |
| 7 | Lee et al., 2018 | Each patient in the nursing home is wearing smart band that can measure heart rate and the amount of steps  The proposed platform provides IoT based connectivity between sensors and signage platform |  |  |  |
| 8 | Mahieu et al., 2019 | Building services that generate interaction tasks for social robots in smart IoT environments |  |  |  |
| 9 | MCA, 2014 | To meet the elderly people's care demand and guaranttee the legitimate rights of the elderly people as the starting point, the application of IoT technology on position monitoring, fall detection, bed-side monitoring, tracking elderly with demansia, behavior analysis, self-help medical examination, activity measurement, video intelligent linkage and other services were carried out for the elderly people in nursing homes |  |  |  |
| 10 | Roh & Park, 2017 | The main server is equipped with a Zigbee communication module so that the server itself can be a master IoT  The nursing home becomes a smart building providing a smart built environment (SBE), or a fixed IoT environment |  |  |  |
| 11 | Shenghuo, 2020 | Intelligent monitoring: Matching precise positioning under infrared sensing |  |  |  |
| 12 | Tang et al., 2019 | An IoMT-based geriatric care management system (I-GCMS) for facilitating the adoption of smart health in nursing homes |  |  |  |
| 13 | Wang, 2014 | IoT is the core of smart nursing home  Install smart devices such as devices capable of wireless positioning on the elderly |  |  |  |
| 14 | Wang, 2020 | Smart nursing home monitoring system, terminal hardware, wearable device monitoring system, environmental monitoring |  |  |  |
| 15 | Xie, 2017 | Wireless technology continues to develop and mature that providing an opportunity for smart senior care refinement services |  |  |  |
| 16 | Xiexiebang, 2019 | Smart nursing home system adopts Zigbee, sensors, cloud computing, medical IoT, mobile Internet and other advanced technologies and concepts |  |  |  |
| 17 | Xu & Tuo, 2019 | Use of various information and communication technology tools such as Internet and IoT |  |  |  |
| 18 | Cui et al., 2020 | We identify key system elements, including hardware elements like living space and devices, software elements like cloud and information systems, and human elements like inhabitants and caregivers | Computing technologies |  |  |
| 19 | Korte | Nursing stations now come equipped with the latest computing technology, and facilities are set up to work with modern medical devices and IT infrastructures |  |  |  |
| 20 | SheCuiTong | 2:167 The management platform of ‘She Cun Tong’ smart nursing homes based on a constumalized software to provide professional services for all elderly institutional care |  |  |  |
| 21 | Telpo | The voice SDK for partners to integrate with software for more functions or further development |  |  |  |
| 22 | Ce.cn, 2019 | Building a cloud integrated elderly institutional care system could implement the rescue of emergent events, testing based on bed-bound, and the wandering  Baihe Yihe Hospital is supported by the monitoring center and cloud monitoring platform | Cloud computing |  |  |
| 23 | Cui et al., 2020 | Enormous data flows may be imported into the simulation model. Machine learning algorithms can help the simulation obtain more accurate parameters and diffusion mechanism. This is exactly the advantage of the smart nursing home | Big data and AI^c^ |  |  |
| 24 | Mahieu et al., 2019 | Data-driven workflow that allows developers of interaction services |  |  |  |
| 25 | MHURD | Intelligent information integration platform/system |  |  |  |
| 26 | Telpo | Telpo intelligent voice terminal allows kinds of smart nursing home applications |  |  |  |
| 27 | Xu & Tuo, 2019 | Intelligently respond to the needs of their health |  |  |  |
| 28 | Baidu, 2018 | Establishing health information system (HIS), collecting daily health records and rehabilitation data for analysis, automatical reminder and notification  On the basis of the existing information platform, such as administration and HIS system, the service will address on real-time positioning, monitoring elderly residents and mHealth for rehabilitation or cares  Establishing a unified control platform, the administration of the hospital could improve the management and ease to control  It is an integration between the smart senior care system and medical information system | Information management system (IMS) |  |  |
| 29 | Liuye | Smart Nursing Home Management System Solution: ‘Six Industries Smart Nursing Home Management System’ is a set of software developed specifically for nursing homes |  |  |  |
| 30 | MHURD | Health management system  Nursing homes should be equipped with the following systems: Basic business office and information management system  Information access system, cabling system, mobile communication indoor signal coverage system, subscriber telephone exchange system, information network system, cable TV system, public broadcasting system, information guidance and distribution system, and other respective facilities  The construction of smart institutional care for the elderly people should adopt modern information technology, network technology and integration technology to implement services |  |  |  |
| 31 | Morley, 2012 | Electronic Medical Record includes: 1. Physician notes and ordering; 2 .Integration with MDS 3.0; 3 Integration with nurses, vital signs, skin and behavior assessments, falls, glucose levels, therapy assessments, and laboratory levels; 4. Pharmacy checks on anticholinergic burden, warfarin interactions and Geriatric Risk Assessment Med Guide and “Beers” list; 5. Integrated record for transfers to and from hospital |  |  |  |
| 32 | BOE Technology Group Co., 2018 | Integrated professional healthcare services focused on digital medical care…  The smart healthcare service sub-division covers digital hospitals, regenerative medicine, healthcare parks, healthcare centres, etc. It offers B2C customers online-and-offline integrated professional healthcare services…  Providing individual and household customers with mobile health testing and online diagnosis services including health management, online diagnosis, AI diagnosis and smart referral…  Division H includes the mobile healthcare IoT platform sub-division and the smart healthcare service | Digital health |  |  |
| 33 | MHURD | Intelligent system for nursing homes includes public service, smart card application, information security management and other information applications |  |  |  |
| 34 | Morley, 2012 | Bringing smart technology... including Telemedicine… health apps into nursing home |  |  |  |
| 35 | Shenghuo, 2020 | Using smart App, establishing a 24-hour health monitoring system to monitor the health records and living conditions of the elderly residents, allows their children who are far away from to check their partients in real time through the App |  |  |  |
| 36 | Telpo | Guangdong Telpo smart nursing home intelligent terminal is a kind of Android telephone |  |  |  |
| 37 | Shenghuo, 2020 | Intelligent devices for rehabilitation: Importing phisical practice devices from Japan which attached data management, could record the elderly daily practice and improve their active living | Assistive devices |  |  |
| 38 | Siciliano & Khatib, 2016 | Robotics and automation are beginning to find applications in the physical task associated with patient care, therapy and oversight |  |  |  |
| 39 | Sun et al., 2015 | Six application systems (including robot service system, environmental monitoring system, intelligent video analysis system, webcasting system, health management system, and elderly locating and calling system) are integrated into an intelligent support platform to support nursing home infrastructure and services  Smart nursing home is based on assistant robot for the elderly people |  |  |  |
| 40 | Cui et al., 2020 | High technology is coming to nursing homes from basic functions like the electronic medical record to complex ones like electronic monitoring | Intelligent nursing | A nursing home offers technology-assisted nursing care for the people who require a lot of assistance with activities of daily living to improve their quality of life in relation to their goals, expectations, standards and concerns. | Technology-assisted nursing care |
| 41 | Deng, 2019 | The intelligent nursing refers to using IoT, wearable technology and mobile technology to provide the elderly with real-time, efficient and intelligent services |  |  |  |
| 42 | MCA, 2014 | Based on nursing home to provide intensive and intelligent care |  |  |  |
| 43 | Tang et al., 2019 | Without the adoption of smart health, caregivers in nursing homes still rely on traditional manual approaches to check and record the biometric data of the elderly residents, and review their care plans |  |  |  |
| 44 | Korte | Automated tracking and alerts can inform nurses and care providers when patients fall, require attendance or enter or exit certain areas | Automated tracking, monitoring and alerts |  |  |
| 45 | Lee et al., 2018 | Smart nursing home service based on the proposed platform. The service monitors health status of users and detects emergency situation by using smart band |  |  |  |
| 46 | Xie, 2017 | Indoor monitoring technology has become an essential technology in elderly care services |  |  |  |
| 47 | Huang, 2019 | The proposed intelligent technology of nursing homes is to improve the living environment and quality of life | Improving quality of life |  |  |
| 48 | Korte | New, innovative materials and technologies have made it possible to cost-efficiently design and build better living environments that allow for improved quality of life. |  |  |  |
| 49 | Wang, 2014 | The implementation of smart senior care will have positive meaning to improve the quality of life for the elderly |  |  |  |
| 50 | Baidu, 2018 | The smart senior care solution…To meet the demands on integration of medical services | Meeting older adults and users' satisfaction |  |  |
| 51 | Cui et al., 2020 | All these service elements can be designed to satisfy inhabitants expectations |  |  |  |
| 52 | MHURD | Building smart nursing home is to meet the comprehensive needs of the elderly such as diet and daily life, care, health management and entertainment |  |  |  |
| 53 | Tang et al., 2019 | There is a crucial need for adopting smart health in nursing homes so as to ...hence improve the overall satisfaction of the elderly |  |  |  |
| 54 | Cui et al., 2020 | Smart nursing homes consist of more diverse agents and elements than smart homes  Smart homes and smart nursing homes are not exactly identical, the key technologies and features are similar…  Smart nursing homes belong to smart homes and the former is just a kind of smart homes with specific users | Similar to smart home | The concept belongs to smart homes with specific users. It performs as a home-based care with the functions of both home and hospital to guarantee a better environment for older adults. | Combination of smart home and hospital model |
| 55 | Korte | Smart nursing home construction: Much like nursing home interior design and architectural design, in nursing home construction |  |  |  |
| 56 | Morley, 2012 | Bringing... “Smart Homes” including: 1. Monitor motor performance B Monitor falls; 2. Sleep pattern monitoring; 3. Monitor vital signs; 4. Monitor wandering (GPS); 5. Monitor dehydration; 6. Monitor skin wetness and skin images into nursing homes |  |  |  |
| 57 | Cui et al., 2020 | It performs as a home-based health care system with the functions of both home and hospital to some certain | Home and hospital model |  |  |
| 58 | Korte | That’s a major change from the past, when nursing homes followed the hospital model, and efficient care was the driving force behind design and construction |  |  |  |
| 59 | Gamberini et al., 2018 | There is a need to find innovative solutions to provide users with more comfortable and safe environments. Priority must be given to the promotion of a healthy and active style of lifestyle and to guarantee a degree of independence | More comfortable and safe environments |  |  |
| 60 | Cui et al., 2020 | Smart nursing home including not only elderly or disable people, but also nurses, medical workers and other caregivers | Special users-older adults and caregivers |  |  |
| 61 | Siciliano & Khatib, 2016 | The facility may have zones to separate residents who have different levels of dependency |  |  |  |

**B: Criteria of Smart NH**

| No | Authors and year | Data Extract/ Quotation | Codes | Description | Themes |
| --- | --- | --- | --- | --- | --- |
| 1 | Baidu, 2018 | Improve the level of elderly care services in the hospital  To provide intelligent services to the elderly and other care recipients in the nursing home through intelligent construction and actively explore the ways to optimize the quality of care | Provide/improve quality of care | The quality of care is the extent to which health care services provided to individuals and patient populations improve desired health outcomes. In order to achieve this, health care must be safe, effective, timely, efficient, equitable and people-centered. (WHO). | Quality of care |
| 2 | Huang et al., 2019 | Provide more accurate and efficient care services to improve the service quality |  |  |  |
| 3 | Korte | The core purpose of a senior living center — to facilitate quality care |  |  |  |
| 4 | Matusitz et al., 2013 | The rate of mortality and patient satisfaction to be indications of deficiencies in quality of care |  |  |  |
| 5 | MHURD | To improve the level and quality of institutional cares to meet the comprehensive needs of the elderly population |  |  |  |
| 6 | Tang et al., 2019 | The adoption of smart health in the area of GCM is worthy of consideration in nursing homes so as to deliver accurate and fast response healthcare services and improve the QoC |  |  |  |
| 7 | Huang et al., 2019 | In daily life mode, physiological signals and abnormal events can still be monitored for a long time | Safe |  |  |
| 8 | MHURD | Environmental monitoring system, monitoring system for personal safety, alarm and help system, monitoring system for households |  |  |  |
| 9 | Siciliano & Khatib, 2016 | Have a 24 h staffed vital signs monitoring and alert capability |  |  |  |
| 10 | Wang, 2020 | To prevent elderly fall and send alarm when the heart rate and blood pressure is abnormal, then the guardian can know in time and find solution |  |  |  |
| 11 | Xiexiebang, 2019 | Giving a quick response to the special events and ensure the safety of life to the elderly residents and their living environment |  |  |  |
| 12 | Baidu, 2018 | The goal in our hospital is to reach the intelligent, standardized and digitalized services for the elderly people | Effective |  |  |
| 13 | Betgé-Brezetz et al., 2009 | Select the right delivery mode taking into account criteria such as the semantics of the information to be notified, its priority and criticality, the end-user preferences and disabilities (limitations), their contexts, or the device capabilities |  |  |  |
| 14 | Cui et al., 2020 | Operation records like health records are stored for further use in accordance with regulations…  Some complex systems can be described and measured by the computational simulation during architecture design |  |  |  |
| 15 | MHURD | There is no standalization to a smart nursing home, the opration should follow the standards to each section and referring the technologies |  |  |  |
| 16 | Shenghuo, 2020 | Ensure all technologies and systems operating continuingly in 24 hours |  |  |  |
| 17 | Tang et al., 2019 | To extend this concept for improving the effectiveness, efficiency, reliability and accuracy in executing the GCM in nursing homes |  |  |  |
| 18 | Baidu, 2018 | Constructing the hospital IoT sensing system and real-time security protection system, are able to identify the data and behavior of the elderly  It could improving the efficiency of care service and reducing caregivers workload | Efficient |  |  |
| 19 | Cui et al., 2020 | The real-time and realistic data collected by the sensors will be analyzed by data mining technologies, which can help smart nursing home run better |  |  |  |
| 20 | SheCuiTong | Improving working and management efficiency of elderly care institutions, reducing resource waste, and enhancing core competitiveness |  |  |  |
| 21 | Siciliano & Khatib, 2016 | To better serve residents and guests to optimize function and to minimize cost to improve safety and efficiency |  |  |  |
| 22 | Tang et al., 2019 | Adopting smart health in nursing homes so as to deliver accurate and fast responses in healthcare services…  To improve the efficiency and reliability of daily routines and care plan modification processes  IoMT application in the nursing home provides advantages for caregivers to capture and monitor relevant data in real- time |  |  |  |
| 23 | Xiexiebang, 2019 | Staff can accurately monitor and manage the living and health conditions in real time for the elderly people |  |  |  |
| 24 | Cui et al., 2020 | Smart nursing homes often aim to make every resident satisfied according to individual requirements, characters, and feedbacks | People-centered (PC) |  |  |
| 25 | Huang et al., 2019 | Centered on the elderly to manage nursing home in order to realize the intelligent elderly care and build a human-centraled environment |  |  |  |
| 26 | Korte | New technology that’s custom-made for care settings is improving staff communication and care |  |  |  |
| 27 | MHURD | Regulations, responsibilities, service standards and procedures for the operation and maintenance of intelligent systems should be developed to address the special care characteristics for the elderly people |  |  |  |
| 28 | Telpo | We offer the customization and the voice SDK for partners |  |  |  |
| 29 | Wang, 2014 | We test physical condition of the elderly in 24 hours a day, and the detected data will be transmitted to processing center for real-time analysis through the network |  |  |  |

^a^ NH=Nursing home

^b^ IoT=Internet of things;

^c^AI= Artificial intelligence
