# Supplementary material for: Defining the concepts of a smart nursing home and its potential technology utilities that integrate medical services and are acceptable to stakeholders: a scoping review
Source: BMC Geriatr. 2022 Oct 7;22:787. doi: 10.1186/s12877-022-03424-6 (PMC9540152; doi:10.1186/s12877-022-03424-6)
Supplement: Supplementary file 5 — Additional file 5. The Code Sheet of Integration of Medical Services. [file 12877_2022_3424_MOESM5_ESM.docx]

**Supplementary file 5: The Code Sheet of Integration of Medical Services**

| No. | Authors and year | Quotation | The form of integrated medical services | Sub-codes | Codes |
| --- | --- | --- | --- | --- | --- |
| 1 | Armer et al., 2004 | The University of Missouri-Columbia is a participant in the MTN, a video-conferencing network that extends to 23 sites throughout the state connected by dedicated T1 lines. Three of the participating counties were chosen as pilot test sites for the implementation of another type of telemedicine technology—the computer.   The nursing home did not have a video-conferencing system on the grounds, although such a system was accessible through the local hospital. | Telemedicine and videoconferencing or without videoconferencing | Teleconsultation and videoconferencing | Integration of medical services in telemedicine |
| 2 | Daly et al., 2005 | Live video and detailed images of nursing home residents can be transmitted in real time via the Internet. | Teleconsulting, live video and image transition |  |  |
| 3 | Chan et al., 2001 | A residential nursing home in Hong Kong was linked to the community geriatric assessment team based in a regional hospital using videoconferencing. It produced a strong partnership between the nursing home and regional hospital. | Videoconferencing and teleconsultation |  |  |
| 4 | Hui & Woo, 2002 | Identical videoconferencing units (Viewstation, Polycom) were installed at the nursing home and Shatin Hospital. A high-resolution portable camera (Samsung) was introduced subsequently which allowed better visualization of lesions. A computer was connected to the Viewstation at the hospital site for downloading clinical photographs. Participated in the study, using real-time teleconsultation instead of face-to-face consultations. |  |  |  |
| 5 | Newbould et al., 2017 | Twelve homes reported using videoconferencing. |  |  |  |
| 6 | Rabinowitz et al., 2010 | Providing psychiatric care to rural nursing home residents by videoconference is cost effective and appears to be a medically acceptable alternative to face-to-face care. |  |  |  |
| 7 | Schneider et al., 2016 | Telemedicine care was provided using videoconferencing, which requires a minimum of 512 Kbps bandwidth for audio and high-definition video on a desktop computer. |  |  |  |
| 8 | Toh et al., 2015 | Telegeriatrics helped to bridge the physical distance between the NH^a^ resident and the hospital’s geriatrician, by delivering geriatric medical care to two partnering NHs via videoconferencing. |  |  |  |
| 9 | Weiner et al., 2001 | The system includes a rolingcart with video conferencing hardware and software, a remotely controllable digital camera, light, wireless network, and battery. A semi-automated paging system informs physicians of patient’s study status and indications for conferencing. Data transmission occurs wirelessly in the nursing home and then through Internet cables to the physician’s home. |  |  |  |
| 10 | Weiner et al., 2003 | We conducted a clinical trial of unscheduled, night-time videoconferencing in a nursing home, where on-call physicians usually provide care by telephone from remote locations. |  |  |  |
| 11 | Biglan et al., 2009 | We subsequently switched to VSee Video-conferencing freeware in January 2008. For all visits, we used an encrypted high-speed internet connection and a Logitech Digicam (camera and microphone combined). A nursing home staff member and technical support staff were available at each visit to provide assistance. | Videoconferencing and telemedicine |  |  |
| 12 | Grabowski & O'Malley, 2014 | The intervention consisted of introducing into the nursing home a cart with equipment for two- way videoconferencing and a high-resolution camera for use in wound care. When a nursing home resident had an off-hours medical problem, a staff member brought the cart into the resident’s room and contacted the telemedicine service. |  |  |  |
| 13 | Pallawala & Lun, 2001 | The NHs, government hospital and research university are connected via ADSL protocol, which support high bandwidth, which is necessary for high quality videoconferencing. Each time a patient needs a teleconsultation, a nurse or doctor in the remote site sends the history to the EMR server. EMR server forwards the request to the Alexandra Hospital for consultation. Geriatrics specialists at Alexandra Hospital carry out teleward rounds twice weekly and on demand basis | Videoconferencing, teleconsultation and EMR^b^ |  |  |
| 14 | Savenstedt et al., 2004 | Videophones have been used to facilitate tele-consultations between nurses and elders at a nursing home. | Videophones and teleconsultation |  |  |
| 15 | Cusack et al., 2008 | The Center for Information Technology Leadership has examined the value of telehealth encounters in which there is a provider both with the patient and at a distance from the patient. We considered three models of telehealth: store-and-forward, real-time video and hybrid systems. | Store-and-forward, real-time video, hybrid systems and teleconsultation |  |  |
| 16 | Catic et al., 2014 | Long-term care sites presented challenging cases regarding residents with dementia and/or delirium related behavioral issues to specialists via video-conferencing. HIPAA compliant video-consultation technology was used to facilitate longitudinal co-management of patients between the specialty team. | Video-consultation technology and teleconsultation |  |  |
| 17 | Chang et al., 2010 | Patients use remote controls and touch panels for uploading their daily biometric information into the database. Physicians read this information using their mobile phones and real‐time medical advice is therefore provided. Subsequently, patients and their physicians engage in an interactive video conference every week in order to discuss the data analysis results and methods for improving the patients’ health. | Telemonitoring plus teleconsultation via videoconferencing | Telemonitoring |  |
| 18 | De Luca et al., 2016 | Each node of the telemedicine system used for patient monitoring consisted of a box that was connected to the monitor of a personal computer (PC) via VGA cable. The data obtained from the measurement of vital parameters were transmitted from the telemedicine devices to the box via Bluetooth or wireless technology. Indeed, once the box was installed and activated, and the telemonitoring device configured, the user could access and manage the box through a remote control. The system automatically transmitted the recorded data to our telemedicine center by using the local internet connection, without interaction of the involved subjects. | Telemonitoring and teleconsulting |  |  |
| 19 | Pallikonda Rajasekaran et al., 2010 | The sensor grid architecture also provides various medical organizations and physicians across the world with access to the medical records of patients in order to refer and/or consult some critical case with some other doctor or physician who is far away from the patient site, and also enables them to come together to share resources and collaborate with each other in order to achieve efficient medical care. | Shared health information collected by wireless Sensor Networks (WSNs) and telemonitoring |  |  |
| 20 | Zhang, 2017 | Health management system includes 5 components: 1) health monitoring system, 2) portable health monitoring devices, 3) smart watch, 4) health information platform, 5) app | Telemonitoring, wearable devices and web-based health information through an App |  |  |
| 21 | Delmastro et al., 2019 | Personalised monitoring and rehabilitation services for older people, based on mobile and wearable technologies. It also provides a complete combined care approach through integrated data analysis tools applied to medium and long-term monitoring of heterogeneous health conditions. | Telemonitoring and wearable devices |  |  |
| 22 | Deng, 2019 | The intelligent nursing refers to using Internet of Things, wearable technology and mobile technology to provide the elderly with real-time, efficient and intelligent services. |  |  |  |
| 23 | Wang, 2014 | In nursing homes, the elderly wearing the RFID smart bracelet which can capture the life characteristics information, considering on the spiritual needs of the elderly, we will provide intelligent mobile robot for them so that they can chatting online with their family meantime, the cameral can be used to collect the action information. |  |  |  |
| 24 | Mishkhal et al. 2020 | A multiple wearable triaxle accelerometer sensor W2ISP that can be connected to a single RFID sensor, where RFID used the air interface protocol to communicate with the W2ISP. |  |  |  |
| 25 | Vowden & Vowden, 2013 | The wound diagnosis made by the care-home staff, the state of the wound and information on pain, exudate levels and treatments were recorded. Additional forms could be submitted at any time. Wound images were taken using a smartphone camera and these data were electronically linked to the digital paper form. Once all the data were collected, the wound images and information were uploaded together to a secure server for assessment by the remote nurse consultant. The evaluation homes received standard care supported by input from the remote experts. | Teleconsultation without videoconference (by digital documents) | Teleconsultation and information technologies |  |
| 26 | Zelickson, 2003 | A nurse collected and sent the histories and images using the teledermatology system. A diagnosis and treatment plan was determined by examine a transmitted still image and patient history alone and in combination by 2 to 3 dermatologists independently. An independent dermatologist made an on-site dermatologic consultation within 2 days after the images had been collected. |  |  |  |
| 27 | Janardhanan et al., 2008 | Email messages were sent automatically to dermatologists when a new patient was referred and to nurses when a diagnostic report was ready. A hard copy of the diagnosis/treatment report was sent to the matron and then to the resident doctor Who reviewed it and prescribed the suggested medications. In the event of relapse, recurrence or no progress, the respective patient cases were uploaded into DPHIMS again for follow-up diagnosis/treatment. They also provided the treatment plan if available along with suggestions, comments or recommendations. | Internet (or email) and teleconsultation |  |  |
| 28 | Low et al. 2020 | The use of a simple videoconferencing (VC) system, which is considered old technology, is highly feasible and could have a significant impact on the care of patients in LTC facilities. This is made possible by the advancement in mobile broadband and wireless technology which allows for the transmission of vast amounts of data across long distances with speed and precision never seen before, translating real time video and audio signals into clear and accurate pictures on the high-resolution monitor. |  |  |  |
| 29 | Lavanya et al., 2006 | The diagnosis and treatment recommendations made by the dermatologists using the D-PHIMS diagnosis module were effective in most cases based on feedback from the nursing staff at the elderly nursing home. | Personal health information management system (D-PHIMS) and teleconsultation |  |  |
| 30 | Doumbouya et al., 2015 | 1) A medical professional cannot make a decision, he can make a teleexpertise request to remote medical professionals for advices. 2)This will make it possible to visualise and understand easily the already taken decisions since all is represented in conceptual graphs, which can be manipulated with little or no effort. 3)Results revealed that with the proposed system of teleexpertise there are substantial increases in the number of collaborative processes and answers, percentage of accurate diagnoses, application of reasoning strategies, and reduction of errors. The system facilitates collaboration for better decision-making provided by specialists with better skills and experiences. | Remote specialists and teleconsultation for decision making | Teleconsultation and remote specialist decision making |  |
| 31 | Shafiee Hanjani et al., 2019 | Telehealth platform and service developed to deliver geriatrician-led comprehensive geriatric assessment (CGA) to aged care residents in Queensland, Australia. Geriatricians have online access to a complete clinical profile of residents prepared by an experienced nurse at the residential aged care facility using the interRAI long-term care facility (LTCF) assessment instrument and conduct weekly video conference consultations to any new or existing resident in need. | Telehealth and interprofessional collaboration |  |  |
| 32 | Liu & Hsu, 2014 | The design concept of BCTS is to integrate telehealth functions into something that already exists in the home and nursing homes. The core sensor of the BCTS is a soft motion sensing mattress, WhizPAD. The BCTS facilitates bed-related real- time monitoring (on/off bed status, sleep posture, body movements), service reminder, and historical data record. Caregivers can also use mobile devices to access the data collected by WhizPAD. | mhealth (App) and a soft motion-sensing mattress | mHealth and abnormal event monitoring | Integration of medical services through mHealth |
| 33 | Mendes et al., 2017 | In this sense, Multi-Agent Systems (MAS) can provide a suitable approach to face this challenge, where several autonomous computing entities can retrieve and perform distributed analysis of the individuals’ data in a real-time and personalized manner, and act as personal assistants, also interacting with other individuals’ agents and health professionals, such as physicians, nurses and gerontologists... This paper describes an innovative healthcare approach that combines MAS and data analysis techniques to monitor and characterize the daily activities and physiological conditions of a group of elderly people institutionalized in a nursing home. | Wearable devices and m-health personalised monitoring |  |  |
| 34 | Delmastro et al., 2018 | The use of mobile and e-health personalised services at home and in residential long-term care facilities can help to stabilise the health conditions of the subjects, in terms of physical, mental, and social capabilities. In this context, we propose a set of personalised monitoring and rehabilitation services based on mobile, wearable, free contact, and touch screen technologies designed to provide an integrated care and monitoring programme for elderly frail subjects. | Mobile and e-health personalised monitoring services |  |  |
| 35 | Donnelly et al., 2018 | The reviewed clinical data generated by mobile and wearable devices and reflected upon their trial-related experiences. | Mobile and wearable devices |  |  |
| 36 | Montalto et al., 2015 | RACFs and general practitioners should use mobile X-ray and integrate these services into their management of aged care delivered in RACFs. The service was provided by a hospital using a single vehicle, staffed by a radiographer. It accepts referrals by telephone and facsimile, and travels to nursing homes to deliver services. The vehicle contains a portable plain X-ray machine, a digital processor, a compact disc (CD) burner and a printer–scanner, as well as a computer with mobile broadband capable of processing images and sending digital images for reporting and storing. The X-ray service (MXS) has a relationship with a radiology service for reporting images, and is responsible for reporting, sending results and storing images. | Teleconsultation and mobile X-ray | mHealth and point-of-care |  |
| 37 | Dozet et al., 2016 | With the use of the digital detector with instant read-out, it was possible for the radiographer to evaluate quality of positioning and image exposure on site. The images were transmitted from the mobile radiography unit to a router in the transporting van, which relayed the images via mobile transmission to the hospital’s Picture Archiving and Communication System. | mHealth, radiography unit and digital detector |  |  |
| 38 | Esteves et al., 2019 | This project emerged and consists in designing and developing a mobile solution that would help and assist the health professionals of a Portuguese nursing home at the point-of-care. | Mobile and point-of-care |  |  |
| 39 | Wälivaara et al., 2011 | For patients living far from the health care centre and where home visits are not possible, the GPs saw MDST as helpful in supporting decisions. | Teleconsultation and mobile distance-spanning technology (MDST) | mHealth and teleconsultation |  |
| 40 | Crotty et al., 2014 | Telerehabilitation employed a coaching model, with fewer therapist home visits, more feedback and ‘‘homework’’ for the patient. Patients had a tablet computer loaded with a videoconferencing app to connect with therapists and relevant therapeutic apps. | Teleconsultation via videoconferencing and web-based health information through an App |  |  |
| 41 | Lai et al. 2020 | Near VA and contrast sensitivity were measured with Paxos Checkup, a smartphone-based near visual acuity testing application. Personel trained on Pax’s Checkup conducted all assessments with patients’ own corrective lenses or a +2.5 diopter lens held in front of the eye as needed. Anterior and posterior segment photographs were taken with the Paxos Scope ophthalmic camera system coupled to an iPhone. Anterior and posterior photographs were reviewed and graded remotely by an ophthalmologist masked to patient identifiers. Smartphone-acquired data were uploaded to a secure HIPAA- compliant server. | Smartphone-based teleophthalmology platforms |  |  |
| 42 | Alexander 2008 | The OneTouch system provided point-of-care technology that enabled healthcare providers to access and enter resident information outside of confined nursing stations. Few nursing homes have incorporated integrated clinical information systems into their clinical practices to support clinical practice, enhance clinical support between different providers (laboratory, physical therapy, social services, etc.), and provide better administrative oversight. | Information management and clinical practice in different care departments | Clinical information integration | Integration of clinical information |
| 43 | Alexander et al., 2015 | Resident care information systems were also integrated with Physical Therapy and Occupational Therapy (PT/OT) disciplines to a greater extent than other resident care disciplines (e.g. pharmacy, dietary, and laboratory). |  |  |  |
| 44 | Ohol, 2010 | The web-based EHR system at TigerPlace is an integrated set of a comprehensive EHR project that manages health care and personal data for residents at the facility. The system is tightly linked with other clinical systems used at the TigerPlace and facilitates the capability to share data with these systems. | Electronic health record and technology-based devices |  |  |

^a^ NH=Nursing home

^b^ EMR=Electronic medical records
