# Supplementary material for: Defining the concepts of a smart nursing home and its potential technology utilities that integrate medical services and are acceptable to stakeholders: a scoping review
Source: BMC Geriatr. 2022 Oct 7;22:787. doi: 10.1186/s12877-022-03424-6 (PMC9540152; doi:10.1186/s12877-022-03424-6)
Supplement: Supplementary file 6 — Additional file 6. The Code Sheet of Stakeholders’ Acceptability. [file 12877_2022_3424_MOESM6_ESM.docx]

**Supplementary file 6: The Code Sheet of Stakeholders’ Acceptability**

| No. | Authors and year | Data Extract/ Quotation | Sub-codes | Description | Codes | Theme |
| --- | --- | --- | --- | --- | --- | --- |
| 1 | Huang et al., 2013 | Willingness was greater among those suffering from heart disease | Severity of illness | The attributes of elderly people to the acceptability include the severity of illness and other individual demographic variables. | Attributes of residents | Attributes of residents and HCPs^a^ |
| 2 | Armer et al., 2004 | The nursing home staff and advanced practice nurses with higher education voiced greater understanding of potential applications of technology in the practice of nursing | Education attainment | The identified attributes of HCPs include education attainment, clinical working experience and the level of tech-savvy. | Attributes of HCPs |  |
| 3 | Handler et al., 2013 | Physicians with 15 or fewer years of clinical experience were 67% more likely to be mobile device users, compared with those with more than 15 years of clinical experience  There was a significant negative association between duration of practicing clinical medicine and mobile device use, with those who recently completed their clinical training showing greater | Clinical working experience |  |  |  |
| 4 | Betgé-Brezetz et al., 2009 | Usability was clearly related to the habit and easiness of nurses to use a PDA (better for young nurses) | The level of tech-savvy |  |  |  |
| 5 | Handler et al., 2013 | Younger physicians may be more familiar with mobile devices and have already integrated them into their workflow |  |  |  |  |
| 6 | Janardhanan et al., 2008 | Nurse in Phase 1 without computer experience said that it was difficult to use |  |  |  |  |
| 7 | Abbate et al., 2014 | After some convincing story about the importance of wearing the devices, AD individuals eventually wore and benefited from the monitoring technologies | Awareness from external resources | The external information that the user received are from HCPs, friends, family members, and media sources (Golant, 2017). | Persuasiveness of external information | Coping process and technology appraisals |
| 8 | Chang et al., 2009 | Most respondents had heard about telemedicine before participation and showed some interest |  |  |  |  |
| 9 | Eklund et al., 2012 | In all instances, there was a good patient acceptance of the radiographic procedure, and the 62 patients who were able to answer the questionnaire gave a favorable response. Examples of the three most common answers from the patients were ‘happy about not having to go away’, ‘feeling safe’, ‘much better than at the hospital’ | User experience of received benefit from using a new technology | People acquire internal information by remembering personal experiences from their earlier experiences and satisfaction. | Persuasiveness of internal information |  |
| 10 | Huang et al., 2015 | The majority of the 51 participants believed the 6-inch mHealth device was helpful for recording and reporting information about their health, easy to use, and easy to learn; in addition, the NH^b^ residents enjoyed using the device |  |  |  |  |
| 11 | Chang et al., 2012 | After the experiment, user satisfaction was investigated to understand the intended use of the WSN healthcare monitoring system | Achievement of user’s satisfaction |  |  |  |
| 12 | Weiner et al., 2003 | Physicians and nursing home residents were satisfied with videoconferencing when it did occur, and physicians reported that making medical decisions was easier |  |  |  |  |
| 13 | Yu et al., 2008 | Caregivers at both homes were satisfied with their homes’ documentation system, and had positive attitudes towards using electronic documentation systems.   After five weeks’ experience of using the electronic system for daily nursing documentation, the caregivers at the electronic site had a similar high level of satisfaction with their home’s documentation system to that of their counterparts who used paper-based documentation |  |  |  |  |
| 14 | Zelickson, 2003 | The dermatologists felt comfortable in making a diagnosis and treatment plan in all cases in which they had access to both the image and patient history. Only 23% of the patients or guardians returned the satisfaction survey, the majority were happy with the experience. Patients' relatives and resident nurses were satisfied with the teledermatology system studied |  |  |  |  |
| 15 | Betgé-Brezetz et al., 2009 | Regarding the residents, they find the phone call through the TV very useful. The users appreciated the adapted and personalized delivery of information and the friendliness of the system for the help request | Usefulness | The perceived efficaciousness of smart technologies was linked to the perceived usefulness, performance expectancy, relative advantage and pleasure experience by the users which was instrumental in achieving medical outcomes and meeting personal demands (Golant, 2017). | Perceived efficaciousness |  |
| 16 | Bleda et al., 2018 | According to the staff experience: new residents can be easily registered into the system. Later they can doublecheck the name of the residents that are sleeping in a given room. They find this feature very useful in order to prevent errors  The residence staff considers that the functionality for monitoring and tracking alerts is very useful. There have been shift changes and the incoming caregiver needs to know if an alert has been attended. The interface shows this information immediately and the caregiver need has been solved |  |  |  |  |
| 17 | Delmastro et al., 2019 | As far as the nursing care personnel is concerned, they found the system useful and a good support for the daily routine |  |  |  |  |
| 18 | Qadri et al., 2009 | Nurses are very receptive to the use of hand-helds containing point-of-patient-care information. Nurses agreed on the utility of the tools for scheduling purposes and for the potential for assisting them in caring for their residents |  |  |  |  |
| 19 | Savenstedt et al., 2002 | Both the doctor and the nurses participating in the study stated that they found teleconsultations useful in managing most problems |  |  |  |  |
| 20 | Wälivaara et al., 2011 | General practitioners’ reasoning about using mobile distance-spanning technology: The results show quite a few expressions about the MDST as useful and valuable in health care at home and in nursing home settings |  |  |  |  |
| 21 | Alexander et al., 2007 | Overall, licensed nurses viewed technology as helpful...Administrative and licensed staff across all facilities saw this as a system benefit. | Helpfulness and improvement in care efficiency |  |  |  |
| 22 | Alexander et al., 2015 | Participants (NH caregivers) emphasized that the use of HIE would be a more efficient means of communication |  |  |  |  |
| 23 | Handler et al., 2013 | NH physicians who use mobile devices equipped with drug reference software believe they are helpful for reducing ADEs |  |  |  |  |
| 24 | Janardhanan et al., 2008 | The dermatologists felt that DPHIMS was helpful in obtaining specialist care for the residents |  |  |  |  |
| 25 | Qadri et al., 2009 | 5 themes related to nurses’ acceptability: 1) Helpfulness, 2)Saving time, 3)Providing guidance, 4) Facilitating communication Convenience,5) Effectiveness |  |  |  |  |
| 26 | Chan et al., 2001 | All professionals thought that telemedicine could replace many of the on-site visits | A better solution in administrative procedures |  |  |  |
| 27 | Rabinowitz et al., 2010 | Family members also embraced the technology—none were opposed to its use, and many thought it was a creative solution to an otherwise insoluble problem |  |  |  |  |
| 28 | Weiner et al., 2003 | Physicians reported that making medical decisions was easier with videoconferencing |  |  |  |  |
| 29 | Crotty et al., 2014 | Service clinicians were equally satisfied with telerehabilitation using videoconferencing compared to face-to-face sessions. They felt it was superior to the service that they could have provided by telephone and mainly equivalent or better than a scheduled home visit (78%) | Improvement in quality of care |  |  |  |
| 30 | Handler et al., 2013 | NH physicians who have integrated a mobile device into their prescribing workflow perceive that the technology improves medication safety |  |  |  |  |
| 31 | Lavanya et al., 2006 | Both dermatologists agreed that there was an overall improvement in the quality of healthcare after using D- PHIMS… All the nurses (100%) agreed that they found an overall improvement in the quality of healthcare after using D- PHIMS |  |  |  |  |
| 32 | Pallawala & Lun, 2001 | Nursing staff feels that the system has reduced the transfers. Nurses were enthusiastic about the prospect of practicing in this way. They also feel that their management of patients has improved significantly and that they are confident in an event of emergency |  |  |  |  |
| 33 | Qadri et al., 2009 | The nurses in long-term care were anxious to utilize portable and easily accessible information related to improving their care of more challenging residents. Nurses participants adored the pocket PC because of its larger information potential and the versatility of features that could assist them in organizing, implementing, and enriching their daily routines |  |  |  |  |
| 34 | Vowden & Vowden, 2013 | Nursing-home staff found the system of value and many were keen to see the trial continue to form part of routine patient management. Once familiar with the equipment, staff found the remote support beneficial |  |  |  |  |
| 35 | Eklund et al., 2012 | The local staff in the nursing homes were in all 123 cases positive. They pointed out three main beneficial factors: the security and comfort for the patients | Assurance of quality of life |  |  |  |
| 36 | Singh et al., 2017 | All the participants agreed that assistive technologies and AAL solutions can have beneficial effects on quality of life and health |  |  |  |  |
| 37 | Eklund et al., 2012 | Respondents also agreed that telemedicine may help avoid transfers to the emergency room or hospital and improve access to appropriate resident care | Improvement of healthcare accessibility and availability | The perceived usability includes effort expectancy, perceived ease of use, or perceived behavioral control (Golant, 2017). The usability appraisals depend on the availability or accessibility of these options, necessary for care, easy to understand, learn and use, affordability, compatible, the availability of tech-support during having difficulties of using a product, and “human-centric” designs such as matching preferences of users, portable and enjoyable to use. | Perceived usability (positive) |  |
| 38 | Toh et al., 2015 | The users expressed general acceptance of Telegeriatrics in providing geriatric care. They were aware of its limitations and challenges, but also recognized it as a promising way of providing consultation and strengthening nursing skills | Necessity for care |  |  |  |
| 39 | Tseng et al., 2013 | The study results also address the positive and significant impact of facilitating condition on user intention in the sense that the more capability that users consider having to use the system and related sources, the higher behavior tendency to use |  |  |  |  |
| 40 | Huang et al., 2015 | The majority of the 51 participants believed the 6-inch mHealth device was helpful for recording and reporting information about their health, easy to use, and easy to learn… the NH residents enjoyed using the device | Easy to use |  |  |  |
| 41 | Janardhanan et al., 2008 | The majority of the nurses (4/5) in Phase 1 and all of them (9/9) in Phase 2 agreed that the DPHIMS interface was easy to use… All the nurses said they would readily recommend DPHIMS to other nurses |  |  |  |  |
| 42 | Lavanya et al., 2006 | In general, both found the D-PHIMS interface easy to use and satisfied with the overall concept and system performance |  |  |  |  |
| 43 | Huang et al., 2013 | Nurses and managers liked using this system from the start, because they felt that it was very user-friendly | User-friendly |  |  |  |
| 44 | Lavanya et al., 2006 | They commented that the information provided by the D-PHIMS for any patient was comprehensive and the digital images of the patient’s skin conditions were sufficient for the diagnosis of chronic skin conditions. With their level of computer literacy, they found the interface user-friendly |  |  |  |  |
| 45 | Yu et al., 2008 | The majority of caregivers who participated in the study were comfortable with electronic documentation and felt that they worked in a user friendly, supportive environment |  |  |  |  |
| 46 | Crotty et al., 2014 | Qualitative analysis of patient, family and carer interviews suggested that telerehabilitation was acceptable and perceived positively by older people. They found it convenient, coped well with the tablet computer and the wearable activity monitor, and developed positive relationships with therapists | Convenience |  |  |  |
| 47 | Hui & Woo, 2002 | 96% of resident respondents: they felt comfortable with this mode of consultation and found it more convenient than having to travel to the clinic. Despite some increase in workload, nursing home staff felt that telemedicine increased their confidence in caring for residents |  |  |  |  |
| 48 | Abbate et al., 2014 | A device comes in one’s favourite colors, it is easier to make it acceptable | “Human-centric” designs to fit user lifestyles |  |  |  |
| 49 | Borelli et al., 2019 | Users’ Opinion About Acceptability to the Smart Objects:Wall light for indoor localization (Easy to install, does not require much maintenance and adaptable to different domestic context) ; Armchair for sitting posture monitoring (Excellent comfort and easy to personalize changing materials or colors); Belt for movement information (Pleasantness to wear and on tactile feel); Wall Panel and mobile devices as user interface (Pleasantness to the visual perception and easy to personalize with different colors and textures) |  |  |  |  |
| 50 | Hui & Woo, 2002 | Telemedicine was cheaper than conventional care, and well accepted by health-care professionals as well as clients | Affordability |  |  |  |
| 51 | Rabinowitz et al., 2010 | Some patients who had training or careers in telecommunications or related fields found the videoconference approach interesting, and some were curious and asked technical questions | Adequate tech-support and regular training |  |  |  |
| 52 | Yu et al., 2008 | Caregivers’ fears about this new practice and increased their confidence in using computers. The majority of caregivers would accept electronic nursing documentation if sufficient training and support were given |  |  |  |  |
| 53 | Gaglio et al., 2016 | Our findings show different levels of domestication: for some of the nursing homes, the lack of practical relevance of the toolkit in emergencies and the difficulty to borrow artifacts from doctors prevented complete adoption. For three nursing homes, domestication occurred in an unexpected way in the sense that the objective of the domestication changed | Appropriate domestication of a new technology |  |  |  |
| 54 | Lavanya et al., 2006 | The medical care received using D-PHIMS (e-Health) was not as good as a regular clinical visit | Unusefulness | The negative perceiveness to the usability appraisals | Perceived usability (negative) |  |
| 55 | Huang et al., 2015 | 50% of the respondents were uncertain about the user benefits, were not convinced that it was reliable for data entry, and did not wish to continue to use such a device | Uncertainty of usefulness |  |  |  |
| 56 | Fraile et al., 2010 | The acceptance among the nurses was 88%, despite the initial learning phase. Nurses, who tried the system, demonstrated a certain rejection to the use of PDA, as well as to the training period required to learn the system | Not easy to learn |  |  |  |
| 57 | Delmastro et al., 2019 | This device has not been positively accepted by the users, mainly due to its management issues (i.e., battery daily recharge, continuous wearing) | Not easy to use |  |  |  |
| 58 | Alexander, 2005 | Important factors shaping use of the system included staff perception of how the system functioned, resources available to assist with trouble-shooting of system problems, equipment availability, and preparation for the process change | The difficulty of resources availability and accessibility |  |  |  |
| 59 | Shafiee Hanjani et al., 2019 | However, they also identified some challenges in the process and disadvantages with the system. There were some issues around accessibility and adequacy of provided clinical information |  |  |  |  |
| 60 | Byrne, 2005 | Clinical support tools for nurses, especially LTC nurses, have been limited and slow to be adopted, due to many factors, such as low investment in IT and computer training, and the lack of useful, high-quality clinical decision-support applications relevant to the nursing home population | Lacking in supportive resources or tech-support |  |  |  |
| 61 | Alexander et al., 2007 | In all facilities, unlicensed staff viewed the technology as increasing their accountability and workload, while reducing their time with residents. The newly automated documentation and messaging systems increased frustrations among both licensed and unlicensed staff, with the latter documenting more information than they had in the active | Burden of using technology |  |  |  |
| 62 | Huang et al., 2013 | Putting the probe on the patients caused extra work for the care givers. They also needed to regularly move it to another finger to prevent pressure sores. Thus the care givers did not like this system very much when they started using it |  |  |  |  |
| 63 | Toh et al., 2015 | The geriatricians also articulated that not seeing the patients in person and their heavy reliance on the nurses were potential medical risks | Potential medical risks | The collateral damages refer to the unintended and harmful damages that elderly people worry will result if they adopt smart options or not. These might include a change in their usual lifestyles, perceived threats to locus of control of one personal lives and environments (Golant, 2017). | Perceived collateral damages |  |
| 64 | Huang et al., 2015 | They also believed that they were more likely to make errors during data entry. These problems emphasize that sensitivity to the utility of a mobile device | Sensitivity of technology and errors during the operation |  |  |  |
| 65 | Chang et al., 2009 | More caregivers were concerned about increased costs (p=0.020), poor hardware quality (p<0. 001), poor security, confidentiality, and reliability (p = 0.036), inconvenience to patients (p = 0.006), associated moral and ethical issues (p = 0.006), and uncertainty about responsibility (p = 0.022). The two groups did not differ in expectations concerning benefits of telemedicine | Overall concern of technology |  |  |  |

^a^ HCPs=Healthcare professionals

^b^ NH=Nursing home
